# Uncovering and resolving challenges of quantitative modeling in a simplified community of interacting cells

**DOI:** 10.1371/journal.pbio.3000135

**Published:** 2019-02-22

**Authors:** Samuel F. M. Hart, Hanbing Mi, Robin Green, Li Xie, Jose Mario Bello Pineda, Babak Momeni, Wenying Shou

**Affiliations:** 1 Division of Basic Sciences, Fred Hutchinson Cancer Research Center, Seattle, Washington, United States of America; 2 Department of Biology, Boston College, Boston, Massachusetts, United States of America; Yale University, UNITED STATES

## Abstract

Quantitative modeling is useful for predicting behaviors of a system and for rationally constructing or modifying the system. The predictive power of a model relies on accurate quantification of model parameters. Here, we illustrate challenges in parameter quantification and offer means to overcome these challenges, using a case example in which we quantitatively predict the growth rate of a cooperative community. Specifically, the community consists of two *Saccharomyces cerevisiae* strains, each engineered to release a metabolite required and consumed by its partner. The initial model, employing parameters measured in batch monocultures with zero or excess metabolite, failed to quantitatively predict experimental results. To resolve the model–experiment discrepancy, we chemically identified the correct exchanged metabolites, but this did not improve model performance. We then remeasured strain phenotypes in chemostats mimicking the metabolite-limited community environments, while mitigating or incorporating effects of rapid evolution. Almost all phenotypes we measured, including death rate, metabolite release rate, and the amount of metabolite consumed per cell birth, varied significantly with the metabolite environment. Once we used parameters measured in a range of community-like chemostat environments, prediction quantitatively agreed with experimental results. In summary, using a simplified community, we uncovered and devised means to resolve modeling challenges that are likely general to living systems.

## Abstract 2

An attempt to model the behaviour of a cooperative community of two engineered yeast strains shows that quantitative modelling of even a simplified living system requires a Herculean effort for parameter quantification.

## Author summary

A crown jewel of any scientific investigation is to make accurate and quantitative predictions based on mechanistic understanding of a system. Although quantitative prediction has been the norm and the expectation in physical sciences, living systems are notoriously difficult to predict quantitatively. One of the major challenges is obtaining model parameters. Choosing model parameters to fit data often results in a model that can explain the fitted data but not predict new data. When modeling cells, “guessing” phenotype parameters or “borrowing” parameters from a different genetic background can be highly problematic. In addition, phenotype parameters can vary significantly over time, as cell physiology changes with changing environments, or as cells evolve rapidly. Thus, although parameters are assumed to be constant in most models, this is a far cry from reality and may interfere with quantitative prediction. Here, using a simple engineered yeast community as our case example, we demonstrate that quantitative modeling is possible, but only after overcoming multiple difficulties in parameter measurements. Our approach should be generalizable for modeling communities of interacting cells for which genetic or chemical information of interaction mechanisms is available.

## Introduction

Successful prediction of quantitative traits of a biological system can be tremendously useful. For example, if we can quantitatively predict properties of microbial communities, then we will be empowered to design or manipulate communities to harness their activities [1–6], ranging from fighting pathogens [7] to industrial production of vitamin C [8,9].

An important community-level property is community dynamics, including how species concentrations change over time [6]. Community dynamics can be predicted using statistical correlation models. For example, community dynamics observed over a period of time can be used to construct a model that correlates the concentration of one species with the growth rate of another, and the model can then be used to predict future dynamics [10–12]. However, even for two-species communities, statistical correlation models might generate false predictions on species coexistence [13].

Alternatively, mathematical models can be constructed based on species interaction mechanisms, such as how metabolites released by one species might affect the growth of another species. For example, genome-scale metabolic models use genome sequences, sometimes in conjunction with RNA and protein expression profiles, to predict metabolic fluxes within species as well as metabolic fluxes among species (i.e., metabolic interactions) [14,15]. However, these models face multiple challenges, including unknown protein functions or metabolic fluxes [16].

When interaction mechanisms are known [14,17–21], we can construct a model based on interaction mechanisms. Ideally, we would use the model to first determine which parameters are critical for the phenomenon of interest, and then directly quantify those critical parameters. However, parameter quantification can be time-consuming. Thus, in many models, a fraction of model parameters are “free parameters” (unmeasured parameters that can be chosen to fit data). Sometimes, a free parameter is assigned a literature value measured in a different strain or even a different species. This is a poor practice for quantitative modeling, because literature values can vary by orders of magnitude [22]. Sometimes, a model is “calibrated” or “benchmarked” to fit experimental data [23], and thus free parameters become “fitting parameters.” This type of model calibration can also be problematic, because wrong models can also be calibrated to fit empirical data [23], and, not surprisingly, the resulting model predictions are likely wrong [23,24].

Even when all parameters are directly measured, quantitative modeling can still be challenging. First, a parameter measured from a cell population represents the population average and ignores cell-to-cell heterogeneity [25], which can be problematic. Second, parameter values may vary with the environment or time [26–29]. For example, the rate of acetate excretion by *Escherichia coli* is sensitive to the growth environment [28,29]. Third, during parameter measurements, cells may rapidly evolve, and thus parameters no longer correspond with the intended genotype. Fourth, in a model with multiple parameters, measurement uncertainty in each parameter can accumulate such that prediction confidence interval is too broad to be useful. Finally, the correctness or sufficiency of a particular model structure can be questionable. It is unclear how severe each of these problems can be in empirical examples, nor how to overcome these problems. As a result, it is not clear how feasible it is to perform quantitative modeling of living systems, including microbial communities.

**Fig 1:**
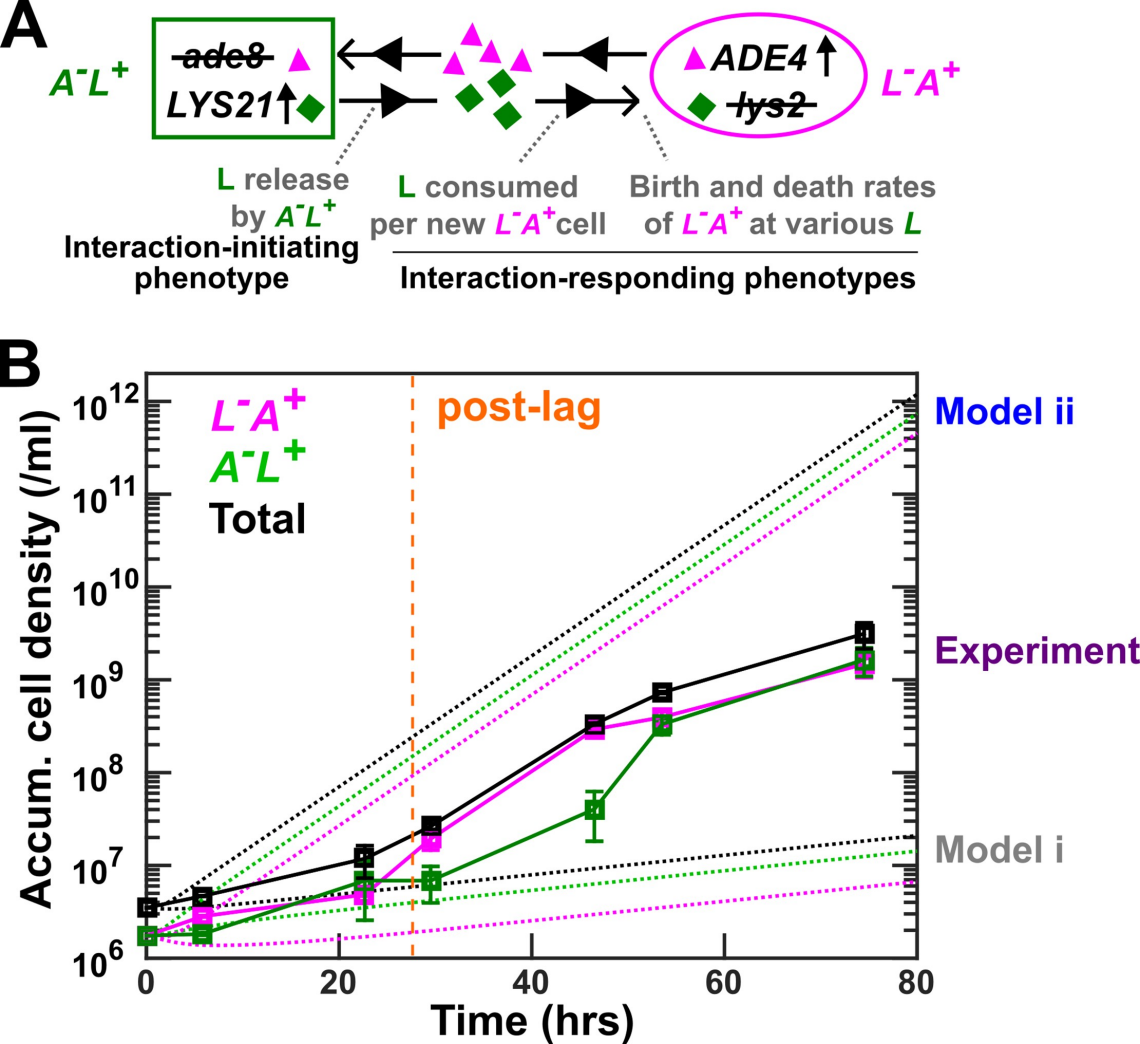
Model–experiment discrepancy in CoSMO growth dynamics. (**A**) CoSMO comprises two non-mating, cross-feeding yeast strains expressing different fluorescent proteins. Here, we use the lysine-mediated interaction as an example to illustrate interaction-initiating and interaction-responding phenotypes. (**B**) Model–experiment discrepancy. In experiments (squares; data listed in S1 Data), *L*^−^*A*^*+*^ and *A*^−^*L*^*+*^ growing exponentially in excess lysine or adenine were washed free of lysine or adenine and preconditioned (“Strain culturing and preconditioning” in Methods). They were mixed at 1:1 in SD at time zero to form CoSMO, which was then cultured in a well-mixed environment and diluted with SD as needed to ensure that no other nutrients were limiting. Population dynamics were tracked using flow cytometry (“Flow cytometry” in Methods), and dilutions were taken into account when calculating accumulative cell densities. CoSMO initially grew slowly (prior to orange dashed line), and then grew faster. We simulated CoSMO population dynamics (dotted lines; S1 Code) using Eqs 1–4, where parameters were measured in batch cultures (S2 Table). Models i and ii differed in that the release rates of the two strains were borrowed from our previous measurements in the S288C background (Model i) or directly measured in the RM11 background (Model ii). *ADE4* and *LYS21*: over-active mutant versions of the *ADE4* and *LYS21* genes, respectively; *ade8* and *lys2*: deletion mutations of the *ADE8* and *LYS2* genes, respectively; CoSMO, Cooperation that is Synthetic and Mutually Obligatory; L, lysine; SD, Synthetic Dextrose minimal medium.

Here, using a highly simplified community of engineered yeast cells, we stress test quantitative modeling of community dynamics. Our community “Cooperation that is Synthetic and Mutually Obligatory” (CoSMO) [17] consists of two differentially fluorescent, non-mating haploid *Saccharomyces cerevisiae* strains (Fig 1A; S1 Table). One strain, designated *A*^*−*^*L*^***+***^, cannot synthesize adenine (*A*) because of a deletion mutation in the *ADE8* gene, and over-activates the lysine (*L*) biosynthetic pathway due to a feedback-resistant *LYS21* mutation [30]. The other strain, designated *L*^*−*^*A*^***+***^, requires lysine because of a deletion mutation in the *LYS2* gene, and over-activates the adenine biosynthetic pathway due to a feedback-resistant *ADE4* mutation [31]. Overproduced metabolites in both strains are released into the environment and are consumed by the partner. In minimal medium lacking adenine and lysine supplements, the two strains engage in obligatory cooperation and stably coexist [17,32]. The biological relevance of CoSMO is as follows. First, simplified communities are useful for biotechnology applications [1,3,33]. For example, mutualistic communities similar to CoSMO have been engineered to divide up the labor of synthesizing complex drugs [34]. Second, cooperation and mutualisms modeled by CoSMO are widely observed in naturally occurring communities (including those in the gut and oral microbiota [35,36]) as microbes exchange essential metabolites such as amino acids and cofactors [37–42]. Indeed, principles learned from CoSMO, including how fitness effects of interactions affect the spatial patterning of community members, mechanisms that protect cooperators from non-cooperators, and how to achieve stable species composition in two-species communities, have been found to operate in communities of non-engineered microbes [32,43–45].

Because CoSMO has defined species interactions, and because all model parameters can be directly measured, we should be able to quantitatively predict community dynamics. Our initial model predictions significantly deviated from experimental measurements. In the process of resolving model–experiment discrepancies, we have uncovered and resolved multiple challenges in parameter quantification, a critical aspect of quantitative modeling. Because these challenges are likely general, our work serves as a road map that can be applied to quantitative modeling of other cell communities where interaction mechanisms can be inferred from genetic determinants (see Discussion).

## Results

### A model of community growth

Experimentally, CoSMO growth followed a reproducible pattern: after an initial lag marked by slow growth, the two populations and thus the entire community grew at a faster rate (Fig 1B, “Experiment”). Under optimized experimental conditions, post-lag growth rate reached a steady state (Fig 7A). We wanted to quantitatively predict CoSMO’s post-lag steady state growth rate (“growth rate”) *g*_*comm*_, the rate of total population increase. Community growth rate is a measure of how likely the community can survive periodic dilutions such as those in industrial fermenters [46] or during regular bowel movements. By “quantitative prediction,” we mean that model prediction should fall within experimental error bars.

We have formulated a differential equation model of the CoSMO dynamics as the following:

$$\frac{d\left[L^{-}A^{+}\right]}{dt}=\left(b_{L}\left(L\right)-d_{L}\right)\left[L^{-}A^{+}\right]$$ (1)

$$\frac{d\left[A^{-}L^{+}\right]}{dt}=\left(b_{A}\left(A\right)-d_{A}\right)\left[A^{-}L^{+}\right]$$ (2)

$$\frac{dL}{dt}=r_{L}\left[A^{-}L^{+}\right]-c_{L}b_{L}\left(L\right)\left[L^{-}A^{+}\right]$$ (3)

$$\frac{dA}{dt}=r_{A}\left[L^{-}A^{+}]-c_{A}b_{A}\left(A\right)[A^{-}L^{+}\right]$$ (4)

Eq 1 states that the *L*^*−*^*A*^***+***^ population density ([*L*^*−*^*A*^***+***^]) increases at a birth rate (*b*_*L*_) dependent on the concentration of lysine (*L*), and decreases at a fixed death rate (*d*_*L*_). Eq 2 describes how *A*^*−*^*L*^***+***^ population density ([*A*^*−*^*L*^***+***^]) changes over time. Eq 3 states that the concentration of lysine (*L*) increases due to releaser *A*^*−*^*L*^***+***^ releasing at a fixed rate (*r*_*L*_), and decreases as the *c*_*L*_ amount is consumed per birth of consumer *L*^*−*^*A*^***+***^. Eq 4 describes how the concentration *A* changes over time.

To predict community growth rate, we either simulated community dynamics (Fig 1B, dotted lines) or calculated it from an analytical formula (Eq 5) derived from Eqs 1–4 (see Methods, “Calculating steady state community growth rate”):

$$g_{comm}\approx -\frac{\left(d_{A}+d_{L}\right)}{2}+\sqrt{\frac{r_{A}r_{L}}{c_{A}c_{L}}}.$$ (5)

Eq 5 suggests that community growth rate depends on metabolite release rates (*r*_*A*_; *r*_*L*_) and metabolite consumption per cell birth (*c*_*A*_; *c*_*L*_) in a square root fashion, and depends on death rates (*d*_*A*_; *d*_*L*_) in a linear fashion. Simulations and analytical calculations yielded similar results (e.g., S1 Fig). Because death rates are small (Table 1) compared to community growth rate *g*_*comm*_ (0.11 ± 0.01/h in Fig 7B), release and consumption parameters are important and should be carefully measured. Eq 5 also states that even if one parameter is free, its value can always be chosen such that the calculated community growth rate will perfectly match experiments, regardless of the accuracy of the remaining five parameters. This is the well-known danger of free parameters.

**Table 1:** Experimentally measured strain phenotypes.

Strain	Phenotype	Symbol	Value	Lower	Upper	Unit	Reference	Exp#
1335	Max birth rate	*b*_*maxL*_	0.51	0.48	0.54	per h	[27]	4
1335	Lys for half max birth	*K*_*L*_	2.1	1.7	2.4	μM	[27]	4
1335	Birth cooperativity	*n*_*L*_	3.2	2.5	3.9		[27]	4
1335	Chemo death rate	*d*_*L*_	0.0024	0.0018	0.0030	per h	S7 Table	9
1335	Lys consumption	*c*_*L*_	5.4	5.1	5.7	fmole/cell	S5 Table	12
1335	Hyp release rate	*r*_*A*_	0.27	0.25	0.29	fmole/cell/h	S7 Table	24
1340	Max birth rate	*b*_*maxA*_	0.44	0.43	0.45	per h	[27]	4
1340	Hyp for half max birth	*K*_*A*_	1.3	1.2	1.4	μM	[27]	4
1340	Birth cooperativity	*n*_*A*_	1.5	1.4	1.7		[27]	4
1340	Chemo death rate	*d*_*A*_	0.015	0.014	0.016	per h	S8 Table	29
1340	Hyp consumption	*c*_*A*_	3.1	3.0	3.2	fmole/cell	S6 Table	32
1340	Lys release rate, starvation	*r*_*L*_(*H* = 0)	0.52	0.42	0.62	fmole/cell/h	S8 Table	7
1340	Lys release rate, T2 = 8 h	*r*_*L*_(*H* = 0.58)	0.83	0.70	0.96	fmole/cell/h	S8 Table	11
1340	Lys release rate, T2 = 7 h	*r*_*L*_(*H* = 0.65)	0.78	0.70	0.86	fmole/cell/h	S8 Table	6
1340	Lys release rate, T2 = 6 h	*r*_*L*_(*H* = 0.73)	0.53	0.45	0.61	fmole/cell/h	S8 Table	8
1340	Lys release rate, T2 = 5.4 h	*r*_*L*_(*H* = 0.80)	0.38	0.32	0.45	fmole/cell/h	S8 Table	4
1340	Lys release rate, T2 = 4 h	*r*_*L*_(*H* = 1.07)	0.08	0.05	0.11	fmole/cell/h	S8 Table	3

The upper and lower bounds of consumption amount per birth, release rate, and death rate in chemostats were calculated from the mean value plus and minus 2 SEMs, respectively. Birth rates *b* at various concentrations *s* of a limiting metabolite were measured using a high-throughput microscopy batch-culture assay and then fitted into the Moser growth model [47], ${b(s)=b}_{max}s^{n}/\left(s^{n}+K^{n}\right)$, where *b*_*max*_ is the maximal birth rate, *K* is the *s* at which *b*_*max*_/2 is achieved, and *n* is the growth cooperativity (akin to the Hill coefficient). The averages from four independent experiments were fitted into the Moser model to infer birth parameters (*b*_*max*_, *K*, and *n*) and their confidence intervals (for details, see [27]). Birth parameters were used in simulations but were not needed in Eq 5. *r*_*L*_(*H*) is the release rate of lysine at hypoxanthine concentration *H*, where *H* was inferred from the *A*^*−*^*L*^*+*^ growth model described above.

Abbreviations: Chemo, chemostat; Exp#, total number of experiments; Hyp, hypoxanthine; Lys, lysine; Max, maximal; SEM, standard error of the mean; T2, doubling time.

Model parameters correspond to strain phenotypes and include metabolite release rate, metabolite consumption per birth, and cell birth and death rates. Even though these phenotypes reflect strain interactions (“interaction phenotypes” in Fig 1A), we measured them in monocultures to eliminate partner feedback. In our earlier studies, we quantified some of these phenotypes and borrowed others from literature values [17,32,44]. Our models correctly predicted various properties of CoSMO, including the steady-state strain ratio [17] as well as qualitative features of spatial patterning [32,44].

### Initial models based on batch-culture parameters poorly predict community growth rate

Our first model (Model i) underestimated community growth rate. Unlike the published strains of *A*^*−*^*L*^***+***^ and *L*^*−*^*A*^***+***^ in the S288C background [17], strains in this study were constructed in the RM11 background to reduce mitochondrial mutation rate [48]. For each RM11 strain, we measured death rate during starvation using a microscopy batch-culture assay [27]. We also quantified the amount of metabolite consumed per birth in batch cultures grown to saturation (see Fig 4B for details; S13 Fig), similar to our earlier work [17]. Because release rates were more tedious to measure, we initially borrowed published release rates of *L*^*−*^*A*^***+***^ and *A*^*−*^*L*^***+***^ in the S288C background in batch starved cultures [17]. Predicted community growth rates were much slower than experimental measurements (Fig 1B, “Model i”; Fig 7B, gray).

A revised model (Model ii) without any borrowed parameters overestimated community growth rate. For this model, we directly measured the release rates of RM11 *L*^*−*^*A*^***+***^ and *A*^*−*^*L*^***+***^ in batch starved cultures (see Fig 5B and S15B Fig for details). The release rates of both strains in the RM11 background were approximately 3-fold higher than those in the S288C background (S2 Table). Consequently, the predicted community growth rate greatly exceeded experiments (Fig 1B, “Model ii”; Fig 7B, blue).

### Identifying interaction mediators

**Fig 2:**
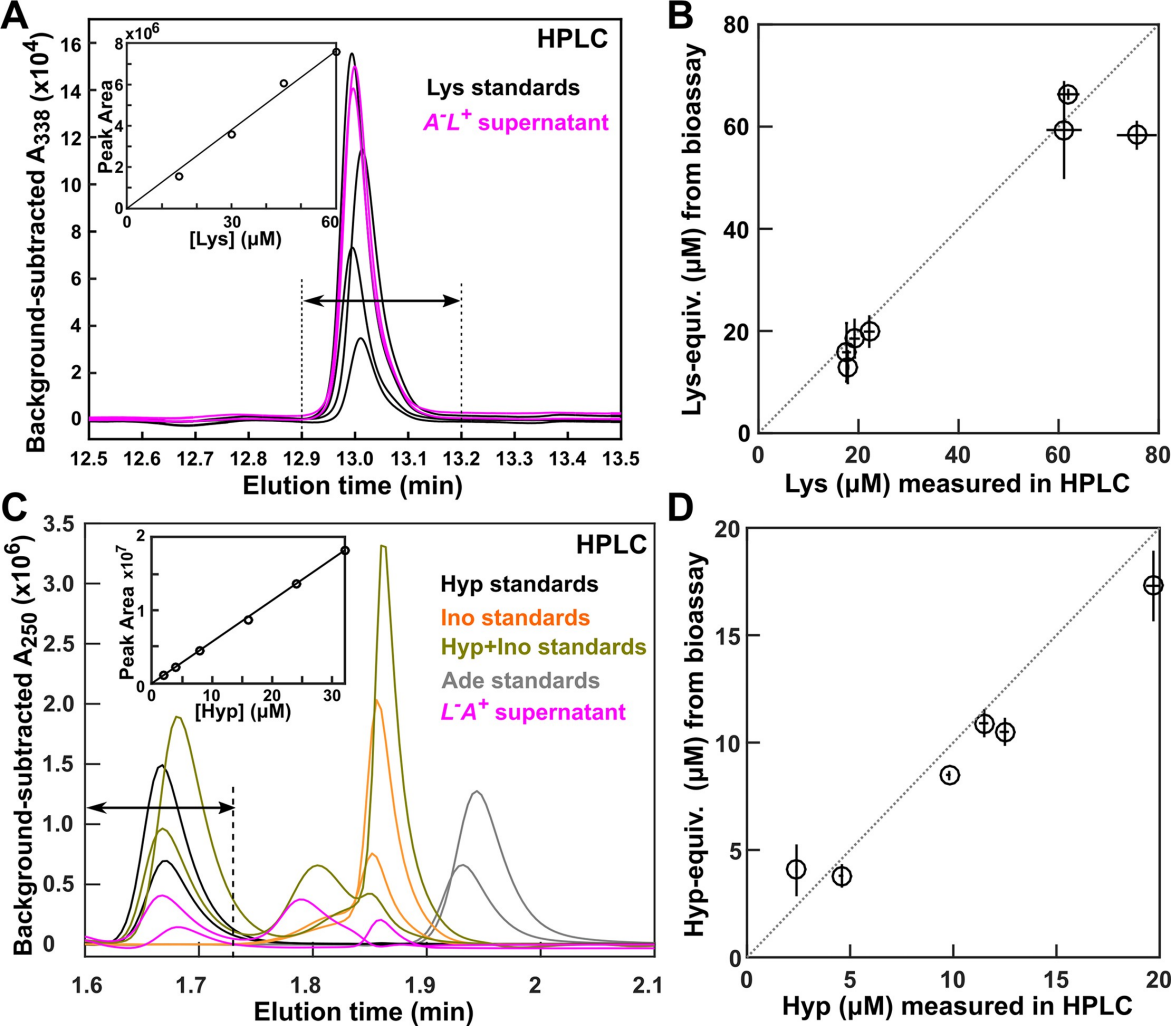
Lysine and hypoxanthine mediate interactions in CoSMO. (**A, B**) Lysine mediates the interaction from *A*^*−*^*L*^***+***^ to *L*^*−*^*A*^***+***^. (**A**) *A*^*−*^*L*^***+***^ releases lysine. Supernatants of *A*^*−*^*L*^***+***^ (magenta) as well as SD supplemented with various concentrations of lysine standards (black) were derivatized and run on HPLC (Methods, “HPLC”). Inset: standard curve on which the peak areas between 12.9 min and 13.2 min (arrows) were plotted against lysine concentrations. (**B**) In *A*^*−*^*L*^***+***^ supernatants, lysine concentrations quantified by HPLC agreed with concentrations of lysine equivalents that supported *L*^*−*^*A*^***+***^ in a yield bioassay (Methods, “Bioassays”). Circles indicate average values, and bars represent the spread of two measurements. (**C, D**) Hypoxanthine mediates the interaction from *L*^*−*^*A*^***+***^ to *A*^*−*^*L*^***+***^. (**C**) *L*^*−*^*A*^***+***^ releases hypoxanthine and inosine. HPLC traces of *L*^*−*^*A*^***+***^ supernatants (magenta) and standards of SD supplemented with two different concentrations of hypoxanthine (black), inosine (orange), adenine (gray), or a mixture of hypoxanthine and inosine (olive). Inset: standard curve on which the peak areas in the window between 1.6 min and 1.732 min (arrows) were plotted against hypoxanthine concentrations and used to quantify hypoxanthine. Because the HPLC elution profile could vary between independent runs (e.g., compare the two olive curves), quantification windows were adjusted accordingly. (**D**) In *L*^*−*^*A*^***+***^ supernatants, hypoxanthine concentrations quantified by HPLC agreed with concentrations of purines that supported *A*^*−*^*L*^***+***^ growth as quantified by the yield bioassay. In **B** and **D**, dotted lines have a slope of 1. All data can be found in S2 Data. Ade, adenine; CoSMO, Cooperation that is Synthetic and Mutually Obligatory; HPLC, high-pressure liquid chromatography; Hyp, hypoxanthine; Ino, inosine; Lys, lysine; SD, Synthetic Dextrose minimal medium.

One possible cause for the model–experiment discrepancy could be that cells engineered to overproduce adenine or lysine [30,31] might instead release derivatives of adenine or lysine. Consequently, when we quantified phenotypes such as metabolite consumption, we could have supplemented the wrong metabolite and been misled. A genome-scale metabolic model of *S*. *cerevisiae* predicted that although *A*^*−*^*L*^***+***^ likely released lysine, *L*^*−*^*A*^***+***^ likely released hypoxanthine or adenosine-(3,5)-biphosphate instead of adenine [49,50]. Nanospray desorption electrospray ionization mass spectrometry imaging (nanoDESI MS) [51] performed by the Julia Laskin lab revealed a lysine gradient emanating from *A*^*−*^*L*^***+***^ and hypoxanthine and inosine gradients emanating from *L*^*−*^*A*^***+***^, although the signals were noisy. We followed up this observation using high-pressure liquid chromatography (HPLC) (Fig 2).

Indeed, lysine mediates the interaction from *A*^*−*^*L*^***+***^ to *L*^*−*^*A*^***+***^. We subjected *A*^*−*^*L*^***+***^ supernatant to HPLC (Methods, “HPLC”) and a yield-based bioassay (Methods, “Bioassays”). In HPLC, a compound in *A*^*−*^*L*^***+***^ supernatant eluted at the same time as the lysine standards (Fig 2A), and its concentration could be quantified by comparing the peak area against those of lysine standards (Fig 2A inset). In bioassay, we quantified the total lysine-equivalent compounds in an *A*^*−*^*L*^***+***^ supernatant by growing *L*^*−*^*A*^***+***^ in it and comparing the final turbidity with turbidities achieved in minimal medium supplemented with various known concentrations of lysine. HPLC quantification agreed with the yield bioassay (Fig 2B). Thus, lysine-equivalent compounds released by *A*^*−*^*L*^***+***^ were primarily lysine.

Hypoxanthine mediates the interaction from *L*^*−*^*A*^***+***^ to *A*^*−*^*L*^***+***^. When we subjected *L*^*−*^*A*^***+***^ supernatants to HPLC, we found compounds at the elution times of hypoxanthine and inosine, but not of adenine (Fig 2C). Hypoxanthine but not inosine supported *A*^*−*^*L*^***+***^ growth, and inosine did not affect how hypoxanthine stimulated *A*^*−*^*L*^***+***^ growth (S3 Fig). Hypoxanthine concentration quantified by HPLC agreed with the concentration of purines consumable by *A*^*−*^*L*^***+***^ in the yield bioassay (Fig 2D; Methods, “Bioassays”). Thus, *A*^*−*^*L*^***+***^ primarily consumed hypoxanthine released by *L*^*−*^*A*^***+***^.

Using phenotypes of *A*^*−*^*L*^***+***^ measured in hypoxanthine versus adenine happened to not affect model performance. Death and release rates were not affected because they were measured in the absence of purine supplements. Similar amounts of hypoxanthine and adenine were consumed to produce a new *A*^*−*^*L*^***+***^ cell (S3 Fig). Although the birth rate of *A*^*−*^*L*^***+***^ was slower in the presence of hypoxanthine compared with adenine, especially at low concentrations (S4 Fig), this difference did not affect community growth rate (Eq 5). Thus, distinguishing whether hypoxanthine or adenine was the interaction mediator did not make a difference in predicting community growth rate (S1 Fig). Here, we continue to use *A* to represent the adenine precursor hypoxanthine.

### Rapid evolution during chemostat measurements of strain phenotypes

**Fig 3:**
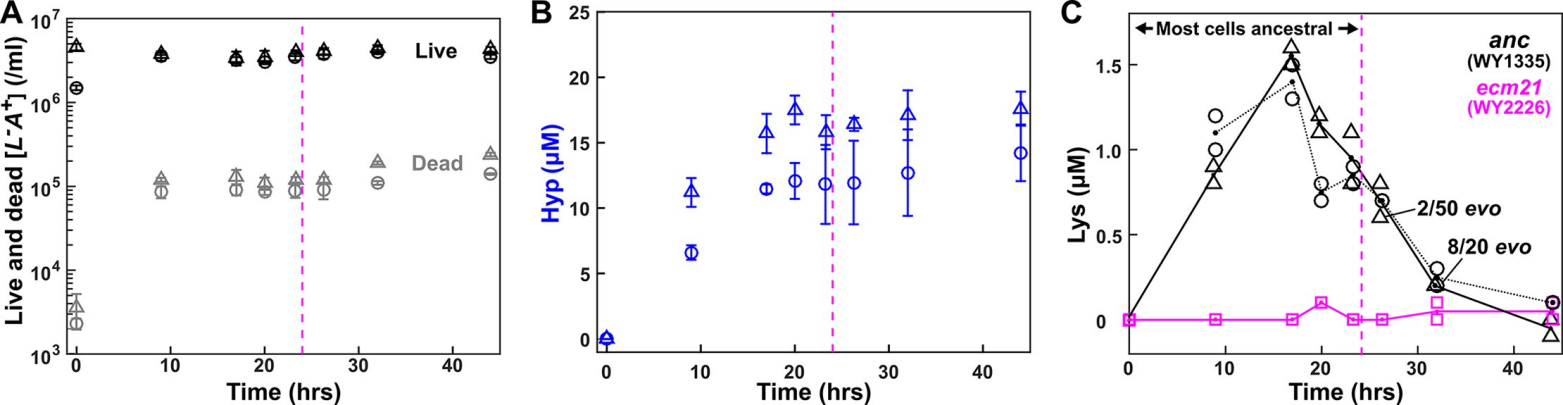
Chemostat dynamics reveals rapid evolution. *L*^*−*^*A*^***+***^ (WY1335) cells growing exponentially in excess lysine were washed free of lysine and inoculated into the culturing vessel (triangles: inoculation at near steady-state density in the absence of lysine; circles: inoculation at 1/3 steady-state density in the presence of 5–10 μM lysine). Minimal medium containing 20 μM lysine was dripped into the culturing vessel (19 mL) to achieve an 8-h doubling time (19 mL * ln(2)/8 h = 1.646 mL/h; “Chemostat culturing” in Methods). (**A**) Live and dead cell densities (Methods, “Flow cytometry”) and (**B**) released hypoxanthine (Methods, “Bioassays”) reached steady state by about 10 h and about 20 h, respectively. Error bars represent 2 standard deviations. (**C**) Lysine concentrations in culturing vessels failed to maintain a steady state due to rapid evolution (Methods, “Detecting evolved clones”). Instead, lysine concentrations rapidly declined to a level similar to that in a chemostat inoculated with an *L*^*−*^*A*^***+***^ mutant with improved affinity for lysine [43,53] (“*ecm21*,” magenta). Indeed, when we tested chemostat samples (triangles), 8 out of 20 tested clones were evolved by 32 h (about four generations). For each sample, two measurements of lysine concentrations and their average were plotted. Magenta dashed lines mark the time before which >90% of population remained ancestral. All plotted data are in S3 Data. *anc*, ancestor; *evo*, evolved; *ecm21*, a strain harboring a deletion mutation of the *ECM21* gene; Hyp, hypoxanthine; Lys, lysine.

Model–experiment discrepancy (Fig 1B) could be caused by phenotypes being dependent on the environment. So far, we had measured phenotypes in batch cultures containing zero or excess metabolite. Thus, we set out to remeasure strain phenotypes in chemostats [52] that mimicked CoSMO environments. Specifically, in a chemostat, fresh medium containing the required metabolite (lysine or hypoxanthine) was pumped into the culturing vessel at a fixed rate (“dilution rate”), while culture medium containing cells exited the culturing vessel at the same rate (Methods, “Chemostat culturing”). After an initial adjustment stage, live population density reached a steady state (Fig 3A), which meant that the population grew at the same rate as the dilution rate (Eqs 6–11 in Methods, “Quantifying phenotypes in chemostats”) [52]. By setting the chemostat dilution rate to various growth rates experienced by CoSMO (i.e., 5.5-h to 8-h doubling), we could mimick the CoSMO growth environments. From the population and chemical dynamics in the chemostat, we could then measure metabolite release rate, metabolite consumption per birth, and death rate (Eqs 12–16 in Methods, “Quantifying phenotypes in chemostats”).

During chemostat measurements, ancestral *L*^*−*^*A*^***+***^ was rapidly overtaken by mutants with dramatically improved affinity for lysine (Fig 3C; S7 Fig; Methods, “Detecting evolved clones”), consistent with our earlier work [43]. These mutants, likely being present in the inoculum at a low (on the order of 10^*−*6^) frequency, displayed a growth rate 3.6-fold that of the ancestor during lysine limitation (S7 Fig). Thus, to measure ancestral *L*^*−*^*A*^***+***^ phenotypes, we terminated measurements before mutants could take over (<10%, before magenta dashed lines in Fig 3).

In contrast, the evolutionary effects of *A*^*−*^*L*^***+***^ mutants on CoSMO growth were captured during phenotype measurements. Unlike *L*^*−*^*A*^***+***^ mutants, *A*^*−*^*L*^***+***^ mutants were constantly generated from ancestral cells at an extremely high rate (on the order of 0.01/cell/generation; Methods, “Evolutionary dynamics of mutant *A*^*−*^*L*^*+*^”), presumably via frequent chromosome duplication (S8C Fig). These mutants were present at a significant frequency (1%–10%), even before our measurements started, and slowly rose to 30%–40% during measurements due to their moderate fitness advantage over the ancestor under hypoxanthine limitation (S8A Fig; S9 Fig; Methods, “Detecting evolved clones”). Consequently, we measured the average phenotypes of an evolving mixture of ancestors and mutants. Fortunately, these averaged phenotypes could be used to model CoSMO, because mutants accumulated in similar fashions during phenotype measurements and during CoSMO measurements so long as the two time windows were compatible (S9B Fig; S11 Fig).

### Metabolite consumption is sensitive to the environment

**Fig 4:**
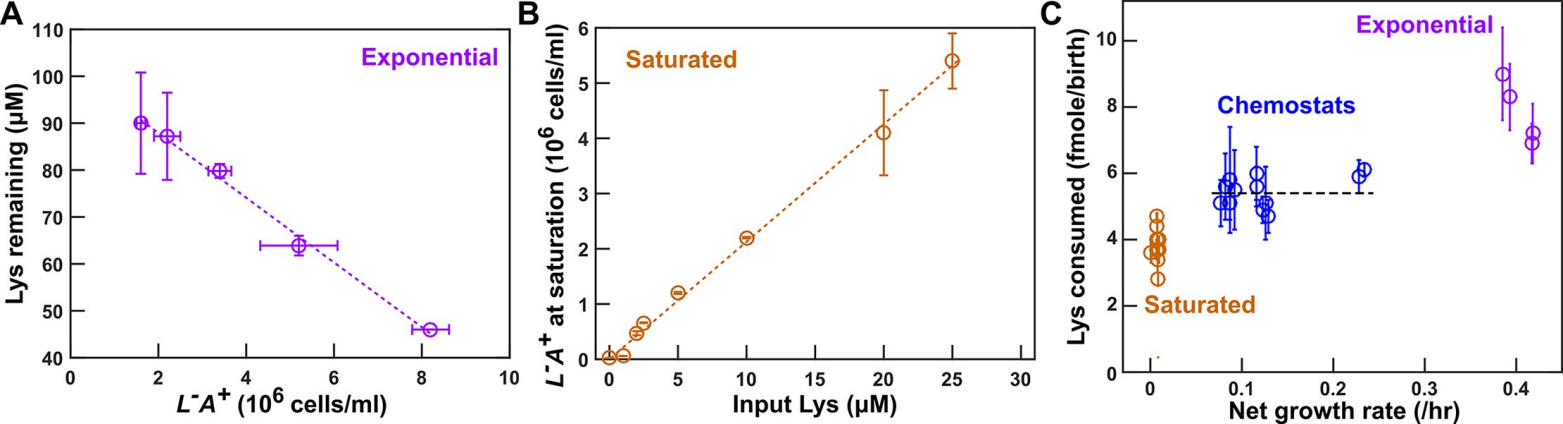
Metabolite consumption is sensitive to the environment. (**A**) Consumption in excess lysine. *L*^*−*^*A*^***+***^ population density and lysine concentration remaining in the medium were measured over time (Methods, “Measuring consumption in batch cultures”). Consumption per birth was calculated from the slope of the lavender line. (**B**) Consumption in cultures grown to saturation. *L*^*−*^*A*^***+***^ cells were inoculated into SD supplemented with various concentrations of lysine. After cultures had reached saturation, total cell densities were measured by flow cytometry. Lysine consumed per birth was quantified from 1/(slope of the orange line). This value was used in Models i and ii. (**C**) Consumption per birth in different environments. Lysine consumption was measured in lysine-limited chemostats at various doubling times (Methods, “Quantifying phenotypes in chemostats,” and Eq 12), and data were jittered slightly along the *x* axis to facilitate visualization. For chemostat measurements, error bars represent 2 standard deviations caused by fluctuations in steady-state population density. For exponential and saturation consumption, error bars mark 2 SEMs for slope estimation. The black dashed line marks the average lysine consumption per *L*^*−*^*A*^***+***^ birth in chemostats (Table 1; S5 Table), which we used in Model iii. All plotted data can be found in S4 Data. Lys, lysine; SD, Synthetic Dextrose minimal medium; SEM, standard error of the mean.

Metabolite consumption per birth depends on the growth environment. Consistent with our previous work [17], consumption during exponential growth in excess supplement was higher than that in a culture grown to saturation (Fig 4; Methods, “Measuring consumption in batch cultures”), presumably due to exponential phase cells storing excess metabolites [54]. Consumption in chemostats (Methods, “Quantifying phenotypes in chemostats,” Eq 12) was in between exponential and saturation consumption (Fig 4C for *L*^*−*^*A*^***+***^ and S13 Fig for *A*^*−*^*L*^***+***^). For both strains, because consumption in chemostat was relatively constant across the range of doubling times encountered in CoSMO (5.5–8 h), we used the average value in Model iii (dashed line in Fig 4C and S13 Fig; Table 1; S5 Table, S6 Table).

### Live *L*^*−*^*A*^*+*^ releases hypoxanthine upon lysine limitation

Metabolites can be released by live cells or leaked from dead cells. We want to distinguish between live versus dead release for the following reasons. First, if death rate were to evolve to be slower, then live release would predict increased metabolite supply, whereas dead release would predict the opposite. Second, dead release would imply nonspecific release and, thus cell–cell interactions may be highly complex. Finally, leakage from dead cells is thermodynamically inevitable, whereas active release of costly molecules would require an evolutionary explanation.

**Fig 5:**
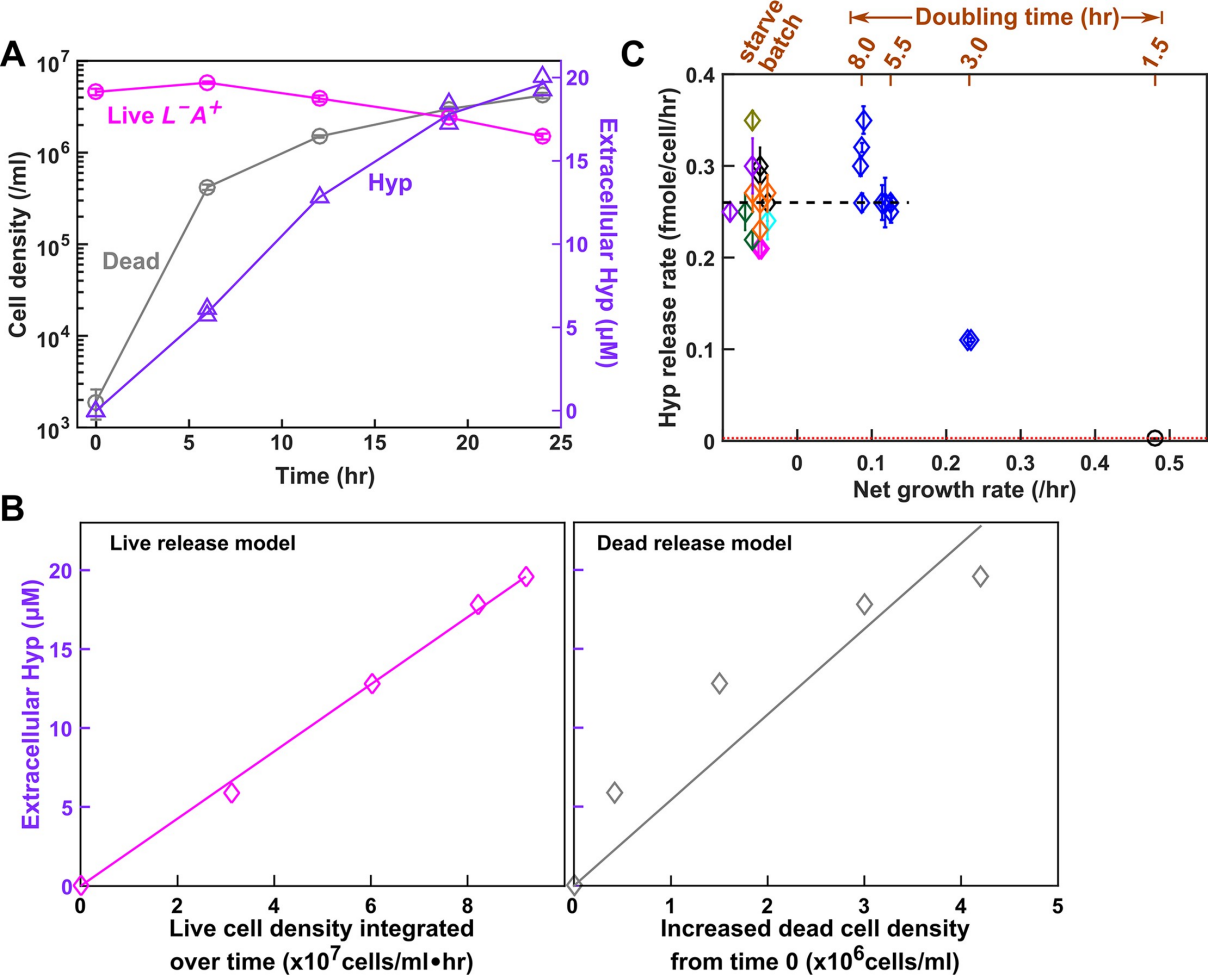
Hypoxanthine is released by live *L*^*−*^*A*^*+*^ upon limitation for lysine. (**A**) Hypoxanthine release during lysine starvation. Exponential *L*^*−*^*A*^***+***^ cells were washed free of lysine and diluted into SD. Live and dead population densities and supernatant hypoxanthine concentrations were measured over time (Methods, “Flow cytometry”; the yield bioassay in “Bioassays”). (**B**) The “live release” model fits data better than the “dead release” model. (Left) If hypoxanthine was released by live cells at a constant rate, then hypoxanthine concentration should scale linearly against live cell density integrated over time. (Right) If hypoxanthine was released upon cell death, then hypoxanthine concentration should scale linearly against dead cell density. The live release model has better linearity than the dead release model, and therefore hypoxanthine is likely released by live cells. The release rate measured in the left panel of **B** was used in Models i and ii. Data for A and B can be found in S5 Data. **(C)** Hypoxanthine release rate as a function of growth rate. Hypoxanthine release rates were plotted for *L*^*−*^*A*^***+***^ in the presence of excess lysine (black circle on the right), in lysine-limited chemostats with doubling times ranging from 3 to 8 h (blue; S14 Fig blue), and during lysine starvation (plotted at <0 net growth rate, with different colors indicating experiments on different days). The black dashed line marks the average release rate measured from starvation up to 5.5-h chemostats (Table 1), which was used in Model iii. Hypoxanthine release rate in excess lysine was lower than the detection limit (i.e., <0.003 fmole/cell/h, marked by the red dotted line; Methods, “Measuring the upper bound of release rate in excess metabolites”). Release rates of *L*^*−*^*A*^***+***^ are in S7 Table. Hyp, hypoxanthine; SD, Synthetic Dextrose minimal medium.

Hypoxanthine is likely released by live *L*^*−*^*A*^***+***^. In the absence of lysine (Methods, “Starvation release assay”), we tracked the dynamics of live and dead *L*^*−*^*A*^***+***^ (Fig 5A, magenta and gray) and of hypoxanthine accumulation (Fig 5A, lavender). If live cells released hypoxanthine, then hypoxanthine should increase linearly with live cell density integrated over time (i.e., the sum of live cell density * h, Fig 5B, left), and the slope would represent the live release rate (fmole/cell/h). If cells released hypoxanthine upon death, then hypoxanthine should increase linearly with dead cell density, and the slope would represent the amount of metabolite released per cell death (Fig 5B, right). Because the live release model explained our data better than the dead release model (Fig 5B), hypoxanthine was likely released by live cells during starvation. In lysine-limited chemostats, we could not use dynamics to distinguish live from dead release (note the mathematical equivalence between Eqs 9 and 10 in Methods, “Quantifying phenotypes in chemostats”). Instead, we harvested cells and chemically extracted intracellular metabolites (Methods, “Extraction of intracellular metabolites”). Each *L*^*−*^*A*^***+***^ cell, on average, contained 0.12 (±0.02, 95% CI) fmole of hypoxanthine (Methods, “HPLC”). If hypoxanthine was released by dead cells (about 10^5^ dead cells/mL, Fig 3A), we should see 0.012 μM instead of the observed approximately 10 μM hypoxanthine in the supernatant (Fig 3B). Thus, hypoxanthine is likely released by live *L*^*−*^*A*^***+***^ in chemostats.

Hypoxanthine release rates of *L*^*−*^*A*^***+***^ are similar in lysine-limited chemostats mimicking the CoSMO environments (Methods, “Quantifying phenotypes in chemostats,” Eq 14) versus during starvation (Fig 5C). Thus, we used the average hypoxanthine release rate (Fig 5C black dashed line; Table 1) in Model iii. Note that release rates declined in faster-growing cultures (≤3-h doubling; Fig 5C), but we did not use these data because CoSMO did not grow that fast.

### *A*^*−*^*L*^*+*^ intracellular lysine content and lysine release rate vary with the environment

Lysine is likely released by live *A*^*−*^*L*^***+***^. When we measured lysine release from starving *A*^*−*^*L*^***+***^ cells (S15A Fig), a model assuming live release and a model assuming dead release generated similar matches to experimental dynamics (S15B and S15C Fig). However, after measuring intracellular lysine content, we concluded that dead release was unlikely, because each dead cell would need to release significantly more lysine than that measured inside a cell to account for supernatant lysine concentration, especially during the early stage of starvation (S16B Fig).

**Fig 6:**
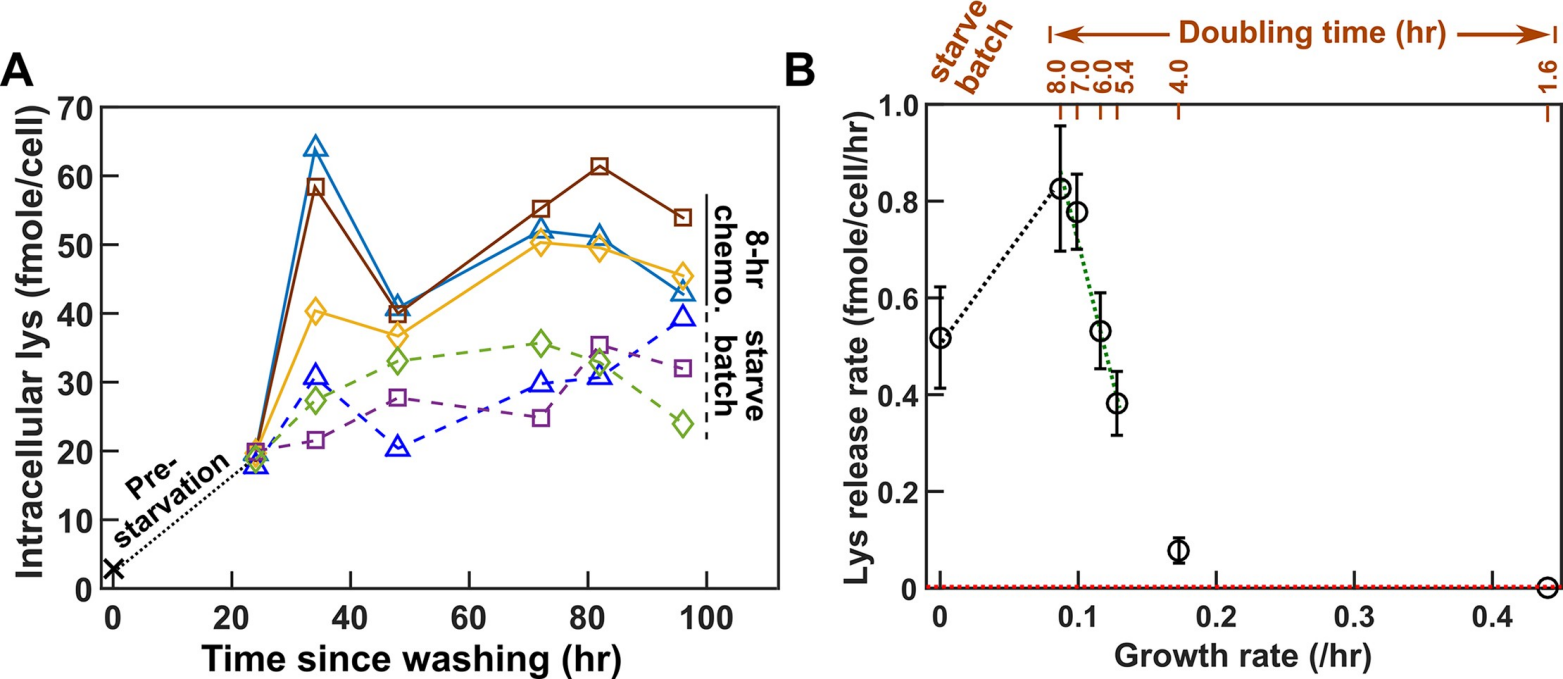
Intracellular lysine content and lysine release rate of *A*^*−*^*L*^*+*^ vary with environment. **(A)** Intracellular lysine content increases upon hypoxanthine limitation. *A*^*−*^*L*^***+***^ cells grown to exponential phase in SD plus excess hypoxanthine were washed and diluted into SD at time zero. Cells were either starved further (“starve batch,” dashed lines) or inoculated into hypoxanthine-limited chemostats after 24 h of prestarvation (“8-h chemo.,” solid lines). At various times, cells were harvested, and intracellular lysine was extracted (Methods, “Extraction of intracellular metabolites”) and measured via yield bioassay (Methods, “Bioassays”). Intracellular lysine content increased by 6-fold during the 24-h prestarvation, even though the average cell size increased by only about 20% (S17 Fig). Intracellular lysine content continued to increase, reaching a higher level in 8-h chemostats than in starvation. Different colors represent different experiments. Data can be found in S6 Data. (**B**) Lysine release rate varies with the environment. Lysine release rates were quantified for cells at different growth rates (doubling times marked above) and during starvation (e.g., S15 Fig). The time window for phenotype measurement is similar to that for CoSMO growth rate measurement to ensure similar evolutionary effects (S9 Fig). Mean release rates and their 2 SEMs were plotted. Lysine release rate in an exponential batch culture in excess hypoxanthine was below the level of detection (red dotted line = 0.003 fmole/cell/h; Methods, “Measuring the upper bound of release rate in excess metabolites”). The green dotted regression line in **B** was used in analytical calculation (Eq 5; Methods, “Calculating steady-state community growth rate”), while the black and the green dotted regression lines in **B** were used in spatial simulations. Lysine release rates were summarized in S8 Table and plotted in greater detail in S20 Fig. chemo., chemostat; CoSMO, Cooperation that is Synthetic and Mutually Obligatory; Lys, lysine; SD, Synthetic Dextrose minimal medium; SEM, standard error of the mean.

Lysine release rate of *A*^*−*^*L*^***+***^ is highly sensitive to the growth environment (Fig 6B, details in S20 Fig) and reaches a maximum at an intermediate growth rate. Release rates in 7–8-h doubling chemostats were about 60% more than those during starvation. Lysine release rate rapidly declined as hypoxanthine became more available (i.e., as growth rate increased, Fig 6B). Variable release rate could be due to variable intracellular lysine content: lysine content per cell increased by severalfold upon removal of hypoxanthine (from 2.9 fmole/cell to about 19 fmole/cell; Fig 6A black dotted line) and leveled off at a higher level in 8-h chemostats than during starvation (Fig 6A). We incorporated a variable lysine release rate in Model iii (Table 1).

### Parameters measured in community-like environments enable prediction of CoSMO growth rate

Death rates, which could affect CoSMO growth rate (Methods, Eq 5), are also sensitive to the environment. We measured death rates in chemostats (Methods, “Quantifying phenotypes in chemostats,” Eq 13 or Eq 16) and found them to be distinct from the death rates in zero or excess metabolite (S21 Fig). Because death rates were relatively constant in chemostats mimicking the CoSMO environments (S21 Fig, blue lines), we used the averaged values in Model iii (Table 1; S7 Table; S8 Table).

Our chemostat-measured model parameters are internally consistent: mathematical models of *L*^*−*^*A*^***+***^ in lysine-limited chemostat (S4 Code) and of *A*^*−*^*L*^***+***^ in hypoxanthine-limited chemostat (S5 Code) captured experimental observations (S12 Fig; S19 Fig).

**Fig 7:**
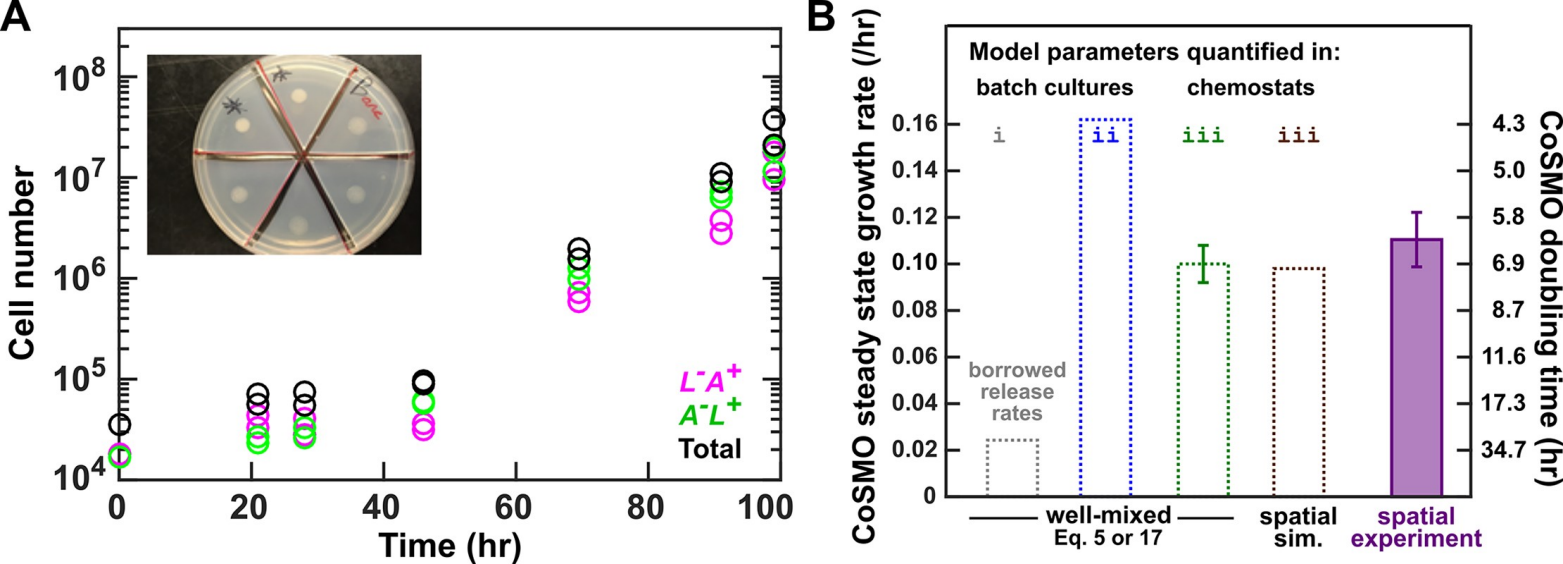
Steady-state CoSMO growth rate can be predicted from parameters measured in chemostats. (**A**) Steady-state CoSMO growth rate on agarose pad. Preconditioned *L*^*−*^*A*^***+***^ and *A*^*−*^*L*^***+***^ were mixed at approximately 1:1 ratio, and 15 μL of 4 × 10^4^ total cells was spotted on the center of agarose pads (inset), forming an inoculum spot of radius 4 mm (S24A Fig; Methods, “Quantifying spatial CoSMO growth dynamics”). Periodically, cells from pads were washed off into water and subjected to flow cytometry. (**B**) A comparison of model predictions and experiments. Steady-state growth rate of well-mixed CoSMO was calculated from Eq 5 or Eq 17 (with nearly identical results) when we borrowed release rates from a different strain background in starved batch culture (gray; Model i in Fig 1B), when all parameters were measured from the correct strain background in batch cultures (blue; Model ii in Fig 1B), and when all parameters were measured from the correct strain background in chemostats (green; Model iii). A partial differential equation model (i.e., the spatial version of Model iii) was simulated for spatial CoSMO under various experimental configurations, yielding similar predictions on spatial CoSMO growth rate (S23 Fig; S6 Code; S7 Code). The average value was plotted here (brown). In simulations, CoSMO grew at a similar rate in a spatially structured environment as in a well-mixed environment. This is because in spatial CoSMO, concentrations of metabolites eventually became uniform in the agarose and in the community (S25 Fig) and because the two strains formed small (tens of micrometers) patches that were intermixed [32]. The green error bar (95% confidence interval) was calculated from uncertainties in parameter estimations (i.e., 2 SEMs in Table 1) via the method of error propagation (Methods, “Calculating steady-state community growth rate”). Under various experimental configurations (Methods, “Quantifying spatial CoSMO growth dynamics”), steady-state CoSMO growth rates were similar, and the average value and 2 standard deviations from 11 independent experiments were plotted (purple; S4 Table). All data can be found in S7 Data. CoSMO, Cooperation that is Synthetic and Mutually Obligatory; SEM, standard error of the mean; sim., simulation.

Using parameters measured in chemostats (Table 1), model prediction on CoSMO growth rate quantitatively matches experimental results. Experimentally, because *L*^*−*^*A*^***+***^ mutants quickly took over well-mixed CoSMO (red in S22A Fig [43]), we grew CoSMO in a spatially-structured environment so that fast-growing mutants were spatially confined to their original locations and remained a minority (red in S22B Fig). Spatial CoSMO growth rates measured under a variety of experimental setups (e.g., agarose geometry and initial total cell density) remained consistent (0.11 ± 0.01/h; Fig 7B purple; S24 Fig). In Model iii, an analytical formula (Eq 5; Methods, “Calculating steady-state community growth rate”) and spatial CoSMO simulations based on chemostat-measured parameters (Table 1) both predicted CoSMO growth rate to be 0.10 ± 0.01/h (Fig 7B, green and brown). Thus, chemostat parameters allowed our model to quantitatively explain experimental CoSMO growth rate (Fig 7 green and brown versus purple). This also suggests that our parameter measurements are valid. Note that although Model iii captures the steady-state growth rate of CoSMO, it fails to recapitulate quantitative details of strain dynamics during and immediately after the initial lag phase (S26 Fig). This is not surprising, because strain phenotypes during starvation are complex (e.g., being time dependent, S18B Fig) [27] and differ from those in chemostats (Fig 4; Fig 6; S13 Fig; S21 Fig).

In summary, phenotypic parameters are often sensitive to the environment. Thus, measuring phenotypes in a range of community-like environments may be required for quantitative modeling. Rapid evolution may further interfere with parameter measurements and model testing. Only after overcoming these challenges did we succeed in quantitatively predicting the steady-state growth rate of CoSMO.

## Discussion

Microbial communities are complex. Thus, qualitative modeling has been deployed to yield biological insights [55,56]. However, one would eventually like to understand how community-level properties quantitatively emerge from interactions among member species. The simplicity of CoSMO has allowed us to directly measure all parameters, uncover some of the challenges to quantitative modeling, and devise means to overcome these challenges. These challenges are likely general to other living systems. Below, we discuss what we have learned from quantitative modeling of CoSMO steady-state growth rate.

Even when genetic determinants are known, interaction mediators can be nontrivial to identify. In CoSMO, we previously thought that adenine was released by *L*^*−*^*A*^***+***^, whereas in reality, hypoxanthine and inosine are released (Fig 2). Fortuitously, hypoxanthine but not inosine affects *A*^*−*^*L*^***+***^ growth (S3 Fig). Otherwise, we might be forced to quantify how hypoxanthine and inosine, in isolation and in different combinations, might affect *A*^*−*^*L*^***+***^. *A*^*−*^*L*^***+***^ grows faster in adenine than in hypoxanthine (S4 Fig), and although this does not affect our prediction of CoSMO growth rate (S1 Fig), it could affect predictions on other community properties.

Many mathematical models have relied on free parameters, which can be problematic when predictions are sensitive to the values of free parameters. In the case of CoSMO, release rates from two strain backgrounds differed by severalfold (S2 Table), and not surprisingly, borrowing parameters affected prediction (Fig 1B).

A major challenge we uncovered was environment-sensitive parameters. A key assumption in modeling is invariant parameters. As we have demonstrated here, phenotypes (e.g., metabolite consumption per birth, metabolite release rate, and death rate) measured in zero or excess metabolite can differ dramatically from those measured in metabolite-limited chemostats (Fig 4**C**; Fig 5C; Fig 6B; S13 Fig; S21 Fig). Furthermore, even within the range of metabolite limitation experienced by CoSMO (doubling times of 5.4–8 h), lysine release rate varied by as much as 2-fold (Fig 6B), which could be caused by variable intracellular metabolite concentrations (Fig 6A). Based on parameters measured in chemostats (including variable lysine release rate), Model iii quantitatively predicts experimental results (Fig 7). Environment-sensitive parameters make quantitative modeling intrinsically difficult, because community environment often changes with time, and so will environment-sensitive model parameters. Even if we are only interested in predicting the steady-state community property, we may not know in advance what that steady state is and thus which environment to measure parameters in. For complex communities, multiple states could exist [57]. Thus, we may need to measure parameters in a range of environments that are typically encountered in a community.

Another obstacle for model building and testing is rapid evolution. If we quantify phenotypes in starved batch cultures, cells do not grow and thus evolution is slow, but the environment deviates significantly from the community environment. In chemostat measurements, we can control the environment to mimic those encountered by the community. However, in addition to the time-consuming nature of constructing and calibrating chemostats to ensure accurate flow rates [58], rapid evolution occurs. For *L*^*−*^*A*^***+***^, mutants pre-exist at a low frequency but can grow severalfold faster than the ancestor (S7 Fig). Consequently, a population will remain largely (>90%) ancestral only for the first 24 h in the well-mixed chemostat environment (Fig 3). A short measurement time window poses additional challenges if, for example, the released metabolite has not accumulated to a quantifiable level. For *A*^*−*^*L*^***+***^, mutants are generated from ancestral cells at an extremely high rate before and during phenotype quantification (Methods, “Evolutionary dynamics of mutant *A*^*−*^*L*^*+*^”; S9 Fig). Because mutants accumulated at a similar rate in CoSMO (S9B Fig), we accounted for evolutionary effects by using similar quantification time windows for *A*^*−*^*L*^***+***^ phenotypes and for CoSMO growth rate. Note that this approximation is valid here, because our model (without any free parameters) matches experiments quantitatively (Fig 7B). Rapid evolution also poses a problem for model testing. For example, when quantifying CoSMO growth rate, which requires several days, we were forced to use a spatially structured environment so that fast-growing *L*^*−*^*A*^***+***^ mutants could not take over (S22 Fig). Thus, unless one is careful, one may not even know what one is measuring!

Rapid evolution need not be unique to our engineered community of “broken” strains. Indeed, rapid evolution has been observed in phage–bacteria communities in aquatic environments and in acidophilic biofilms [59–61]. Rapid evolution is not surprising: given the large population sizes of microbial populations, mutants can pre-exist [62]. These pre-existing mutants can quickly take over in novel environments (e.g., exposure to evolving predators or to man-made pollutants and drugs) where the ancestor is ill adapted.

Choosing the right level of abstraction is yet another important consideration during model building, because different levels of abstraction show trade-offs between generality, realism, and precision [63]. When the level of abstraction is chosen properly, even complex biological phenomena can be described by simple and predictive equations. For example, a simplified model considering negative feedback regulation of carbon intake in *E*. *coli* quantitatively predicted cell growth rate on two carbon sources based on growth rates on individual carbon sources using only one single parameter that is fixed by experiments [64]. For CoSMO, one could construct a complex model that, for example, considers physiological and genetic networks of each cell to account for the dependence of phenotypes on the environment and on evolution. However, this would require making numerous assumptions and measuring even more numerous parameters. In the absence of free parameters, quantitative matching between model predictions and experimental results provides strong evidence that no additional complexity is required to explain the biological phenomenon of interest.

Once the right level of abstraction is chosen, a good model can serve multiple purposes [65–67], especially when coupled with quantitative measurements. First, a model suggests which parameters need to be carefully measured. For example, for spatial CoSMO growth rate, parameters such as diffusion coefficients are not critical (S23 Fig), but metabolite release and consumption parameters are (Eq 5). Second, a useful model not only explains existing data but also makes extrapolative predictions accurately. An example is the quantitative theory of the *lac* operon in *E*. *coli* ([68–70]). Finally, model–experiment discrepancy exposes knowledge gaps. When predicting CoSMO growth rate, the missing piece was environment-sensitive phenotypes.

Our approach can be applied to communities where interaction mechanisms can be inferred from genetic analysis. For example, we have applied this approach to understand an evolved metabolic interaction. Specifically, we observed that a single yeast population evolutionarily diverged into two genetically distinct subpopulations [71]. One subpopulation acquired a *met−* mutation that prevented the synthesis of organosulfurs and thus must rely on the *MET+* subpopulation for organosulfurs (which are essential for viability). Similar to this work, we first identified the released organosulfurs to be mainly glutathione and glutathione conjugates, using liquid chromatography–mass spectrometry. Because glutathione and glutathione conjugates were consumed by *met−* cells in a similar fashion, we “lump-summed” organosulfurs and quantified them in terms of “glutathione equivalents.” We then determined that organosulfurs were likely released by live cells, and quantified organosulfur release rate at various *MET+* growth rates. Finally, we quantified organosulfur consumption per birth of *met−* cell. These measurements allowed us to understand the steady-state ratio of the two subpopulations [71].

In summary, despite the many challenges, quantitative modeling of cell communities is possible. Importantly, by eliminating free parameters through direct experimental quantification, we arrive at two possibilities, both useful: quantitative matching between model predictions and experiments would provide strong evidence that no additional complexity is required to explain the biological phenomenon of interest. Significant mismatching between predictions and experiments would motivate us to look for the important missing pieces.

## Methods

### Strains and growth medium

We constructed CoSMO in the RM11 background due to its lower rate of mitochondrial mutation [48] compared with the S288C background used in our earlier studies [17]. Thus, phenotypes measured here differed from those measured in strains of the S288C background [17,32]. We introduced desired genetic modifications into the ancestral RM11 background via transformation [72,73] (S1 Table). Strains were stored at −80°C in 15% glycerol.

We used rich medium YPD (10 g/L yeast extract, 20 g/L peptone, 20 g/L glucose) in 2% agar plates for isolating single colonies. Saturated YPD overnight liquid cultures from these colonies were then used as inocula to grow exponential cultures. To prevent purines from being yield limiting, we supplemented YPD with 100 μM hypoxanthine for *A*^*−*^*L*^*+*^ cells. We sterilized YPD media by autoclaving. YPD overnights were stored at room temperature for no more than 4–5 d prior to experiments.

We used defined minimal medium SD (6.7 g/L Difco yeast nitrogen base w/o amino acids, 20 g/L glucose) for all experiments [74], with supplemental metabolites as noted [73]. To achieve higher reproducibility, we sterilized SD media by filtering through 0.22-μm filters. To make SD plates, we autoclaved 20 g/L Bacto agar or agarose in H_2_O and, after autoclaving, supplemented equal volume of sterile-filtered 2-fold concentrated SD. CoSMO steady-state growth rates on agar (which contains trace contaminants of metabolites) and agarose generate similar results.

### Strain culturing and preconditioning

All culturing, unless otherwise noted, was performed at 30°C in a well-mixed environment where culture tubes were inserted sideways into a roller drum (Model TC-7, New Brunswick Scientific, Edison, NJ). *L*^*−*^*A*^***+***^ cells were pregrown to exponential phase (OD_600_ generally less than 0.4 in 13-mm culture tubes, or <2.8 × 10^7^ cells/mL) in SD supplemented with excess (164 μM) lysine and washed 3–5 times with SD. In microscopy assays, when noted, we starved *L*^*−*^*A*^***+***^ cells for 4 h to deplete intracellular lysine storage. Otherwise, we did not prestarve *L*^*−*^*A*^***+***^. *A*^*−*^*L*^***+***^ cells were pregrown to exponential phase in SD supplemented with excess hypoxanthine (100 μM) or excess adenine (108 μM) as noted, washed 3–5 times with SD, and prestarved in SD for 24 h to deplete cellular purine storage. We pre-starved *A*^*−*^*L*^***+***^ to reduce CoSMO growth lag (S2 Fig), thus facilitating quantification of CoSMO growth rate. To be consistent, we also prestarved *A*^*−*^*L*^***+***^ during phenotype quantification.

### Flow cytometry

We prepared fluorescent bead stocks (3-μm red fluorescent beads Cat R0300, Thermo Fisher Scientific, Waltham, MA). Beads were autoclaved in a factory-clean glass tube, diluted into sterile 0.9% NaCl, and supplemented with sterile-filtered Triton X-100 to a final 0.05% (to prevent beads from clumping). We sonicated beads and kept them in constant rotation to prevent settling. We quantified bead concentrations by counting beads via hemacytometer and Coulter counter. Final bead stock was generally 4–8 × 10^6^/mL.

Culture samples were diluted to OD 0.01–0.1 (7 × 10^5^–7 × 10^6^/mL) in Milli-Q H_2_O in unautoclaved 1.6-mL Eppendorf tubes. A total of 90 μL of the diluted culture sample was supplemented with 10 μL bead stock and 2 μL of 1 μM ToPro 3 (T-3605, Molecular Probes, Eugene, OR), a nucleic acid dye that only permeates compromised cell membranes (dead cells). Sample preparation was done in a 96-well format for high-throughput processing.

Flow cytometry of the samples was performed on Cytek (Fremont, CA) DxP Cytometer equipped with 4 lasers, 10 detectors, and an autosampler. Fluorescent proteins GFP, Citrine, mCherry, TagBFP-AS-N (Evrogen, Moscow, Russia), and ToPro are respectively detected by a 50-mW 488-nm laser with 505/10 (i.e., 500–515-nm) detector, a 50-mW 488-nm laser with 530/30 detector, a 75-mW 561-nm laser with 615/25 detector, a 50-mW 408-nm laser with 450/50 detector, and a 25-mW 637-nm laser with 660/20 detector. Each sample was run in triplicates and individually analyzed using FlowJo software to identify numbers of events of beads, dead cells, and various live fluorescent cells. Densities of various populations were calculated from the cell–bead ratios. We then calculated the mean cell density from triplicate measurements, with the coefficient of variation generally within 5%–10%.

### HPLC

All HPLC measurements were done on a Shimadzu (Kyoto, Japan) Nexera X2 series ultra-performance HPLC (UHPLC) system. All supernatant samples were filtered (0.22-μm filter). For standards, we made a high-concentration solution, filtered it, and stored it at −80°C. Prior to an HPLC run, we diluted the stock to various concentrations in filtered H_2_O.

To quantify lysine, a 100-μL sample was loaded into an Agilent (Santa Clara, CA) 250 μL pulled point glass small volume insert (part number 5183–2085), which was then placed inside a Shimadzu 1.5 mL 12 × 32 mm autosampler vial (part number 228-45450-91). This vial was then placed into an autosampler (Nexera X2 SIL-30AC). Prior to injection into the column, samples were derivatized at 25°C with freshly made derivatization reagents in the autosampler using a programmed mixing method as follows. A total of 7.5 μL of sample was removed and placed into a separate reaction small volume insert and vial. Next, 45 μL of mercaptopropionic acid (10 μL per 10 mL 0.1 M sodium borate buffer, pH 9.2) and 22 μl of *o*-phthaladehyde (10 mg per 5 mL 0.1 M sodium borate buffer, pH 9.2) were added to this vial, mixed, and incubated for 1 min. A total of 10 μL of 9-fluorenyl methyl chloroformate (4 mg per 20 mL acetonitrile, HPLC grade) was then added, and the sample was remixed and incubated for 2 min. Finally, 10 μL of the reaction mixture was injected onto Phenomenex (Torrance, CA) Kinetex 2.6 μm EVO C18 100 Å LC Column (150 × 3.0 mm, part number 00F-4725-Y0) fitted with a SecurityGuard ULTRA Holder for UHPLC Columns (2.1 to 4.6 mm, part number AJ0-9000) and a SecurityGuard ULTRA cartridge (3.0-mm internal diameter, part number AJ0-9297). SecurityGuard ULTRA cartridge (precolumn) was periodically replaced in the event of pressure reading exceeding the manufacturer’s specifications.

Compounds were eluted from the column using a gradient of HPLC-grade Solution A (73 mM potassium phosphate, pH 7.2) and Solution B (50:50 acetonitrile/methanol). Solution A was filtered through a 0.2-μm filter prior to use. The percentage of solution B follows the following program: 0–2 min, 11%; 2–4 min, 17%; 4–5.5 min, 31%; 5.5–10 min, 32.5%; 10–12 min, 46.5%; and 12–15.5 min, 55%. The flow rate is maintained at 0.1 mL/min. The column was then flushed with 100% solution B for 5 min and re-equilibrated for 5 min with 11% solution B at 0.8 mL/min. The column was maintained at a running temperature of 35°C in a Nexera X2 CTO-20A oven. Absorbance measurements at 338 nm were measured using a high-sensitivity flow cell for a SPD-M30A UV-Vis detector.

For purines, we used the above protocol without the derivatization steps. Instead, a 5–10-μL sample was directly injected onto the column.

### Bioassays

We used a yield-based bioassay for relatively high metabolite concentrations (≥5 μM for lysine and ≥2 μM for hypoxanthine). For lower concentrations, we used a rate-based bioassay with a sensitivity of 0.1 μM for both lysine and hypoxanthine. When necessary, we diluted the sample to get into the assay linear range.

In the yield bioassay, a 75-μL sample filtered through a 0.2-μm filter was mixed with an equal volume of a master mix containing 2-fold concentrated SD (to provide fresh medium) as well as tester cells auxotrophic for the metabolite of interest (about 1 × 10^4^ cells/mL, WY1335 for lysine or WY1340 for hypoxanthine) in a flat-bottom 96-well plate. We then wrapped the plate with parafilm and allowed cells to grow to saturation at 30°C for 48 h. We resuspended cells using a Thermo Scientific Teleshake (setting #5 for about 1 min) and read culture turbidity using a BioTek Synergy MX plate reader. Within each assay, SD supplemented with various known concentrations of metabolite was used to establish a standard curve that related metabolite concentration to final turbidity (e.g., S3A Fig). From this standard curve, the total concentration of metabolites that can support auxotroph growth in an unknown sample could be inferred.

The rate bioassay was used for samples with low metabolite concentrations. For example, to measure lysine concentration in a lysine-limited chemostat, we mixed 150 μL filtered sample with an equal volume of master mix containing 2-fold concentrated SD and *L*^*−*^*A*^***+***^ tester cells (about 1 × 10^4^ cells/mL) in a flat-bottom 96-well plate. As our tester strain for lysine, we used an evolved clone (WY 2270) isolated after *L*^*−*^*A*^***+***^ had grown for tens of generations under lysine limitation. This clone displayed increased affinity for lysine due to an *ecm21* mutation and duplication of Chromosome 14. Growth rates of the tester strain in SD supplemented with various known concentrations of lysine and in the unknown sample were measured using a microscopy assay (Methods, “Microscopy quantification of growth phenotypes”). The growth rate of WY 2270 tester cells scaled linearly with lysine concentrations up to 1 μM (S6A Fig). Similarly, for hypoxanthine, we used an evolved *A*^*−*^*L*^***+***^ strain (WY1600) as the tester strain. The linear range was up to about 0.3 μM (S6B Fig). From the standard curve, we could infer the metabolite concentration of a sample.

### Extraction of intracellular metabolites

To extract intracellular metabolites, we poured a cell culture onto a 0.45-μm nitrocellulose membrane filter (Cat 162–0115, BioRad, Hercules, CA) in a reusable filtration device (glass microanalysis 25-mm vacuum filter holder with a 15-mL funnel, Product FHMA25, Southern Labware, Cumming, GA), applied vacuum to drain the supernatant, transferred the filter into extraction solution (40% acetonitrile, 40% methanol, and 20% water), vortexed to dislodge cells, and then removed the filter. This sequence was carried out as rapidly as possible (<10 s). We then flash-froze the extraction solution in liquid nitrogen and allowed it to thaw at −20°C. After thawing, we subjected the solution to five rounds of the following: vortexing for 1 min, and incubating on ice for 5 min between each vortexing. We then spun down the solution in a refrigerated centrifuge for 10 min at 14,000 rpm to pellet membrane-permeabilized cells as well as any membrane filter bits that may have disintegrated into the extraction solution. We transferred the supernatant containing soluble cell extract to a new tube. In order to make sure that all soluble components were extracted, we resuspended the cell pellet in a half volume of fresh extraction solution and subjected cells to another round of the same procedure (flash-freezing, five rounds of vortexing–ice incubation, and centrifugation). We then removed the supernatant and added it to the original supernatant. We then dried off the extraction solution in a centrifugal evaporator and resuspended soluble components in water. This resultant solution could then be assayed for metabolite concentrations. When properly dried, extracts did not contain inhibitors that might interfere with bioassays (S5 Fig).

For *L*^*−*^*A*^*+*^, cells from 19-mL cultures (4 × 10^5^–4 × 10^6^ cells/mL) were resuspended in 3 mL extraction buffer. One third of the sample was further processed, and extracted metabolites were resuspended in 0.5 mL water. For *A*^*−*^*L*^*+*^, metabolites from 1–5-mL cultures (1–6 × 10^6^ cells/mL) were extracted and resuspended in 1 mL water.

### Microscopy quantification of growth phenotypes

See [27] for details on microscopy experimental setup, method validation, and data analysis. Briefly, cells were diluted to low densities to minimize metabolite depletion during measurements. Dilutions were estimated from culture OD measurement to result in 1,000–5,000 cells inoculated in 300 μL SD medium supplemented with different metabolite concentrations in wells of a transparent flat-bottom microtiter plate (e.g., Costar 3370). We filled the outermost wells with water to reduce evaporation.

Microtiter plates were imaged periodically (every 0.5–2 h) under a 10× objective in a Nikon (Melville, NY) Eclipse TE-2000U inverted fluorescence microscope. The microscope was connected to a cooled CCD camera for fluorescence and transmitted light imaging. The microscope was enclosed in a temperature-controlled chamber set to 30°C. The microscope was equipped with motorized stages to allow z-autofocusing and systematic xy-scanning of locations in microplate wells, as well as motorized switchable filter cubes capable of detecting a variety of fluorophores. Image acquisition was done with an in-house LabVIEW program, incorporating bright-field autofocusing [27] and automatic exposure adjustment during fluorescence imaging to avoid saturation. Condensation on the plate lid sometimes interfered with autofocusing. Thus, we added a transparent “lid warmer” on top of our plate lid [27] and set it to be 0.5°C warmer than the plate bottom, which eliminated condensation. We used an ET DsRed filter cube (Exciter: ET545/30x, Emitter: ET620/60m, Dichroic: T570LP) for mCherry-expressing strains and an ET GFP filter cube (Exciter: ET470/40x, Emitter: ET525/50m, Dichroic: T495LP) for GFP-expressing strains.

Time-lapse images were analyzed using an ImageJ plug-in, Bioact [27]. Bioact measured the total fluorescence intensity of all cells in an image frame after subtracting the background fluorescence from the total fluorescence. A script plotted background-subtracted fluorescence intensity over time for each well to allow visual inspection. If the dynamics of four positions looked similar, we randomly selected one to inspect. In rare occasions, all four positions were out of focus and were not used. In a small subset of experiments, a discontinuous jump in data appeared in all four positions for unknown reasons. We did not calculate rates across the jump. Occasionally, one or two positions deviated from the rest. This could be due to a number of reasons, including shift of focal plane, shift of field of view, black dust particles, or bright dust spots in the field of view. The outlier positions were excluded after inspecting the images for probable causes. If the dynamics of four positions differed because of cell growth heterogeneity at low concentrations of metabolites, all positions were retained.

We normalized total intensity against that at time zero, and averaged across positions. We calculated growth rate over three to four consecutive time points and plotted the maximal net growth rate against metabolite concentration (e.g., S4 Fig). If maximal growth rate occurred at the end of an experiment, then the experimental duration was too short and data were not used. For *L*^*−*^*A*^***+***^, the initial stage (3–4 h) residual growth was excluded from analysis. For *A*^*−*^*L*^***+***^, because cells had already been prestarved, fluorescence intensity did not continue to increase in the absence of supplements.

For longer *A*^*−*^*L*^***+***^ imaging (30+ h), we observed two maximal growth rates at low hypoxanthine concentrations (e.g., about 0.4 μM), possibly due to mutant clones. We used the earlier maximal growth rate even if it was lower than the later maximal growth rate, because the latter was probably caused by faster-growing mutants.

### Chemostat culturing

We have constructed an eight-vessel chemostat with a design modified from [75]. For details of construction, modification, calibration, and operation, see [58].

For *L*^*−*^*A*^***+***^, due to rapid evolution, we tried to devise experiments so that live and dead populations quickly reached steady state. Two conditions seemed to work well. In both, we first calculated the expected steady-state cell density by dividing the concentration of lysine in the reservoir (20 μM) by fmole lysine consumed per new cell. Condition 1 consisted of the following: wash exponentially growing cells to completely remove any extracellular lysine and inoculate the full volume (19 mL) at 100% of expected steady-state density. Start chemostat to drip in lysine at the prespecified flow rate. Condition 2 consisted of the following: wash exponentially growing cells to remove extracellular lysine and inoculate 50%–75% of the volume at 1/3 of the expected steady-state density. Fill the rest of the 19-mL vessel with reservoir media (resulting in less than the full 20 μM of starting lysine, but more than enough for maximal initial growth rate, about 10–15 μM). The two conditions yielded similar results (Fig 3). We predominantly used Condition 2.

We set the pump flow rate to achieve the desired doubling time *T* (19 mL * ln(2)/*T*). We collected and weighed waste media for each individual culturing vessel to ensure that the flow rate was correct (i.e., total waste accumulated over time *t* was equal to the expected flow rate * t). We sampled cultures periodically to track population dynamics using flow cytometry (Methods, “Flow cytometry”), filtered supernatant through a 0.45-μm nitrocellulose filter, and froze the supernatant for metabolite quantification at the conclusion of an experiment (Methods, “Bioassays”). At the conclusion of an experiment, we also tested input media for each individual culturing vessel to ensure sterility by plating a 300-μL aliquot on an YPD plate and checking for growth after 2 d of growth at 30°C. If a substantial number of colonies grew (>5 colonies), the input line was considered contaminated and data from that vessel were not used.

*A*^*−*^*L*^***+***^ cells exponentially growing in SD + 100 μM hypoxanthine were washed and prestarved for 24 h. We then filled the chemostat culturing vessel with starved cells in SD at 100% of the expected starting density and pumped in fresh medium (SD + 20 μM hypoxanthine) to achieve the desired doubling time. Cultures were otherwise treated as described above for *L*^*−*^*A*^*+*^.

For most experiments, we isolated colonies from the end time point and checked percentage evolved (Methods, “Detecting evolved clones”). For *L*^*−*^*A*^***+***^, we only analyzed time courses for which >90% of population remained ancestral. For *A*^*−*^*L*^***+***^, significant levels of mutants were generated before and throughout quantification (S9 Fig). Because quantified phenotypes did not correlate strongly with the percentage of mutants (S11 Fig) and because mutants accumulated similarly during chemostat measurements and during CoSMO growth rate measurements (S9B Fig), we used the time window for CoSMO growth rate quantification (about 96 h) in *A*^*−*^*L*^***+***^ chemostat experiments.

### Quantifying phenotypes in chemostats

We illustrate how we quantify release rate, consumption amount per birth, and death rate in chemostats, using *L*^*−*^*A*^***+***^ as an example. In a lysine-limited chemostat, live cell density [*L*^−^*A*^+^]_*live*_ is increased by birth (at a rate *b*_*L*_) and decreased by death (at a rate *d*_*L*_) and dilution (at a rate *dil*):

$$\frac{d[L^{-}A^{+}{]}_{live}}{dt}=(b_{L}-d_{L}-dil)[L^{-}A^{+}{]}_{live}$$ (6)

Dead cell density [*L*^−^*A*^+^]_*dead*_ is increased by death and decreased by dilution

$$\frac{d[L^{-}A^{+}{]}_{dead}}{dt}=d_{L}[L^{-}A^{+}{]}_{live}-dil[L^{-}A^{+}{]}_{dead}$$ (7)

*L*, lysine concentration in the culturing vessel, is increased by the supply of fresh medium (at concentration *L*_*0*_) and decreased by dilution and consumption (with each birth consuming *c*_*L*_ amount of lysine).

$$\frac{dL}{dt}=L_{0}\cdot dil-L\cdot dil-c_{L}b_{L}[L^{-}A^{+}{]}_{live}$$ (8)

Finally, hypoxanthine concentration *A* is increased by release (either from live cells at *r*_*A*_ per live cell per h or from dead cells at *r*_*A*,*d*_ per death) and decreased by dilution.

$\frac{dA}{dt}=r_{A}\cdot [L^{-}A^{+}{]}_{live}-dil\cdot A$ (9, if live release)

or

$\frac{dA}{dt}=r_{A,d}\cdot d_{L}\cdot [L^{-}A^{+}{]}_{live}-dil\cdot A$ (10, if dead release)

Note that at the steady state (denoted by subscript *ss*), net growth rate is equal to dilution rate (setting Eq 6 to zero):

$$b_{L}-d_{L}=dil$$ (11)

To measure metabolite consumed per cell at steady state, we set Eq 8 to zero

$$c_{L}=\frac{L_{0}\cdot dil-L\cdot dil}{b_{L}[L^{-}A^{+}{]}_{live,ss}}∼\frac{L_{0}}{[L^{-}A^{+}{]}_{live,ss}}$$ (12)

Here, the approximation holds because the concentration of lysine in chemostat (*L*) is much smaller than that in reservoir (*L*_*0*_) and because birth rate *b*_*L*_ is similar to dilution rate *dil*.

To measure death rate at steady state, we set Eq 7 to zero and get

$$d_{L}=dil\frac{[L^{-}A^{+}{]}_{dead,ss}}{[L^{-}A^{+}{]}_{live,ss}}$$ (13)

Thus, we can measure death rate by measuring the steady-state dead and live population densities averaged over time.

To measure release rate at steady state, we can set Eq 9 to zero and obtain

$$r_{A}=\frac{dil\cdot A_{ss}}{[L^{-}A^{+}{]}_{live,ss}}$$ (14)

Alternatively, we can use both the pre–steady-state and steady-state chemostat dynamics to quantify release rate and death rate if these rates are constant. For release rate, we multiply both sides of Eq 9 with *e*^*dil*∙*t*^

$$\frac{e^{dil\cdot t}dA}{dt}+dil\cdot Ae^{dil\cdot t}=r_{A}\cdot [L^{-}A^{+}{]}_{live}e^{dil\cdot t}\;or\frac{d(e^{dil\cdot t}A)}{dt}=r_{A}\cdot [L^{-}A^{+}{]}_{live}e^{dil\cdot t}.$$

Because the initial *A* is zero, we have

$$Ae^{dil\cdot t}=r_{A}\int_{0}^{t}[L^{-}A^{+}{]}_{live}e^{dil\cdot \tau }d\tau$$ (15)

How do we calculate $\int_{0}^{t}f(\tau )d\tau$ from experimental data? The value of integral is always zero at *t* = 0. For each time point *t* + *Δt*, the integral is the integral at the previous time point *t* (i.e., $\int_{0}^{t}f(\tau )d\tau$) plus $\Delta t\frac{f(t)+f(t+\Delta t)}{2}$. If we plot *Ae*^*dil*∙*t*^ against $\int_{0}^{t}[L^{-}A^{+}{]}_{live}e^{dil\cdot \tau }d\tau$, we should get a straight line through the origin with a slope of *r*_*A*_ (S14 Fig, blue).

Similarly to Eq 7, if death rate is constant, we have

$$[L^{-}A^{+}{]}_{dead}e^{dil\cdot t}=[L^{-}A^{+}{]}_{dead}(t=0)+d_{L}\int_{0}^{t}[L^{-}A^{+}{]}_{live}e^{dil\cdot \tau }d\tau$$ (16)

If we plot $[L^{-}A^{+}{]}_{dead}e^{dil\cdot t}$ against $\int_{0}^{t}[L^{-}A^{+}{]}_{live}e^{dil\cdot \tau }d\tau$, we should get a straight line with a slope of *d*_*L*_ (S14 Fig, gray).

The two methods (using only the steady-state data versus performing linear regression on the entire data range) yielded similar results. We have opted for the latter method because it takes advantage of pre–steady-state data.

### Detecting evolved clones

To detect evolved clones in an *L*^*−*^*A*^***+***^ culture, we diluted it to <1,000 cells/mL and plated 300 μL on a YPD plate and allowed colonies to grow for 2–3 d. We randomly picked 20–50 colonies to inoculate into YPD and grow saturated overnights. We diluted each saturated overnight 1:6,000 into SD + 164 μM lysine and allowed cultures to grow overnight at 30°C to exponential phase. We washed cells 3× with SD, starved them for 4–6 h to deplete vacuolar lysine stores, and diluted each culture so that a 50-μL spot had several hundred cells. We spotted 50 μL on an SD plate supplemented with 1.5 μM lysine (10 spots/plate) and allowed these plates to grow overnight. When observed under a microscope, evolved cells with increased lysine affinity would grow into “microcolonies” of about 20–100 cells, while the ancestral genotype failed to grow (S7C Fig). Occasionally an intermediate phenotype was observed where smaller microcolonies with variable sizes formed, and this phenotype was counted as evolved as well. For a high-throughput version of this assay, we diluted YPD saturated culture 10,000× into SD and waited for 3 h at room temperature. We then directly spotted 50 μL on SD plates supplemented with 1.5 μM lysine. Ancestral cells formed ≤10-cell clusters, but we could still clearly distinguish ancestor versus evolved clones.

To detect evolved clones in an *A*^*−*^*L*^***+***^ culture, we took advantage of the observation that evolved clones with improved affinity for hypoxanthine grew slowly when hypoxanthine concentration was high (S8A Fig). A similar fitness trade-off has been observed for *L*^*−*^*A*^***+***^ [43] and in many other examples [76–79]. From an *A*^*−*^*L*^***+***^ culture, we randomly picked colonies and made individual YPD overnights in a 96-well plate. We diluted YPD overnights 1:3,600-fold into SD + 100 μM hypoxanthine or 108 μM adenine, and grew for 16–24 h. Some of these cultures were not turbid while other cultures and the ancestor reached near saturation (S8B Fig). We considered these low-turbidity cultures as evolved, and they generally grew faster than the ancestor in low (0.4 μM) hypoxanthine (S8A Fig, compare blue, gray, and green against magenta).

### Starvation release assay

For *L*^*−*^*A*^***+***^, we washed exponential phase cells and diluted each sample to OD 0.1 to roughly normalize cell density. We took an initial cell density reading of each sample by flow cytometry, wrapped tube caps in parafilm to limit evaporation, and incubated in a rotator at 30°C. Prep time (from the start of washing to the initial cell density reading) took approximately 2 h, during which time the majority of residual growth had taken place. At each time point, we measured live and dead cell densities by flow cytometry; we froze an aliquot of supernatant where supernatant had been separated from cells by filtering through sterile nitrocellulose membrane. We concluded the assay after approximately 24 h, generally aiming for time points every 6 h. At the conclusion of the assay, we quantified hypoxanthine concentration for each sample using the yield bioassay (Methods, “Bioassays”). The slope of the linear regression of integrated live cell density over time (cells/mL * h) versus hypoxanthine concentration (μM) gave us the release rate.

For *A*^*−*^*L*^***+***^, the starvation release assay was similar, except that the assay lasted longer with less frequent time points to accommodate the longer assay. Pregrowth in 108 μM Ade versus 100 μM hypoxanthine generated similar release rates, and thus we pooled the data.

### Evolutionary dynamics of mutant *A*^*−*^*L*^*+*^

Mutant *A*^*−*^*L*^***+***^ clones were alike, and they grew about 50% slower than the ancestor in excess hypoxanthine (S8A Fig). This has allowed us to rapidly quantify mutant abundance (S8B Fig; Methods, “Detecting evolved clones”). The high abundance of mutants during exponential growth is surprising, especially given the large (about 50%) fitness disadvantage of mutants in excess hypoxanthine (S8A Fig). Whole-genome sequencing of a randomly chosen evolved *A*^*−*^*L*^***+***^ clone (WY2447) revealed evidence for aneuploidy (S8C Fig; Methods, “Genomic analysis”). Assuming a chromosomal mis-segregation rate of 0.01/generation/cell and incorporating the fitness difference between ancestor and mutant in various hypoxanthine concentrations (S10A Fig), our mathematical models (S2 Code; S3 Code) qualitatively captured experimental observations (S10B and S10C Fig). This extraordinarily high mutation rate is possibly due to an imbalance in purine intermediates in a purine auxotroph and is in line with the highest chromosomal mis-segregation rate observed in chromosome transmission fidelity mutants (up to 0.015/generation/cell) [80]. In low concentrations of hypoxanthine (<1 μM), the fitness difference between mutant and ancestral *A*^*−*^*L*^***+***^ varied from 30% to 70% (right panel of S10A Fig), consistent with the dynamics of mutant *A*^*−*^*L*^***+***^ in chemostats.

Ancestral and evolved clones exhibited distinct phenotypes (S8A and S8D Fig). However, measured phenotype values were not significantly correlated with the percentage of mutants at the end of an experiment. This was due to the relatively narrow spread in the percentage of mutants and the relatively large measurement errors (S11 Fig).

### Measuring consumption in batch cultures

To measure consumption in exponential cultures, we diluted exponentially growing cells to about 1 × 10^6^ cell/mL in SD supplemented with about 100 μM metabolite and measured cell density (Methods, “Flow cytometry”) and metabolite concentration (Methods, yield assay in “Bioassays”) every hour over 6 h. For an exponential culture of size *N(t)* growing at a rate *g* while consuming metabolite *M*, we have

$$\frac{dN}{dt}=gN$$

$$\frac{dM}{dt}=-cgN.$$

Thus, $\frac{dM}{dt}=-c\frac{dN}{dt}$. Integrating both sides, we have *M(t) − M(0) = −c(N(t) − N(0))*. Thus, if we plot *M(t)* against *N(t)*, the slope is consumption per birth. We disregarded time points after which M had declined to less than 10 μM, even though cells could still grow at the maximal growth rate.

We also measured consumption after cells fully “saturated” the culture and used intracellular stores for residual growth. We starved exponentially growing cells (3–6 h for *L*^*−*^*A*^***+***^, 24 h for *A*^*−*^*L*^***+***^) to deplete initial intracellular stores and inoculated about 1 × 10^5^ cells/mL into various concentrations of the cognate metabolite up to 25 μM. We incubated for 48 h and then measured cell densities by flow cytometry. We performed linear regression between input metabolite concentrations and final total cell densities within the linear range, forcing the regression line through origin. Consumption per birth in a saturated culture was quantified from 1/slope.

### Measuring the upper bound of release rate in excess metabolites

To measure release rate in an exponentially growing population in excess metabolites, we note that

$$\frac{dM}{dt}=rN$$

where *M* is metabolite concentration, *r* is the release rate, and *N* is live population density. Let *g* be growth rate; then, after integration, we have

$$M(T)=\int_{0}^{T}rNdt=\int_{0}^{T}rN(0)e^{gt}dt=\frac{r}{g}N(0)e^{gt}|{}_{0}^{T})\approx \frac{r}{g}N(T).$$

The approximation holds when *N(T)>>N(0)*, which is true experimentally.

We grew cells in excess metabolite (lysine or hypoxanthine) exponentially to time *T* when OD_600_ < 0.5 (i.e., <1.6 × 10^7^/mL). Supernatants were assayed for released metabolite using the rate bioassay (Methods, “Bioassays”). Because *M(T)* was below the sensitivity of detection (about 0.1 μM; S6 Fig) for both strains, we used 0.1 μM as *M(T)*, growth rate (0.47–0.48/h for *L*^*−*^*A*^***+***^ and 0.43–0.44/hr for *A*^*−*^*L*^***+***^), and *N(T)* (1.4–1.6 × 10^7^/mL) to calculate the upper bound for release rate *r*.

### Genomic analysis

High-quality genomic DNA was extracted using the QIAGEN (Hilden, Germany) Genomic-tip 20G kit (CAT Number 10223) or the Zymo Research (Irvine, CA) YeaStar Genomic DNA Kit (CAT Number D2002). DNA fragmentation and libraries were prepared [81] using a Nextera DNA Sample Preparation Kit (Illumina, San Diego, CA) with 96 custom bar code indices [82] and TruSeq Dual Index Sequencing Primers. Libraries were pooled and multiplexed on a HiSeq2000 lane (Illumina) for 150-cycle paired-end reading. A custom analysis pipeline written in Perl incorporated the bwa aligner [83] and samtools [84] for alignment file generation, GATK for SNP/indel calling [85], and cn.MOPs for local copy number variant calling [86]. Finally, a custom Perl script using vcftools [87] was used to automate the comparison of an evolved clone with its ancestor. All called mutations were validated by visual inspection in the IGV environment [88].

Ploidy was calculated using custom python and R scripts. Read depth was counted for each base and averaged within consecutive 1,000-bp windows. Then, the average coverage of each 1,000-bp window was normalized against the median of these values from the entire genome and log_2_ transformed. Transformed data were plotted as box plots for each chromosome/supercontig. All code is publicly available at https://github.com/robingreen525/ShouLab_NGS_CloneSeq.

### Calculating death rate in nonlimited batch culture

We grew cells to exponential phase in SD + excess supplements. While still at a low density (<10^7^ cells/mL), we measured live and dead cell densities using flow cytometry to yield a dead/live ratio. Because the percentage of dead cells was small, we analyzed a large volume of sample via flow cytometry to ensure that at least 400 ToPro3-stained dead cells were counted so that the sampling error ($\frac{2\sqrt{N}}{N}$) was no more than 10%. We also calculated growth rates using optical density readings for the 2 h before and after flow cytometry measurement to yield the net growth rate, *g*. In exponentially growing cells,

$$\frac{dLive}{dt}=(b-d)\cdot Live$$

$$\frac{dDead}{dt}=d\cdot Live$$

where *b* and *d* are, respectively, birth and death rates of cells.

Thus, *Live* = *Live*(*t* = 0)*e*^(*b*−*d*)*t*^

$Dead∼\frac{d\cdot Live(t=0)}{b-d}e^{(b-d)t}=\frac{d}{b-d}Live$, or

*d* = (*b*−*d*) ∙ *Dead*/*Live*.

Thus, the ratio of dead to live cells is the ratio of death rate to net growth rate. The death rate of *lys2−* cells in excess lysine ranged from 10^−4^ to 10^−3^/h. This large variability persisted despite our using the same culture master mix to grow independent cultures.

### Quantifying spatial CoSMO growth dynamics

In all experiments, *L*^*−*^*A*^***+***^ cells were grown to exponential phase in SD plus lysine, and washed free of lysine. *A*^*−*^*L*^***+***^ cells were grown to exponential phase in SD plus hypoxanthine, washed, prestarved in SD for 24 h, and washed again. Prestarvation was intended to deplete cellular hypoxanthine storage and to shorten CoSMO growth lag (S2 Fig). We grew spatial CoSMO in two configurations: “column” versus “spotting.”

In the column setting, to prevent potential metabolite cross-contamination, we overfilled non-neighboring wells (i.e., 24 wells per deep 96-well plate) with 2 × SD + 2% agar and covered the surface with a sterile glass plate to form a flat agar surface with no air bubbles. After solidification, we removed the glass plate and removed extra agar between filled wells using sterile tweezers. This results in an agar depth of 3 cm. For the rest of the experiment, when not setting up or sampling, we covered the plate with a sterile lid suspended above the wells by thick toothpicks. We wrapped plates with parafilm to reduce agar drying. We mixed strains at a 1:1 ratio and filtered them through MF membrane (HAWP04700 from Millipore, Billerica, MA) to achieve a 3,000 cells/mm^2^ density on the filter (see [32] for details). We then punched 8-mm-diameter disks and transferred one disk to each agarose well, resulting in about 1.5 × 10^5^ cells/disk. For each time point, we used tweezers (ethanol flame sterilized between samples) to pick 2–3 disks, and suspended each in water prior to flow cytometry measurements.

In the spotting setting, in an 85-mm petri dish, we poured about 25 mL 2 × SD + 2% agarose + a small amount of lysine (generally 0.7 μM to minimize the lag phase during CoSMO growth) to achieve an agar/agarose depth of 5 mm. After solidification, we used a sterile blade to cut and remove 2-mm strips out of the agar to create six similarly sized sectors on the plate, with no agarose connections between them (S24A Fig). We inoculated plates by spotting 15 μL of strains at a 1:1 ratio onto plates, to result in about 4 × 10^4^ cells/patch (4-mm inoculum radius). Cells were grown and sampled as in the column setting, except that we cut out the agarose patch containing cells, submerged it in water, vortexed for a few seconds, and discarded the agarose.

For both setups, we used 9 × 10^7^ total cells as a cutoff for the CoSMO growth rate calculation. We used this cutoff because exponential CoSMO growth rate was observed beyond 9 × 10^7^ total cells, suggesting that no other metabolites were limiting by then.

### Simulating spatial CoSMO growth

We modified our previous individual-based spatial CoSMO model [32] so that in each time step, metabolite consumption and release of each cell scaled linearly with cell’s biomass to reflect exponential growth. The model used the parameters in Table 1. The release rate of lysine for each *A*^*−*^*L*^***+***^ cell at each time step was linearly interpolated based on the local concentration of hypoxanthine (S8 Table). We simulated CoSMO growth in two different settings: (1) cells were initially uniformly distributed on the surface of an agar column and (2) cells were initially spotted in the middle of an agar pad according to the experimental setup. The simulation domain used for setting (1) was 500 × 500 μm in the lateral *x* and *y* dimensions; for setting (2), the agarose domain was 800–960 μm on each side (5 μm/grid), and the size of the inoculation spot was 1/4 × 1/4 = 1/16 of the agarose domain. In both settings (1) and (2), the *z* dimension in simulation varied according to the experimental setup (5 mm–3 cm). For metabolite diffusion within the community, we used either a single diffusion coefficient (*D* = 360 μm^2^/s; S6 Code) or two diffusion coefficients (*D* = 360 μm^2^/s measured in agarose and *D* = 20 μm^2^/s measured in yeast community [32]; S7 Code). Both codes are for spotting inoculation, but the inoculation spot can be increased to cover the entire surface. Regardless of the simulation setup, we obtained a similar steady-state community growth rate.

### Calculating steady-state community growth rate

When CoSMO achieves the steady-state growth rate, both strains will grow at the same rate as the community (*g*_*comm*_). This means that *L* and *A* concentrations do not change, and Eqs 1–4 become

$$b_{L}-d_{L}=g_{comm}$$

$$b_{A}-d_{A}=g_{comm}$$

$$r_{L}[A^{-}L^{+}]=c_{L}b_{L}[L^{-}A^{+}]=c_{L}(g_{comm}+d_{L})[L^{-}A^{+}]$$

$$r_{A}[L^{-}A^{+}]=c_{A}b_{A}[A^{-}L^{+}]=c_{A}(g_{comm}+d_{A})[A^{-}L^{+}]$$

Combining the last two equations, we get

*r*_*A*_*r*_*L*_ = *c*_*A*_*c*_*L*_(*g*_*comm*_ + *d*_*L*_)(*g*_*comm*_ + *d*_*A*_).

Solving this, we get

$$g_{comm}=-\frac{\left(d_{A}+d_{L}\right)}{2}+\sqrt{\frac{r_{A}r_{L}}{c_{A}c_{L}}+\frac{(d_{A}-d_{L}{)}^{2}}{4}}.$$ (17)

For Model iii, given our parameter values (Table 1), $\frac{{\left(d_{A}-d_{L}\right)}^{2}}{4}\ll \frac{r_{A}r_{L}}{c_{A}c_{L}}$. Thus, we obtain

$$g_{comm}\approx -\frac{\left(d_{A}+d_{L}\right)}{2}+\sqrt{\frac{r_{A}r_{L}}{c_{A}c_{L}}}$$ (18; 5)

*r*_*L*_, lysine release rate of *A*^*−*^*L*^***+***^, varies with growth rate (Fig 7). When we focus on doubling times between 5.5 and 8 h, a range experienced by CoSMO, then we arrive at the following correlation (Fig 6B, green dotted line):

*r_L_* = 1.853−11.388*g_A_*, where *g*_*A*_ is the net growth rate of *A*^*−*^*L*^***+***^.

Because at steady state, growth rate, *g*_*A*_ = *g*_*comm*_, we have

$$g_{comm}\approx -\frac{d_{A}+d_{L}}{2}+\sqrt{\frac{r_{A}r_{L}}{c_{A}c_{L}}}=-\frac{0.015+0.0024}{2}+\sqrt{\frac{0.27(1.853-11.388*g_{comm})}{3.1*5.4}}$$

(*g*_*comm*_ + 0.0087)^2^ = (1.853−11.388*g_*comm*_)0.0161 = 0.0298−0.1833*g*_*comm*_. That is,

*g*_*comm*_^2^ + 0.200*g*_*comm*_−0.030 = 0.

$$\mathrm{Thus},\;g_{comm}=0.10/h$$ (19)

corresponding to a doubling time of 6.9 h.

To estimate the uncertainty in our prediction of *g*_*comm*_, we use the variance formula for error propagation. Specifically, let *f* be a function of *x*_*i*_ (*i* = 1, 2,…, *n*). Then, the error of *f*, *s*_*f*_, can be expressed as

$${s_{f}}^{2}=\sum {\left(\frac{\partial f}{\partial x_{i}}s_{x_{i}}\right)}^{2}$$

where $s_{x_{i}}$ is the uncertainty of *x*_*i*_.

Thus, for each of the six parameters in Eq 18, we divide its 95% confidence interval (Table 1) by 2 to obtain error *s*. For lysine release rate, *r*_*L*_, we use the value measured in chemostats with a 7-h doubling time, which closely corresponds to CoSMO doubling time.

1. ${\left(\frac{\partial g}{\partial d_{A}}\right)}^{2}{s_{dA}}^{2}=\frac{1}{4}(\frac{0.001}{2}{)}^{2}=6\times 10^{-8}$
2. ${\left(\frac{\partial g}{\partial d_{L}}\right)}^{2}{s_{dL}}^{2}=\frac{1}{4}(\frac{0.0006}{2}{)}^{2}\approx 2\times 10^{-8}$
3. ${\left(\frac{\partial g}{\partial r_{A}}\right)}^{2}{s_{rA}}^{2}={\left(\sqrt{\frac{r_{L}}{c_{A}c_{L}}}\frac{1}{2\sqrt{r_{A}}}s_{rA}\right)}^{2}=\frac{0.78}{3.1*5.4*0.27}{\left(\frac{1}{2}*\frac{0.02}{2}\right)}^{2}=4.3\times 10^{-6}$
4. ${\left(\frac{\partial g}{\partial r_{L}}\right)}^{2}{s_{rL}}^{2}={\left(\sqrt{\frac{r_{A}}{c_{A}c_{L}}}\frac{1}{2\sqrt{r_{L}}}s_{rL}\right)}^{2}=\frac{0.27}{3.1*5.4*0.78}{\left(\frac{1}{2}*\frac{0.08}{2}\right)}^{2}=8.3\times 10^{-6}$
5. ${\left(\frac{\partial g}{\partial c_{A}}\right)}^{2}{s_{cA}}^{2}={\left(\sqrt{\frac{r_{A}r_{L}}{c_{L}}}\frac{1}{2\sqrt{{c_{A}}^{3}}}s_{cA}\right)}^{2}=\frac{0.27*0.78}{5.4*3.1^{3}}{\left(\frac{1}{2}*\frac{0.124}{2}\right)}^{2}=1.3\times 10^{-6}$
6. ${\left(\frac{\partial g}{\partial c_{L}}\right)}^{2}{s_{cL}}^{2}={\left(\sqrt{\frac{r_{A}r_{L}}{c_{A}}}\frac{1}{2\sqrt{{c_{L}}^{3}}}s_{cL}\right)}^{2}=\frac{0.27*0.78}{3.1*5.4^{3}}{\left(\frac{1}{2}*\frac{0.26}{2}\right)}^{2}=1.8\times 10^{-6}$

Summing all terms and taking the square root, we have an error of 0.004 for *g*_*comm*_. Thus, the 95% confidence interval is ±0.01.

We did not calculate the uncertainty of our spatial simulation prediction, because we did not solve the spatial model analytically. However, given that predicted community growth rates with or without diffusion are similar (Fig 7), we expect that the two predictions should share similar uncertainty.

## Supporting information

S1 Fig Using *A*^*−*^*L*^*+*^ phenotypes measured in batch monocultures supplemented with adenine versus hypoxanthine did not affect model performance. “Exp”: community growth rates were calculated from seven independent experiments in a well-mixed environment (from about 30 h to 70–80 h) and averaged, with the error bar representing 2 standard deviations. “Model ii”: all model parameters were derived from *L*^*−*^*A*^***+***^ and *A*^*−*^*L*^***+***^ of the RM11 genetic background measured in batch monocultures. We predicted steady-state community growth rate either via quantifying the simulated post-lag dynamics (e.g., Fig 1B “Model ii”) (“Sim”) or via an analytical formula (Eq 17 in Methods) (“Cal”). The experimental and predicted doubling times were 6.5 and 4.3 h, respectively. Experimental data and model parameters are listed in S1 Data and S2 Table, respectively. Cal, calculation; Exp, experiment; Sim, simulation.
(TIF)

S2 Fig Prestarving *A*^*−*^*L*^*+*^ reduces the lag phase of community growth. Exponential *A*^*−*^*L*^***+***^ (WY1340) cells were washed free of hypoxanthine and either prestarved for 24 h in SD (solid lines) or not prestarved (dotted lines) before being mixed with exponentially grown and then washed *L*^*−*^*A*^***+***^ (WY1335) to form CoSMO. Prestarvation of *A*^*−*^*L*^***+***^ leads to less growth lag compared with no prestarvation. All data can be found in S8 Data. CoSMO, Cooperation that is Synthetic and Mutually Obligatory.
(TIF)

S3 Fig Inosine does not mediate the interaction from *L*^*−*^*A*^*+*^ to *A*^*−*^*L*^*+*^. (**A**) Hypoxanthine but not inosine is consumed by *A*^*−*^*L*^***+***^. The final turbidity of an *ade8−* (WY1340) tester strain increases with increasing concentrations of hypoxanthine (blue) and adenine (gray), but not inosine (brown). The slopes of the blue and gray lines are similar, suggesting that a similar amount of hypoxanthine and adenine are consumed to produce one new *A*^*−*^*L*^***+***^ cell. (**B**) Stimulation of the *A*^*−*^*L*^***+***^ (WY1340) growth rate by hypoxanthine (blue) is not affected by the presence of inosine at 1× (orange) or 10× (brown) concentration. All data can be found in S9 Data.
(TIF)

S4 Fig Hypoxanthine and adenine lead to quantitatively different growth phenotypes in *A*^*−*^*L*^*+*^. *A*^*−*^*L*^***+***^ cells grow faster when fed with adenine (red) than when fed with hypoxanthine (blue) when metabolite concentration is low (inset). *A*^*−*^*L*^***+***^ (WY1340) cells pregrown in SD + adenine or SD + hypoxanthine were washed into SD and prestarved for 24 h to deplete intracellular storage. Subsequently, adenine or hypoxanthine was supplemented at various concentrations, and the net growth rate was measured via fluorescence microscopy (Methods, “Microscopy quantification of growth parameters”). Red circles and squares: pregrown in adenine and incubated in adenine; red crosses: pregrown in hypoxanthine and incubated in adenine; blue circles and squares: pregrown in hypoxanthine and incubated in hypoxanthine; blue crosses: pregrown in adenine and incubated in hypoxanthine. Pregrowth in cognate metabolite versus noncognate metabolite does not make a difference (e.g., compare red circles with red crosses and blue circles with blue crosses, all of which were measured in the same experiment). All data can be found in S10 Data.
(TIF)

S5 Fig Cell extracts do not interfere with bioassays. Exponential *L*^*−*^*A*^***+***^ (WY1335) cells were starved in SD for 4 h to deplete intracellular storage of lysine. A total of 2.5 mL of starved culture at OD 0.2 was used to extract intracellular metabolites (“Extraction of intracellular metabolites” in Methods). The dried pellet was resuspended in about 1 mL H_2_O. In a separate experiment, exponential *L*^*−*^*A*^***+***^ were washed and prestarved in SD for 4 h. We then quantified the growth rates of *L*^*−*^*A*^***+***^ in SD supplemented with 1/3 volume of extracts (orange and blue) or water (black), as well as various concentrations of lysine (“Microscopy quantification of growth phenotypes” in Methods). The inclusion of extracts did not affect growth rates. All data can be found in S11 Data. OD, optical density at 600 nm; SD, Synthetic Dextrose minimal medium.
(TIF)

S6 Fig Using evolved clones to measure low concentrations of metabolites. (**A**) WY2270, an evolved *L*^*−*^*A*^***+***^ clone with significantly improved affinity for lysine, could detect sub–1 μM Lys. (**B**) WY1600, an evolved *A*^*−*^*L*^***+***^ clone with a significantly improved affinity for hypoxanthine, could detect sub–1 μM hypoxanthine. Vertical dotted blue lines mark detection limits. Circles and diamonds mark two independent replicates. All data can be found in S12 Data. Lys, lysine.
(TIF)

S7 FigCharacterization of evolved *L*^*−*^*A*^*+*^ clones.Whole-genome sequencing revealed that evolved *L*^*−*^*A*^***+***^ clones harbor mutations in genes such as *RSP5* (an E3 ubiquitin ligase) and *ECM21* (an arrestin-like adaptor for Rsp5) [43]. In a stressful environment, wild-type Ecm21 and Rsp5 proteins target cell surface permeases (including the high-affinity lysine permease, Lyp1) for ubiquitination [53]. Ubiquitinated permeases are then endocytosed and degraded in the vacuole [53]. The resulting amino acids are then transported to the cytoplasm for protein synthesis to help cells cope with stress [89]. In evolved cells with mutant *ecm21* or *rsp5*, lysine permease is stabilized. (**A**) Evolved *L*^*−*^*A*^***+***^ grows faster than the ancestor in lysine-limited chemostats. *L*^*−*^*A*^***+***^ with or without an *ecm21* deletion (WY2226 and WY1657, respectively) expressing different fluorescent proteins were competed in 8-h doubling time chemostats. The initial lysine concentrations in culturing vessels was 0 (black triangles) or 10–15 μM (brown circles). In all four chemostats, *ecm21* overtook the ancestor. The fitness difference between the two strains can be estimated: Let *E(t)* and *A(t)* be population densities of *ecm21* and the ancestor at time *t*, respectively, and let *r*_*E*_ and *r*_*A*_ be the growth rates of the two strains. Then, $\frac{E(t)}{A(t)}=\frac{E(0)}{A(0)}\cdot e^{(r_{E}-r_{A})t}$, and we have $\ln\left(\frac{E(t)}{A(t)}\right)=\ln\left(\frac{E(0)}{A(0)}\right)+(r_{E}-r_{A})t$. We quantified (*r*_*E*_−*r*_*A*_), the fitness advantage of *ecm21* over ancestor, as 0.31/h (computed up to 32 h), compared with the ancestor growth rate of 0.087/h (8-h doubling). Thus, the growth rate of *ecm21* is about 3.6-fold that of the ancestor in 8-h doubling chemostats. This fitness advantage is qualitatively consistent with what we observed in chemostats initiated with pure ancestor, because evolved clones increased from about 4% to about 40% within 5.7 h (from 26.3 to 32 h in Fig 3C), translating to a 0.49/h fitness difference. We infer that evolved clones are initially present at a frequency on the order of about 0.04/exp(0.4/h * 26.3 h) = 10^−6^. This is in line with the phenotypic mutation rate of 0.5–30 × 10^−7^ per cell per generation [90] in the following sense. Because the inoculum population size is on the order of 4 × 10^6^ cells/mL × 19 mL = 8 × 10^7^ cells, we expect 2 × 8 × 10^7^ * (0.5–30 × 10^−7^) = 8–480 pre-existing evolved cells (the coefficient of 2 results from (1+2+2^2^+⋯+2^*n*^)/2^*n*^~2, where the numerator represents the total mutation opportunity in a culture starting from a single cell, and the denominator represents the final size of chemostat inoculum). Thus, the early evolutionary dynamics in chemostats can be explained by pre-existing mutants outcompeting ancestral cells. (**B**) Percentage evolved clones in 26-h samples from chemostats inoculated with ancestral *L*^*−*^*A*^***+***^ (WY1335). (**C**) A visual assay that distinguishes ancestral versus evolved *L*^*−*^*A*^***+***^ clones. *L*^*−*^*A*^***+***^ cells from an ancestral clone and two evolved clones were plated on SD plates supplemented with 1.5 μM lysine. Ancestral cells (WY1335, left) failed to divide (arrows). Cells from a mildly adapted evolved clone (harboring duplication of Chromosome 14, center) showed heterogeneous phenotypes: some cells remained undivided (arrow), while other cells formed microcolonies of various sizes. Cells from a strongly adapted evolved clone (harboring an *ecm21* mutation, right) formed microcolonies of a uniform and large size. These images were taken using a cellular phone camera and thus do not have a scale bar. For reference, an average yeast cell has a diameter of about 5 μm. All data can be found in S13 Data. Lyp1, high-affinity lysine permease; Ecm21 and Rsp5, proteins involved in degrading Lyp1; SD, Synthetic Dextrose minimal medium.(TIF)

S8 Fig Characterization of evolved *A*^*−*^*L*^*+*^ clones. (**A**) A fitness trade-off in *A*^*−*^*L*^***+***^. (**Left**) Growth rates of ancestral (magenta) and evolved (blue and green) *A*^*−*^*L*^***+***^ clones (prestarved overnight) in various concentrations of hypoxanthine (Methods, “Microscopy quantification of growth parameters”) were plotted. Brown dotted lines mark 6-h and 8-h doubling time, a range experienced by CoSMO. In CoSMO, hypoxanthine concentrations were low (about 1 μM). Evolved clones grew faster than the ancestor under low hypoxanthine concentrations, but grew slower than the ancestor under high hypoxanthine concentrations (e.g., 20–100 μM). Clones marked by crosses were isolated from Generation 4 (hour 31) of chemostat culturing. (**Right**) A negative correlation between growth rate at low hypoxanthine versus turbidity in high adenine after overnight growth. Error bars on growth rate indicate the 95% confidence interval on slope (rate) estimation. Gray line indicates the threshold by which we differentiated evolved clones (left of gray line) from ancestral clones (right of gray line), according to the growth rate assay in the left panel. In both panels, gray crosses represent Generation 4 clones assigned to be evolved, while black crosses represent Generation 4 clones assigned to be ancestral. (**B**) A high-throughput assay that distinguishes ancestral from evolved *A*^*−*^*L*^***+***^ clones. We used turbidity after overnight growth in high Ade (108 μM) to classify *A*^*−*^*L*^***+***^ clones as ancestral (no blue stars) or evolved (blue stars). The ancestral clone (WY1340) and an evolved clone (WY1598) are shown as controls. (**C**) Aneuploidy in the evolved clone WY2447. Whole-genome sequencing revealed that, in addition to a synonymous nucleotide change, two nucleotide changes in noncoding regions and a point mutation from Cys102 to Ser in the gene *OAR1* (S3 Table), Chromosomes I, III, and VI are likely duplicated. For Chromosome III, read depth was not fully twice that of other chromosomes, which could be caused by cells losing the extra copy of Chromosome III during culturing prior to sequencing. (**D**) Evolved *A*^*−*^*L*^***+***^ cells have a lower death rate, a lower lysine release rate, and lower hypoxanthine consumption per birth compared with the ancestor. Phenotypes of ancestral *A*^*−*^*L*^***+***^ (WY1340 with pre-existing WY2447-like mutants), 1:1 anc:evo (WY1340:WY2447), or evolved *A*^*−*^*L*^***+***^ (WY2447) were measured in 8-h chemostats (Methods, “Quantifying phenotypes in chemostats”). All data can be found in S14 Data. Ade, adenine; anc, ancestor; CoSMO, Cooperation that is Synthetic and Mutually Obligatory; Cys, cysteine; evo, evolved; Hyp, hypoxanthine; Ser, serine.
(TIF)

S9 FigHigh levels of evolved clones in *A*^*−*^*L*^*+*^.(**A**) High levels of evolved *A*^*−*^*L*^***+***^ clones prior to a starvation experiment (left of dotted line) and during a starvation experiment (right of dotted line). Different colors represent experiments on different days. In experiments that terminated at 0 h (circles and squares), an entire colony grown on rich YPD (circles) or minimal SD plus excess (100 μM) hypoxanthine (square) (-20 h) was resuspended in SD, and a fraction was used to inoculate SD plus excess hypoxanthine to grow exponential cultures (0 h). Otherwise (triangles), a fraction of the YPD-grown colony was used to inoculate SD plus excess hypoxanthine to grow exponential cultures, and at time zero, the culture was washed free of supplements and starved of hypoxanthine. (**B**) Similar percentages of evolved *A*^*−*^*L*^***+***^ clones in chemostats and in CoSMO. For chemostat experiments, exponentially growing cells washed free of supplements were prestarved (unfilled symbols) or not prestarved (filled symbols), and inoculated into chemostats (time zero). For CoSMO experiments, *A*^*−*^*L*^***+***^ cells were prestarved. In all experiments, we used the assay in S8B Fig to distinguish ancestral and evolved clones (Methods, “Detecting evolved clones”). If we sampled *n*_*tot*_ cells, and *n*_*evo*_ cells were evolved, then the fraction evolved was estimated to be *n*_*evo*_/*n*_*tot*_, with the error bar indicating $\frac{2\sqrt{n_{evo}}}{n_{tot}}$ (assuming that the random variable *n*_*evo*_ followed a Poisson distribution). If zero assayed colonies were evolved, we identified the maximal frequency of evolved clones such that the error bar of $\frac{2\sqrt{n_{evo}}}{n_{tot}}$ still covered zero, and used that error bar. For example, if 0 out of 88 were evolved (0%), and because 3 out of 88 had a frequency of 3.4%, with an error bar of 3.9%, which covered zero, we added an error bar of 3.9% above the 0% data point. All data can be found in S15 Data. CoSMO, Cooperation that is Synthetic and Mutually Obligatory; SD, Synthetic Dextrose minimal medium; YPD, Yeast extract Peptone Dextrose rich medium.(TIF)

S10 Fig Evolutionary dynamics of *A*^*−*^*L*^*+*^ are consistent with high chromosomal mis-segregation rate. (**A**) Growth rates of ancestral (WY1340) and evolved (WY2447) *A*^*−*^*L*^***+***^ cells in various concentrations of hypoxanthine (24-h prestarvation; Methods, “Microscopy quantification of growth phenotypes”). Three experiments were averaged, and error bars indicate 2 standard deviations. (**B**) The evolutionary dynamics of *A*^*−*^*L*^***+***^ in excess hypoxanthine could be explained if we assumed that chromosome mis-segregation generated WY2447-like mutants at a rate of 0.01/cell/generation (solid lines). As a comparison, predictions from a mutation rate of 0.003/cell/generation (dotted lines) were also plotted. Brown and blue circles (measured in two different experiments) are identical to the corresponding ones in S9A Fig. Specifically, from the inoculum size and the final population size, we calculated the number of generations, which we then multiplied with the doubling time in SD with excess hypoxanthine to obtain the duration of the exponential phase. We then inferred the lag phase to be about 6 h and assumed that the fraction of evolved cells at time zero (the beginning of exponential phase) was similar to that at the time of inoculation. Our model (S2 Code) considered the fitness advantage of ancestor over mutant in excess hypoxanthine (**A**), as well as the conversion from ancestor to mutant. Data at 0 h were slightly jittered to aid visualization. (**C**) We competed WY2447 (expressing citrine fluorescent protein) and WY1340 (expressing green fluorescent protein) in 8-h chemostats from two starting ratios, and measured strain ratios over time using flow cytometry (black circles). Using a mathematical model (S3 Code) in which growth parameters were measured experimentally (**A**) and the ancestor converted to WY2447-like mutants at a rate of 0.01/cell/generation, we obtained a qualitative matching between model and experiments. In both models (**B**, **C**), death rate and hypoxanthine consumption per birth were from 8-h chemostat measurements (Methods, “Quantifying phenotypes in chemostats”). All data can be found in S16 Data. SD, Synthetic Dextrose minimal medium.
(TIF)

S11 Fig Measured *A*^*−*^*L*^*+*^ phenotypes are not significantly impacted by percentage evolved clones during quantification. *A*^*−*^*L*^***+***^ (WY1340), either prestarved for 24 h (open circles) or not prestarved (filled circles), were cultured in 8-h chemostats (different colors representing independent chemostat experiments). Hypoxanthine consumption per birth (**A**), death rate (**B**), and lysine release rate (**C**) were quantified (Methods, “Quantifying phenotypes in chemostats”) using dynamics up to 48–50 h (small-sized circles), 67–72 h (medium-sized circles), or 94–96 h (large-sized circles). Percentage of evolved clones was quantified at the end of each measurement. Despite phenotypic differences between ancestral and evolved *A*^*−*^*L*^***+***^ (S8D Fig), measured phenotypes did not show significant correlation with percentage evolved (slope ± standard error of the mean [SEM] being 0.1 ± 0.8 (**A**), 0.003 ± 0.008 (**B**), and 0.3 ± 0.6 (**C**)—none significantly different from zero). This lack of correlation is presumably due to the relatively large measurement errors and the relatively narrow spread in percentage evolved. Take consumption as an example. Suppose that ancestral and evolved *A*^*−*^*L*^***+***^ consumed hypoxanthine at 2.5 fmole/birth and 1.5 fmole/birth, respectively (S8D Fig). At 10% mutants, consumption would be 2.5 * 0.9 + 1.5 * 0.1 = 2.4 fmole/birth. At 30% mutants, consumption would be 2.5 * 0.7 + 1.5 * 0.3 = 2.2 fmole/cell. This 10% difference is smaller than the measurement error. For example, at about 33% evolved *A*^*−*^*L*^***+***^ (filled dots in **A**), consumption varied from 2.2 to 2.8 fmole/birth. In summary, quantified phenotypes did not correlate strongly with percentage mutants because percentage mutants were sufficiently similar across different replicates and because measurement errors were sufficiently large. All data can be found in S17 Data.
(TIF)

S12 Fig Parameters measured from *L*^*−*^*A*^*+*^ chemostats recapitulate chemostat dynamics. *L*^*−*^*A*^***+***^ cells in SD + 15 μM lysine were inoculated into a chemostat culturing vessel (19 mL). SD + 20 μM lysine in the reservoir was pumped into the culturing vessel to achieve an 8-h doubling time (i.e., 19 mL * ln(2)/8/h = 1.646 mL/h). *L*^*−*^*A*^***+***^ phenotypes in Table 1 (except for release rate of 0.30 fmole hypoxanthine/cell/h and death rate of 0.0021/h measured in this particular experiment) were used to simulate chemostat dynamics (S4 Code). Simulations (lines) and experiments (circles) are in good agreement. All data can be found in S18 Data. SD, Synthetic Dextrose minimal medium.
(TIF)

S13 Fig Purine consumption by *A*^*−*^*L*^*+*^ is relatively constant during purine limitation. For hypoxanthine-limited chemostat measurements, data were jittered slightly along the *x* axis to facilitate visualization. Consumption was measured over a similar time window as that of CoSMO growth rate to ensure similar evolutionary effects. For exponential and saturation consumption of adenine (which is similar to hypoxanthine, see S3A Fig), error bars mark 2 SEMs for slope estimation. The black dashed line marks the average hypoxanthine consumption per *A*^*−*^*L*^***+***^ birth in chemostats (Table 1; data can be found in S6 Table). CoSMO, Cooperation that is Synthetic and Mutually Obligatory; SEM, standard error of the mean.
(TIF)

S14 Fig Regression analysis reveals death and release rates in a chemostat. We used regression to measure death rate (gray) and hypoxanthine release rate (blue) for the triangle-marked chemostat experiment from Fig 3. For an explanation, see “Quantifying phenotypes in chemostats” in Methods. Densities of fluorescent live cells and nonfluorescent/ToPro3-positive dead cells were measured via flow cytometry (Methods, “Flow cytometry”). Hypoxanthine was quantified using the yield bioassay (Methods, “Bioassays”). All data can be found in S19 Data.
(TIF)

S15 Fig Quantifying release and death rates of *A*^*−*^*L*^*+*^. *A*^*−*^*L*^***+***^ cells grown to exponential phase in SD + excess hypoxanthine were washed and diluted into SD. (**A**-**C**) “Starvation”: at time zero, cells were inoculated into SD. From population and lysine dynamics (**A**), the live release model (**B**) and the dead release model (**C**) yielded similar fits to the data. Thus, from regression alone, we could not distinguish live from dead release. (**D-F**) “Chemostat”: cells were prestarved in SD for 24 h and then transferred to a hypoxanthine-limited chemostat (doubling time, 8 h) at time zero. From population and lysine dynamics (**D**), lysine release rate by live cells (**E**) and death rate (**F**) can be calculated from slopes of respective regressions (Methods, “Quantifying phenotypes in chemostats”). Note that lysine release rate during starvation (**B**) remained relatively constant during the initial 90 h (excluding the last two data points; the time window we later used to measure CoSMO growth rate; also see S18B Fig). In chemostat, upon reaching the steady state, the release rate also remained relatively constant (the last three time points in **D, E**). All data can be found in S20 Data. CoSMO, Cooperation that is Synthetic and Mutually Obligatory; SD, Synthetic Dextrose minimal medium.
(TIF)

S16 Fig *A*^*−*^*L*^*+*^ intracellular lysine content varies with time and environment. *A*^*−*^*L*^***+***^ cells (WY1340) grown in SD + excess hypoxanthine to exponential phase were washed and diluted into SD at time zero. Cells were either starved further (**B**) or inoculated into hypoxanthine-limited chemostats after 24 h of prestarvation (**A**). At various times, cells were harvested, and intracellular lysine was extracted and measured via yield bioassay. Different colors (except gray) represent different replicates and are identical to those in Fig 6A. Gray symbols in (**A**) represent the intracellular lysine content required to satisfy the dead release model at the steady state (calculated from the last three time points of S15D Fig). Gray symbols and dashed lines in (**B**) represent the intracellular lysine content required to satisfy the dead release model during starvation. Because live or dead release could explain chemostat results (**A**) but dead release could not explain starvation results (**B**), we made the most parsimonious assumption of live release. All data can be found in S21 Data. SD, Synthetic Dextrose minimal medium.
(TIF)

S17 Fig Slight cell size increase during *A*^*−*^*L*^*+*^ starvation. *A*^*−*^*L*^***+***^ cells (WY1340) grown in SD + excess hypoxanthine were either maintained at exponential phase (green) or washed and starved in unsupplemented SD for 24 h (black). Cell sizes were measured using a Coulter counter. The initial peak in starved cells may represent dead cell debris. The average sizes of exponential and starved cells were 64.8 fL and 80.5 fL, respectively (dashed lines). All data can be found in S22 Data. SD, Synthetic Dextrose minimal medium.
(TIF)

S18 Fig Lysine release by *A*^*−*^*L*^*+*^ during starvation is relatively constant during the initial 90 h. (**A**) Exponentially growing *A*^*−*^*L*^***+***^ cells were washed and diluted into SD. Live and dead population densities were measured by flow cytometry, and lysine concentration was measured by the yield bioassay. Regression in both the live release model (**B**) and dead release model (**C**) deviated from linearity. Because metabolite analysis suggests that live release is more likely (S16B Fig), we infer that the release rate is time variant—initially slow and then speeding up (a similar but less obvious trend can also be seen in S15B Fig). However, because CoSMO growth rate measurements rarely exceeded 96 h, we used the lysine release rate measured up to 90 h in Model ii. All data can be found in S23 Data. CoSMO, Cooperation that is Synthetic and Mutually Obligatory; SD, Synthetic Dextrose minimal medium.
(TIF)

S19 Fig The *A*^*−*^*L*^*+*^ chemostat model can describe experimentally observed chemostat steady state. *A*^*−*^*L*^***+***^ cells grown exponentially in SD + excess hypoxanthine were washed and diluted into SD, and prestarved for 24 h. At time zero, starved cells (together with the medium, which had already accumulated some lysine) were inoculated into chemostats, and fresh SD + 20 μM hypoxanthine was pumped in at a rate to achieve a doubling time of 8 h. Dynamics of live and dead populations (left) and of released lysine (right) were plotted (squares, circles, and diamonds representing three chemostats). The model (dashed lines; S5 Code) was based on parameters in Table 1, except for a lysine release rate of 0.99 fmole/cell/h, which was averaged among the three chemostats. The initial decline in live cell density in experiments was presumably due to a growth lag when cells transitioned from starvation to chemostats, which was not modeled. The initial decline in extracellular lysine concentration in experiments is consistent with the live release model: reduced live cell density leads to reduced extracellular lysine. All data can be found in S24 Data. SD, Synthetic Dextrose minimal medium.
(TIF)

S20 Fig Measuring lysine release rate. *A*^*−*^*L*^***+***^ cells grown to exponential phase in SD + excess hypoxanthine were washed and diluted into SD at time zero. Cells were either starved further (“Starve”) or inoculated into hypoxanthine-limited chemostats after 24 h of prestarvation (e.g., S15D and S15E Fig; doubling times marked above). Each symbol represents an independent measurement, and measurements done at the same time were marked with the same color. Open and closed symbols represent pregrowth done in tubes versus flasks, respectively. We observed day-to-day variations in measurements (e.g., cyan circles higher than blue triangles). Lysine release rate data are in S8 Table. SD, Synthetic Dextrose minimal medium.
(TIF)

S21 Fig Death rates of *L*^*−*^*A*^*+*^ and *A*^*−*^*L*^*+*^ are relatively constant over a range of CoSMO-like environments. (**A**) Exponential *L*^*−*^*A*^***+***^ (WY1335) cells were washed in SD, and death rate was measured in chemostats at various doubling times (Methods, “Quantifying phenotypes in chemostats,” Eq 16; S14 Fig, gray). As a comparison, death rates in batch cultures with zero or excess lysine are shown (see [27] for detailed methodology and data). With no lysine, the early-phase death rate (gray; from 5 to 12 h post-wash) was slower than the late-phase death rate (black; from 12 to 30 h post-wash). With excess lysine, death rates were very low (orange diamonds; Methods, “Calculating death rate in nonlimited batch culture”). Death rates of *L*^*−*^*A*^***+***^ in chemostats (blue; doubling times from left to right being 8, 6, 5.5, and 3 h) were in between death rates in starvation and in excess lysine. Blue solid and dashed lines mark the mean death rate ±2 SEMs from 5.5–8-h doubling time chemostats (Table 1). Detailed data for **A** are in S7 Table. We used a log plotting scale to visualize differences between small numbers. (**B**) Exponential *A*^*−*^*L*^***+***^ (WY1340) cells were washed and prestarved for 24 h. They were either further starved (black and gray) or cultured at various growth rates in chemostats (blue). During starvation, death rate was initially slow (gray) and then sped up (black; see [27] for detailed methodology and data). Average death rate (blue solid line) and 2 SEM (blue dotted lines) were calculated from chemostats run at doubling times of 5.4–8 h (e.g., S15F Fig) and used in our model (Table 1). We used a linear plotting scale because early death rates were zero and could not be plotted on the log scale. In both **A** and **B**, death rates were quantified from the decline rate of ln(live population size), and live population size could be measured via microscopy total fluorescence intensity [27] (circles), microscopy live cell count [27] (triangles), or flow cytometry live cell density (diamonds; Methods, “Flow cytometry”). Detailed data for **B** are in S8 Table. CoSMO, Cooperation that is Synthetic and Mutually Obligatory; SD, Synthetic Dextrose minimal medium; SEM, standard error of the mean.
(TIF)

S22 FigA spatially structured environment slows down *L*^*−*^*A*^*+*^ evolution in CoSMO.**(A)** *L*^*−*^*A*^***+***^ evolves rapidly in a well-mixed environment. Exponentially growing *L*^*−*^*A*^***+***^ (WY1335) and *A*^*−*^*L*^***+***^ (WY1340) were washed free of supplements, preconditioned, and mixed at 1:1 in SD at a total cell density of 10^5^/mL. The resultant CoSMO was grown in a well-mixed environment. At various times, samples were plated on YPD, and 32 *L*^*−*^*A*^***+***^ colonies (red diamonds) and 84–96 *A*^*−*^*L*^***+***^ colonies (green circles) were isolated to assay whether they were evolved or not (Methods, “Detecting evolved clones”). (**B**) *L*^*−*^*A*^***+***^ evolves slowly in a spatially structured environment. *L*^*−*^*A*^***+***^ (WY1335 and WY1657) and *A*^*−*^*L*^***+***^ (WY1340 and WY1342) were mixed at approximately equal ratio and spotted onto the middle of an agarose slice containing 0.7 μM lysine (the spotting setting in Methods, “Quantifying spatial CoSMO growth dynamics”; prestarved *A*^*−*^*L*^***+***^ cells were washed again in SD so that CoSMO started with a defined level of lysine in the agarose pad). At 96 h, CoSMO samples were plated on YPD, and 80 *L*^*−*^*A*^***+***^ colonies (red diamonds) and 88 *A*^*−*^*L*^***+***^ colonies (green circles) were isolated to assay whether they were evolved or not. For (**A**) and (**B**), error bars indicate 2 standard deviations according to binomial distribution. Specifically, if we observed *e* evolved clones among *N* total clones, then the fraction evolved was *p* = *e/N* and the error bar was $2[\sqrt{Np(1-p)}]/N$. If no evolved clones were observed, then *p* = 0, and the upper error bar was defined to be that of maximal *e*, whose lower error bar spanned 0 (similar to S9 Fig). Error bars were truncated at 0 and 1. All data can be found in S25 Data. CoSMO, Cooperation that is Synthetic and Mutually Obligatory; SD, Synthetic Dextrose minimal medium; YPD, Yeast extract Peptone Dextrose rich medium.(TIF)

S23 Fig Predicting the steady-state growth rate of spatial CoSMO. Spatial CoSMO growth was simulated under varying initial total cell density, inoculation setup (uniformly plated “u” versus centrally spotted “s”), agar depth, and diffusion coefficient (20 and 360 μm^2^/s, corresponding to diffusion coefficients in community and agarose, respectively [32]). Spatial simulations yielded similar CoSMO growth rates (brown). Experimental measurements of spatial CoSMO (purple) and CoSMO growth rate calculated from Eq 17 (orange) were taken from Fig 7 and plotted here for comparison. The spatial model and the calculation both considered variable lysine release rate. All data can be found in S26 Data. CoSMO, Cooperation that is Synthetic and Mutually Obligatory.
(TIF)

S24 Fig Population dynamics of spatial CoSMO. Preconditioned *L*^*−*^*A*^***+***^ and *A*^*−*^*L*^***+***^ were mixed at approximately 1:1 ratio and grown on 2 × SD agar (which may contain trace nutrient contaminants) or agarose (Methods, “Quantifying spatial CoSMO growth dynamics”). (**A**) Growth dynamics of CoSMO on four media. Inset: shared experimental setup. A total of 15 μL of 4 × 10^4^ total cells was spotted on the center of the cut pad, forming an inoculum spot of radius 4 mm. The lower left panel is identical to Fig 7A. (**B**) Growth dynamics of CoSMO in deep 96-well plates. A total of 1.5 × 10^5^ initial total cells were filtered on top of a membrane filter to ensure uniform spatial distribution. This was equivalent to 3,000 cells/mm^2^. (**C**) After the lag phase, steady-state growth rates of CoSMO were calculated from 11 independent experiments, with color-coding corresponding to conditions in (**A**) and (**B**). Time points at which total cell numbers exceed 1 × 10^8^ were excluded to avoid stationary phase. Error bars mark 2 standard errors of estimating growth rate. In **A** and **B**, each data point represented the average of three flow cytometry measurements of a single spatial sample. Experimental data for **A** and **B** and summary data for **C** are provided in S4 Table. CoSMO, Cooperation that is Synthetic and Mutually Obligatory; SD, Synthetic Dextrose minimal medium.
(TIF)

S25 Fig Spatial CoSMO eventually resembles well-mixed CoSMO. Metabolite concentrations in the agarose and the community eventually reach a nearly uniform state. Plotted are top views of lysine (*L*, left panel) and hypoxanthine (*A*, right panel) concentrations in the cell layer immediately on top of the agarose surface 120 h after being spotted in the middle of an agarose pad. Because the populations were fairly intermixed within the community [32], the overall metabolite distributions remained fairly uniform within the community. The spatial averages of *L* and *A* in the community were 1.35 μM and 0.79 μM, respectively. The average concentrations in the agarose (1.31 μM for *L* and 0.73 μM for *A*) closely matched those inside the community. Thus, the CoSMO growth rate in a spatially structured environment is similar to that in a well-mixed environment. Here, the diffusion coefficients inside CoSMO and agarose were 20 and 360 μm^2^/s, respectively. CoSMO, Cooperation that is Synthetic and Mutually Obligatory.
(TIF)

S26 Fig Model iii does not quantitatively capture detailed dynamics during and immediately after the lag phase. (**A**) Predicted (dotted lines; S8 Code) and measured (symbols; identical to those in Fig 1B) dynamics of a well-mixed community. (**B**) Predicted (dotted lines; S7 Code) and measured (symbols; identical to those in Fig 7A) dynamics of a community grown on top of an agarose pad. In **B**, the growth slowdown toward the end of simulations is due to exhaustion of shared nutrients in the agarose pad. All parameters are from Table 1.
(TIF)

S1 Table Strain table. (XLSX)

S2 Table Parameters for models i and ii, corresponding to Fig 1 and S1 Fig. CoSMO, Cooperation that is Synthetic and Mutually Obligatory.
(XLSX)

S3 Table Mutations in WY2447. (XLSX)

S4 Table Experimental measurements of spatial CoSMO population dynamics and a summary of steady-state CoSMO growth rate. CoSMO, Cooperation that is Synthetic and Mutually Obligatory.
(XLSX)

S5 Table *L*^*−*^*A*^*+*^ consumption. (XLSX)

S6 Table *A*^*−*^*L*^*+*^ consumption. (XLSX)

S7 Table *L*^*−*^*A*^*+*^ death and release rates. (XLSX)

S8 Table *A*^*−*^*L*^*+*^ death and release rates. (XLSX)

S1 Code Initial models for well-mixed CoSMO corresponding to Fig 1. CoSMO, Cooperation that is Synthetic and Mutually Obligatory.
(ZIP)

S2 Code Modeling *A*^*−*^*L*^*+*^ evolution in excess hypoxanthine. (ZIP)

S3 Code Modeling competition between evolved and ancestral *A*^*−*^*L*^*+*^ strains in hypoxanthine-limited chemostats. (ZIP)

S4 Code Modeling chemostat dynamics of *L*^*−*^*A*^*+*^. (ZIP)

S5 Code Modeling chemostat dynamics of *A*^*−*^*L*^*+*^. (ZIP)

S6 Code Spatial CoSMO model with a similar diffusion coefficient for agar and community regions. CoSMO, Cooperation that is Synthetic and Mutually Obligatory.
(ZIP)

S7 Code Spatial CoSMO model with separate diffusion coefficients for agar and community regions. CoSMO, Cooperation that is Synthetic and Mutually Obligatory.
(ZIP)

S8 Code Well-mixed CoSMO model incorporating variable lysine release rate. CoSMO, Cooperation that is Synthetic and Mutually Obligatory.
(ZIP)

S1 Data Additional well-mixed CoSMO experimental data. CoSMO, Cooperation that is Synthetic and Mutually Obligatory.
(XLSX)

S2 Data Data plotted in Fig 2. (XLSX)

S3 Data Data plotted in Fig 3. (XLSX)

S4 Data Data plotted in Fig 4. (XLSX)

S5 Data Data plotted in Fig 5A and 5B. (XLSX)

S6 Data Data plotted in Fig 6A. (XLSX)

S7 Data Data plotted in Fig 7. (XLSX)

S8 Data Data plotted in S2 Fig. (XLSX)

S9 Data Data plotted in S3 Fig. (XLSX)

S10 Data Data plotted in S4 Fig. (XLSX)

S11 Data Data plotted in S5 Fig. (XLSX)

S12 Data Data plotted in S6 Fig. (XLSX)

S13 Data Data plotted in S7 Fig. (XLSX)

S14 Data Data plotted in S8 Fig. (XLSX)

S15 Data Data plotted in S9 Fig. (XLSX)

S16 Data Data plotted in S10 Fig. (XLSX)

S17 Data Data plotted in S11 Fig. (XLSX)

S18 Data Data plotted in S12 Fig. (XLSX)

S19 Data Data plotted in S14 Fig. (XLSX)

S20 Data Data plotted in S15 Fig. (XLSX)

S21 Data Data plotted in S16 Fig. (XLSX)

S22 Data Data plotted in S17 Fig. (XLSX)

S23 Data Data plotted in S18 Fig. (XLSX)

S24 Data Data plotted in S19 Fig. (XLSX)

S25 Data Data plotted in S22 Fig. (XLSX)

S26 Data Data plotted in S23 Fig. (XLSX)

## Abbreviations

CoSMO: Cooperation that is Synthetic and Mutually Obligatory
HPLC: high-pressure liquid chromatography
nanoDESI MS: nanospray desorption electrospray ionization mass spectrometry imaging
SEM: standard error of the mean
UHPLC: ultra-performance HPLC
