# Supplementary figures and images for: Uncovering and resolving challenges of quantitative modeling in a simplified community of interacting cells
Source: PLoS Biol. 2019 Feb 22;17(2):e3000135. doi: 10.1371/journal.pbio.3000135 (PMC6402699; doi:10.1371/journal.pbio.3000135)

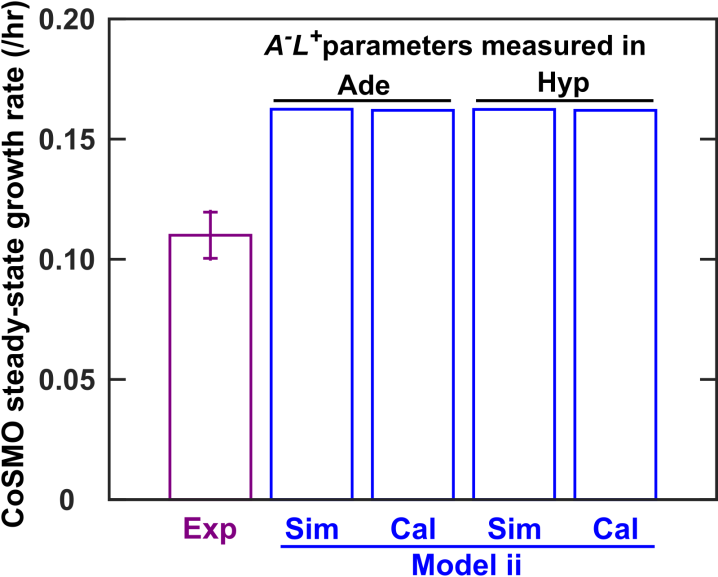

Supplement: S1 Fig — “Exp”: community growth rates were calculated from seven independent experiments in a well-mixed environment (from about 30 h to 70–80 h) and averaged, with the error bar representing 2 standard deviations. “Model ii”: all model parameters were derived from L−A+ and A−L+ of the RM11 genetic background measured in batch monocultures. We predicted steady-state community growth rate either via quantifying the simulated post-lag dynamics (e.g., Fig 1B “Model ii”) (“Sim”) or via an analytical formula (Eq 17 in Methods) (“Cal”). The experimental and predicted doubling times were 6.5 and 4.3 h, respectively. Experimental data and model parameters are listed in S1 Data and S2 Table, respectively. Cal, calculation; Exp, experiment; Sim, simulation. (TIF) [file pbio.3000135.s001.tif]

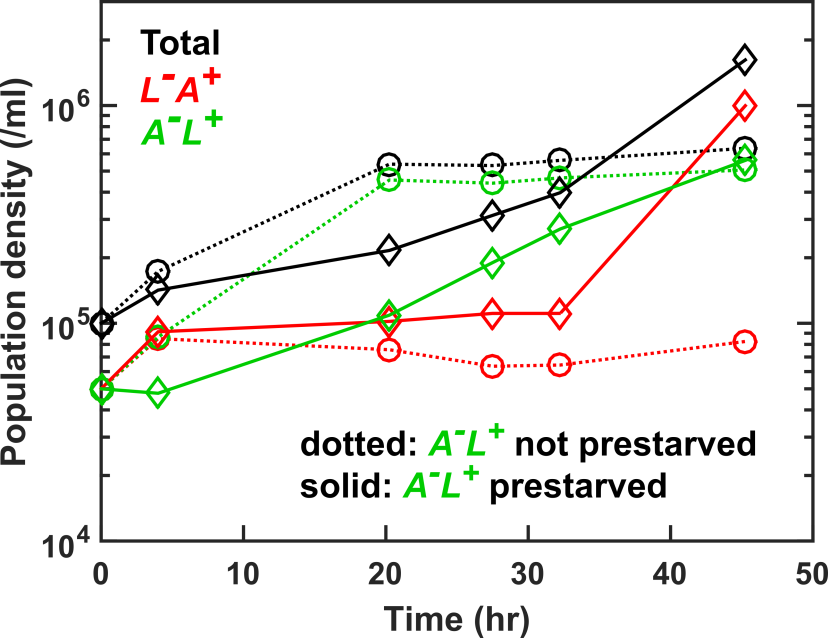

Supplement: S2 Fig — Exponential A−L+ (WY1340) cells were washed free of hypoxanthine and either prestarved for 24 h in SD (solid lines) or not prestarved (dotted lines) before being mixed with exponentially grown and then washed L−A+ (WY1335) to form CoSMO. Prestarvation of A−L+ leads to less growth lag compared with no prestarvation. All data can be found in S8 Data. CoSMO, Cooperation that is Synthetic and Mutually Obligatory. (TIF) [file pbio.3000135.s002.tif]

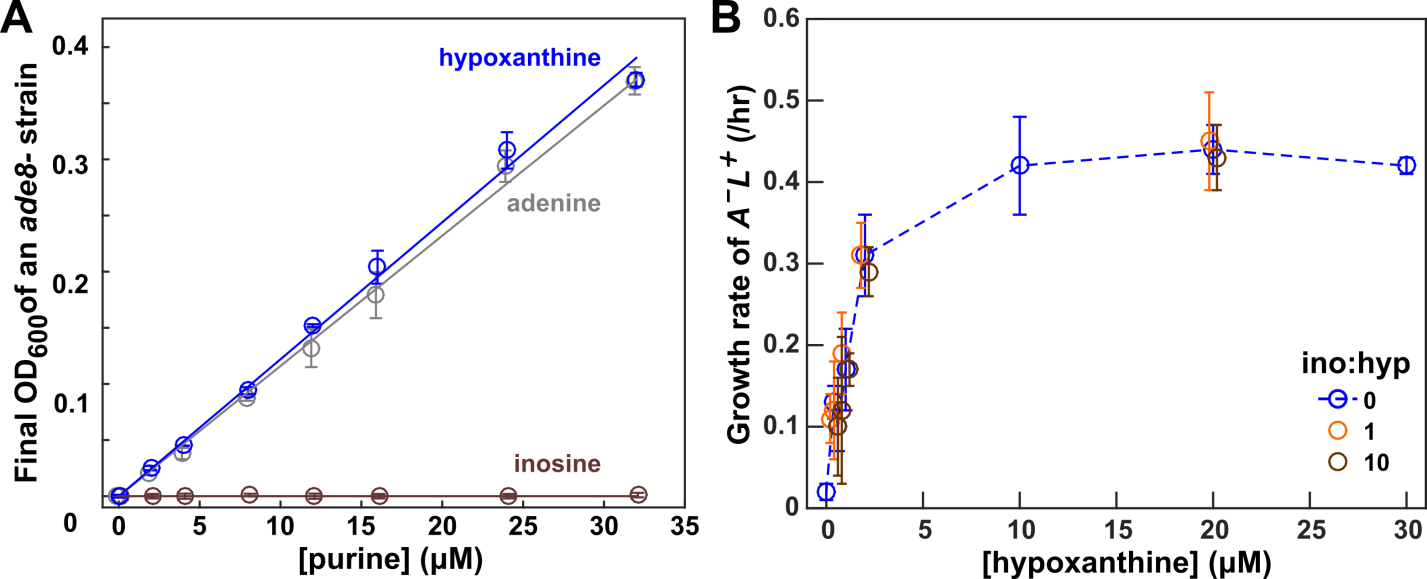

Supplement: S3 Fig — (A) Hypoxanthine but not inosine is consumed by A−L+. The final turbidity of an ade8− (WY1340) tester strain increases with increasing concentrations of hypoxanthine (blue) and adenine (gray), but not inosine (brown). The slopes of the blue and gray lines are similar, suggesting that a similar amount of hypoxanthine and adenine are consumed to produce one new A−L+ cell. (B) Stimulation of the A−L+ (WY1340) growth rate by hypoxanthine (blue) is not affected by the presence of inosine at 1× (orange) or 10× (brown) concentration. All data can be found in S9 Data. (TIF) [file pbio.3000135.s003.tif]

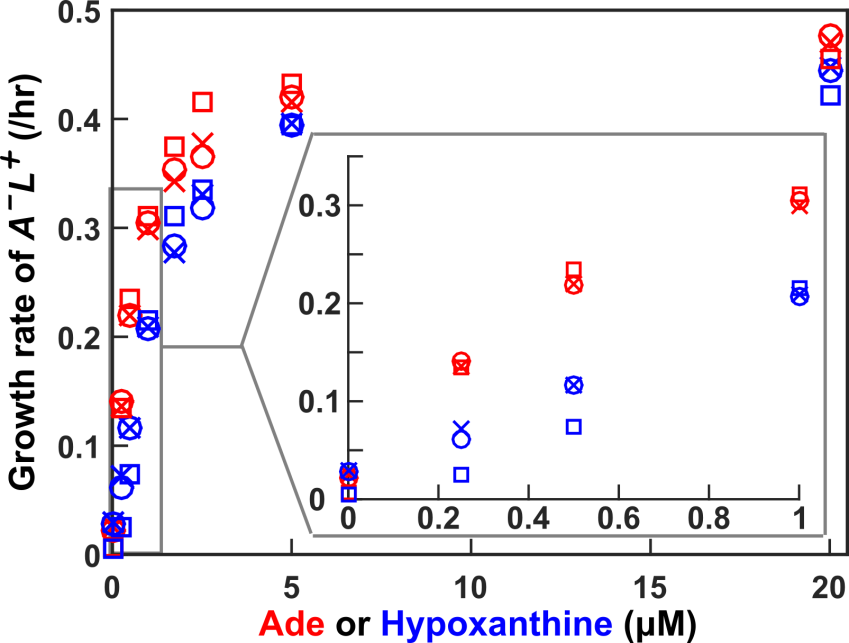

Supplement: S4 Fig — A−L+ cells grow faster when fed with adenine (red) than when fed with hypoxanthine (blue) when metabolite concentration is low (inset). A−L+ (WY1340) cells pregrown in SD + adenine or SD + hypoxanthine were washed into SD and prestarved for 24 h to deplete intracellular storage. Subsequently, adenine or hypoxanthine was supplemented at various concentrations, and the net growth rate was measured via fluorescence microscopy (Methods, “Microscopy quantification of growth parameters”). Red circles and squares: pregrown in adenine and incubated in adenine; red crosses: pregrown in hypoxanthine and incubated in adenine; blue circles and squares: pregrown in hypoxanthine and incubated in hypoxanthine; blue crosses: pregrown in adenine and incubated in hypoxanthine. Pregrowth in cognate metabolite versus noncognate metabolite does not make a difference (e.g., compare red circles with red crosses and blue circles with blue crosses, all of which were measured in the same experiment). All data can be found in S10 Data. (TIF) [file pbio.3000135.s004.tif]

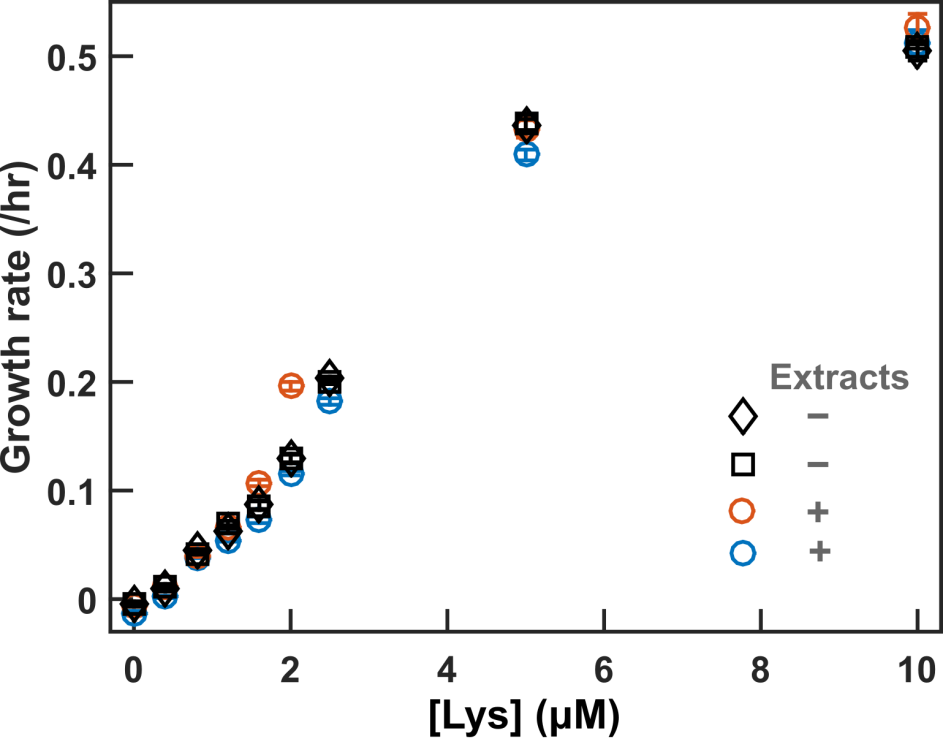

Supplement: S5 Fig — Exponential L−A+ (WY1335) cells were starved in SD for 4 h to deplete intracellular storage of lysine. A total of 2.5 mL of starved culture at OD 0.2 was used to extract intracellular metabolites (“Extraction of intracellular metabolites” in Methods). The dried pellet was resuspended in about 1 mL H2O. In a separate experiment, exponential L−A+ were washed and prestarved in SD for 4 h. We then quantified the growth rates of L−A+ in SD supplemented with 1/3 volume of extracts (orange and blue) or water (black), as well as various concentrations of lysine (“Microscopy quantification of growth phenotypes” in Methods). The inclusion of extracts did not affect growth rates. All data can be found in S11 Data. OD, optical density at 600 nm; SD, Synthetic Dextrose minimal medium. (TIF) [file pbio.3000135.s005.tif]

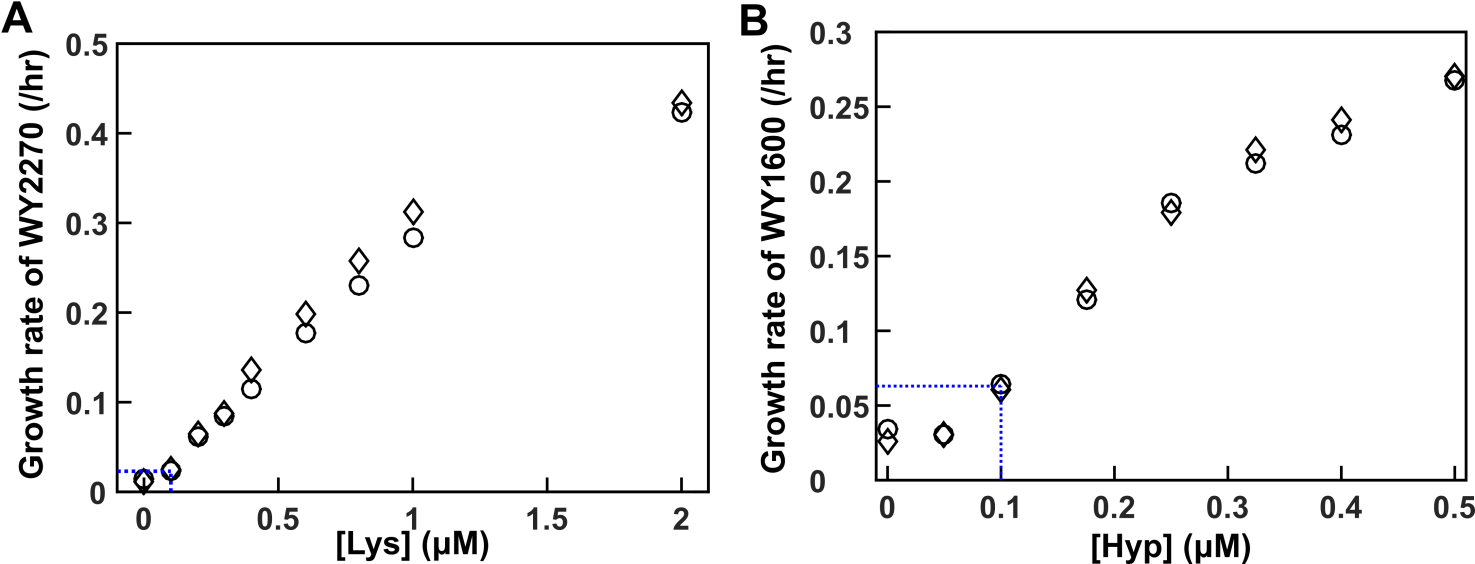

Supplement: S6 Fig — (A) WY2270, an evolved L−A+ clone with significantly improved affinity for lysine, could detect sub–1 μM Lys. (B) WY1600, an evolved A−L+ clone with a significantly improved affinity for hypoxanthine, could detect sub–1 μM hypoxanthine. Vertical dotted blue lines mark detection limits. Circles and diamonds mark two independent replicates. All data can be found in S12 Data. Lys, lysine. (TIF) [file pbio.3000135.s006.tif]

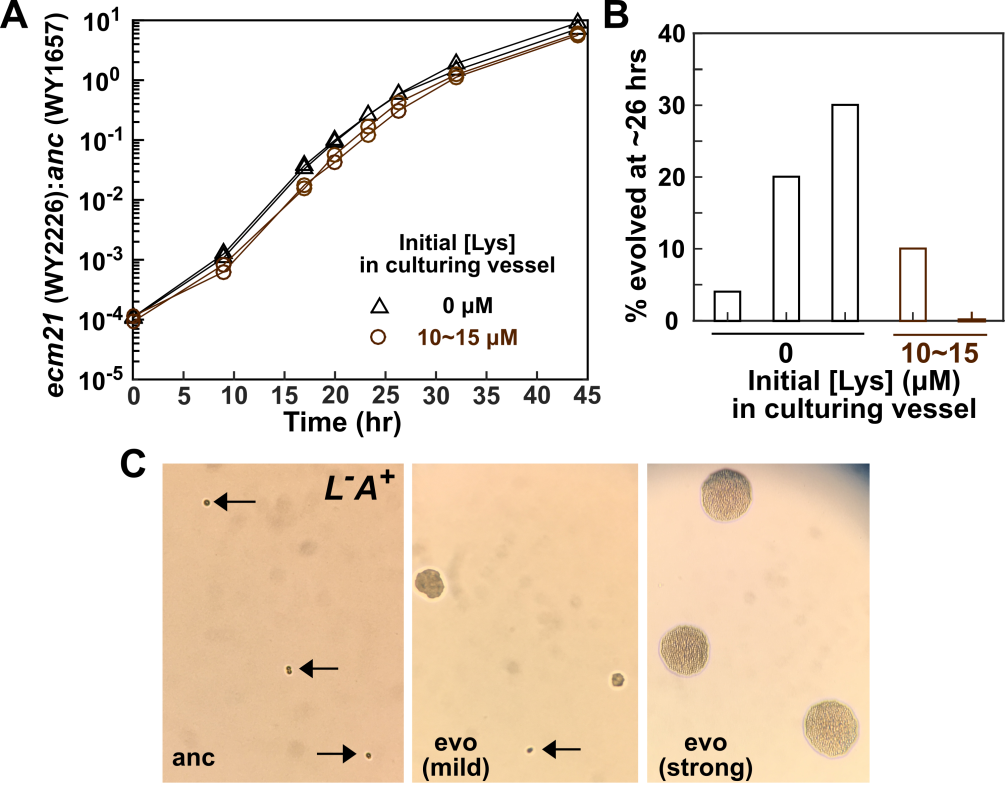

Supplement: S7 Fig — Whole-genome sequencing revealed that evolved L−A+ clones harbor mutations in genes such as RSP5 (an E3 ubiquitin ligase) and ECM21 (an arrestin-like adaptor for Rsp5) [43]. In a stressful environment, wild-type Ecm21 and Rsp5 proteins target cell surface permeases (including the high-affinity lysine permease, Lyp1) for ubiquitination [53]. Ubiquitinated permeases are then endocytosed and degraded in the vacuole [53]. The resulting amino acids are then transported to the cytoplasm for protein synthesis to help cells cope with stress [89]. In evolved cells with mutant ecm21 or rsp5, lysine permease is stabilized. (A) Evolved L−A+ grows faster than the ancestor in lysine-limited chemostats. L−A+ with or without an ecm21 deletion (WY2226 and WY1657, respectively) expressing different fluorescent proteins were competed in 8-h doubling time chemostats. The initial lysine concentrations in culturing vessels was 0 (black triangles) or 10–15 μM (brown circles). In all four chemostats, ecm21 overtook the ancestor. The fitness difference between the two strains can be estimated: Let E(t) and A(t) be population densities of ecm21 and the ancestor at time t, respectively, and let rE and rA be the growth rates of the two strains. Then, E(t)A(t)=E(0)A(0)⋅e(rE−rA)t, and we have ln(E(t)A(t))=ln(E(0)A(0))+(rE−rA)t. We quantified (rE−rA), the fitness advantage of ecm21 over ancestor, as 0.31/h (computed up to 32 h), compared with the ancestor growth rate of 0.087/h (8-h doubling). Thus, the growth rate of ecm21 is about 3.6-fold that of the ancestor in 8-h doubling chemostats. This fitness advantage is qualitatively consistent with what we observed in chemostats initiated with pure ancestor, because evolved clones increased from about 4% to about 40% within 5.7 h (from 26.3 to 32 h in Fig 3C), translating to a 0.49/h fitness difference. We infer that evolved clones are initially present at a frequency on the order of about 0.04/exp(0.4/h * 26.3 h) = 10−6. This is in line with the phe [file pbio.3000135.s007.tif]

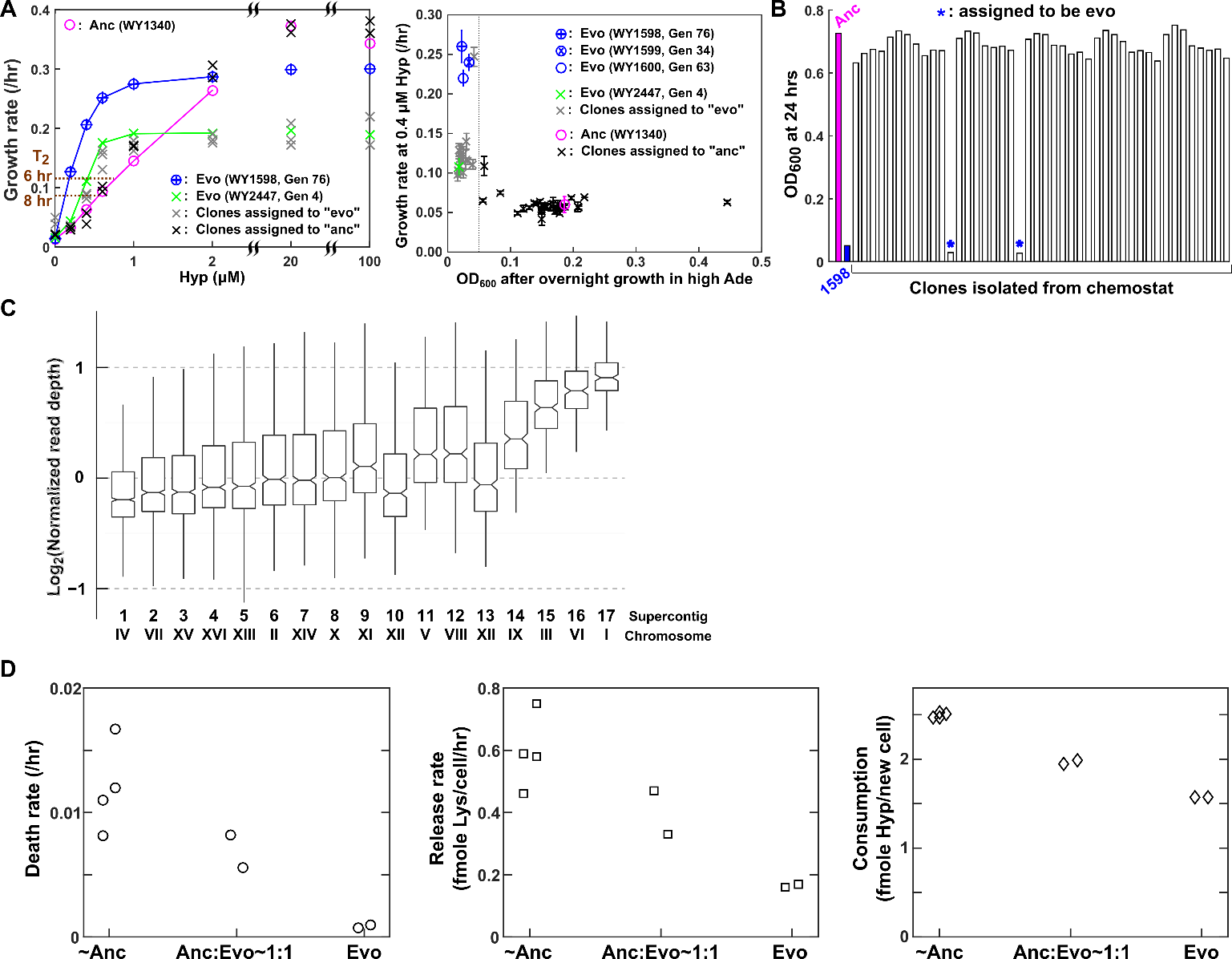

Supplement: S8 Fig — (A) A fitness trade-off in A−L+. (Left) Growth rates of ancestral (magenta) and evolved (blue and green) A−L+ clones (prestarved overnight) in various concentrations of hypoxanthine (Methods, “Microscopy quantification of growth parameters”) were plotted. Brown dotted lines mark 6-h and 8-h doubling time, a range experienced by CoSMO. In CoSMO, hypoxanthine concentrations were low (about 1 μM). Evolved clones grew faster than the ancestor under low hypoxanthine concentrations, but grew slower than the ancestor under high hypoxanthine concentrations (e.g., 20–100 μM). Clones marked by crosses were isolated from Generation 4 (hour 31) of chemostat culturing. (Right) A negative correlation between growth rate at low hypoxanthine versus turbidity in high adenine after overnight growth. Error bars on growth rate indicate the 95% confidence interval on slope (rate) estimation. Gray line indicates the threshold by which we differentiated evolved clones (left of gray line) from ancestral clones (right of gray line), according to the growth rate assay in the left panel. In both panels, gray crosses represent Generation 4 clones assigned to be evolved, while black crosses represent Generation 4 clones assigned to be ancestral. (B) A high-throughput assay that distinguishes ancestral from evolved A−L+ clones. We used turbidity after overnight growth in high Ade (108 μM) to classify A−L+ clones as ancestral (no blue stars) or evolved (blue stars). The ancestral clone (WY1340) and an evolved clone (WY1598) are shown as controls. (C) Aneuploidy in the evolved clone WY2447. Whole-genome sequencing revealed that, in addition to a synonymous nucleotide change, two nucleotide changes in noncoding regions and a point mutation from Cys102 to Ser in the gene OAR1 (S3 Table), Chromosomes I, III, and VI are likely duplicated. For Chromosome III, read depth was not fully twice that of other chromosomes, which could be caused by cells losing the extra copy of Chromosome III during culturing [file pbio.3000135.s008.tif]

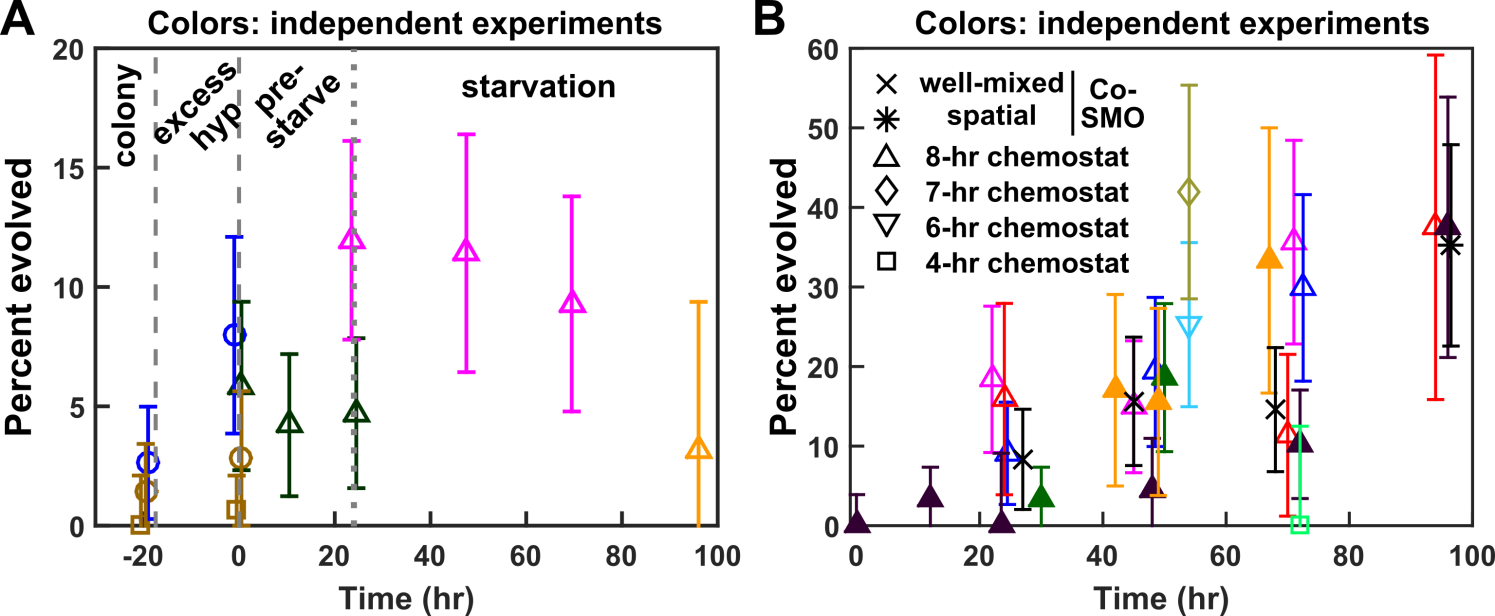

Supplement: S9 Fig — (A) High levels of evolved A−L+ clones prior to a starvation experiment (left of dotted line) and during a starvation experiment (right of dotted line). Different colors represent experiments on different days. In experiments that terminated at 0 h (circles and squares), an entire colony grown on rich YPD (circles) or minimal SD plus excess (100 μM) hypoxanthine (square) (-20 h) was resuspended in SD, and a fraction was used to inoculate SD plus excess hypoxanthine to grow exponential cultures (0 h). Otherwise (triangles), a fraction of the YPD-grown colony was used to inoculate SD plus excess hypoxanthine to grow exponential cultures, and at time zero, the culture was washed free of supplements and starved of hypoxanthine. (B) Similar percentages of evolved A−L+ clones in chemostats and in CoSMO. For chemostat experiments, exponentially growing cells washed free of supplements were prestarved (unfilled symbols) or not prestarved (filled symbols), and inoculated into chemostats (time zero). For CoSMO experiments, A−L+ cells were prestarved. In all experiments, we used the assay in S8B Fig to distinguish ancestral and evolved clones (Methods, “Detecting evolved clones”). If we sampled ntot cells, and nevo cells were evolved, then the fraction evolved was estimated to be nevo/ntot, with the error bar indicating 2nevontot (assuming that the random variable nevo followed a Poisson distribution). If zero assayed colonies were evolved, we identified the maximal frequency of evolved clones such that the error bar of 2nevontot still covered zero, and used that error bar. For example, if 0 out of 88 were evolved (0%), and because 3 out of 88 had a frequency of 3.4%, with an error bar of 3.9%, which covered zero, we added an error bar of 3.9% above the 0% data point. All data can be found in S15 Data. CoSMO, Cooperation that is Synthetic and Mutually Obligatory; SD, Synthetic Dextrose minimal medium; YPD, Yeast extract Peptone Dextrose rich medium. (TIF) [file pbio.3000135.s009.tif]

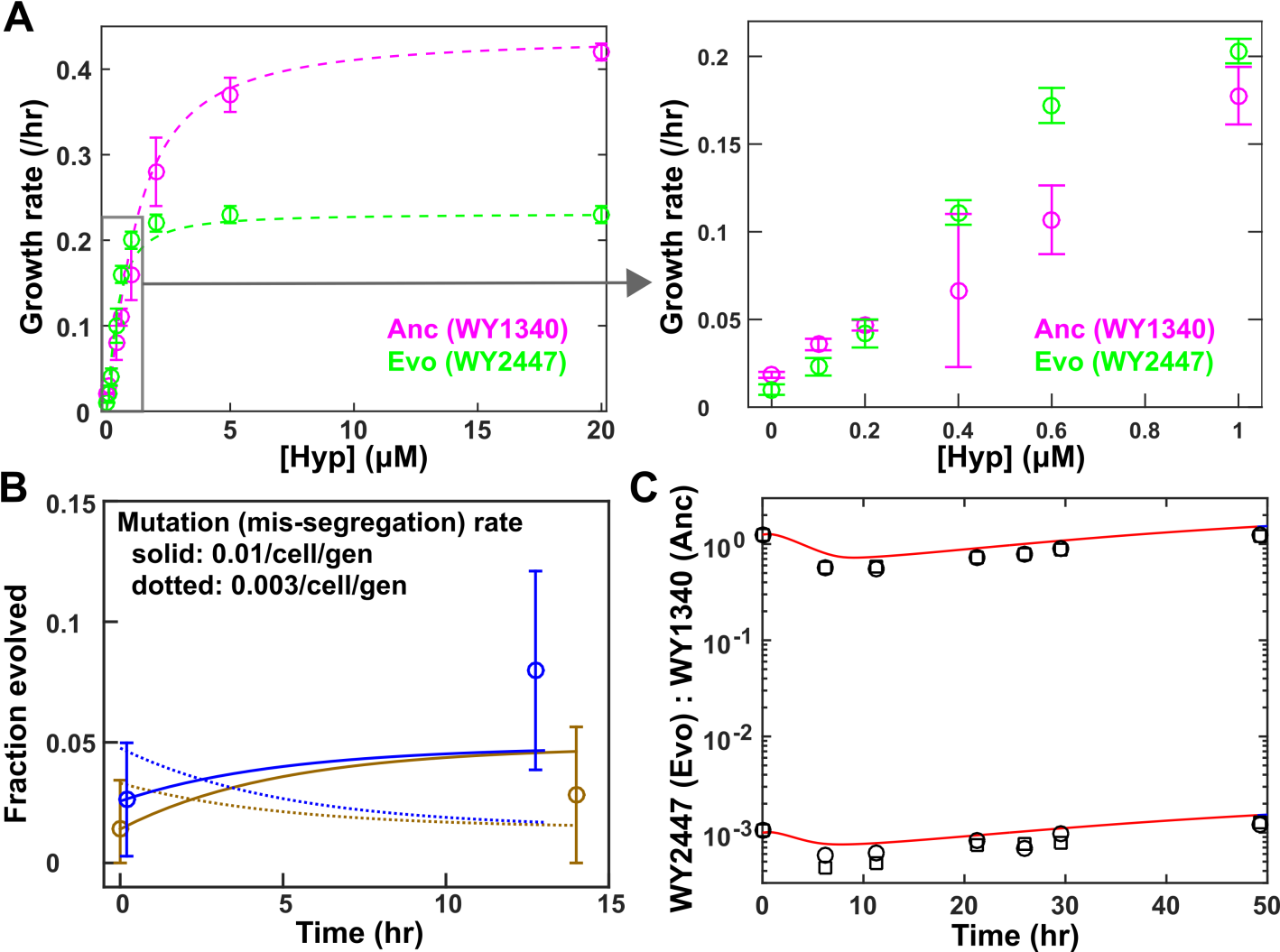

Supplement: S10 Fig — (A) Growth rates of ancestral (WY1340) and evolved (WY2447) A−L+ cells in various concentrations of hypoxanthine (24-h prestarvation; Methods, “Microscopy quantification of growth phenotypes”). Three experiments were averaged, and error bars indicate 2 standard deviations. (B) The evolutionary dynamics of A−L+ in excess hypoxanthine could be explained if we assumed that chromosome mis-segregation generated WY2447-like mutants at a rate of 0.01/cell/generation (solid lines). As a comparison, predictions from a mutation rate of 0.003/cell/generation (dotted lines) were also plotted. Brown and blue circles (measured in two different experiments) are identical to the corresponding ones in S9A Fig. Specifically, from the inoculum size and the final population size, we calculated the number of generations, which we then multiplied with the doubling time in SD with excess hypoxanthine to obtain the duration of the exponential phase. We then inferred the lag phase to be about 6 h and assumed that the fraction of evolved cells at time zero (the beginning of exponential phase) was similar to that at the time of inoculation. Our model (S2 Code) considered the fitness advantage of ancestor over mutant in excess hypoxanthine (A), as well as the conversion from ancestor to mutant. Data at 0 h were slightly jittered to aid visualization. (C) We competed WY2447 (expressing citrine fluorescent protein) and WY1340 (expressing green fluorescent protein) in 8-h chemostats from two starting ratios, and measured strain ratios over time using flow cytometry (black circles). Using a mathematical model (S3 Code) in which growth parameters were measured experimentally (A) and the ancestor converted to WY2447-like mutants at a rate of 0.01/cell/generation, we obtained a qualitative matching between model and experiments. In both models (B, C), death rate and hypoxanthine consumption per birth were from 8-h chemostat measurements (Methods, “Quantifying phenotypes in chemostats”). All data can [file pbio.3000135.s010.tif]

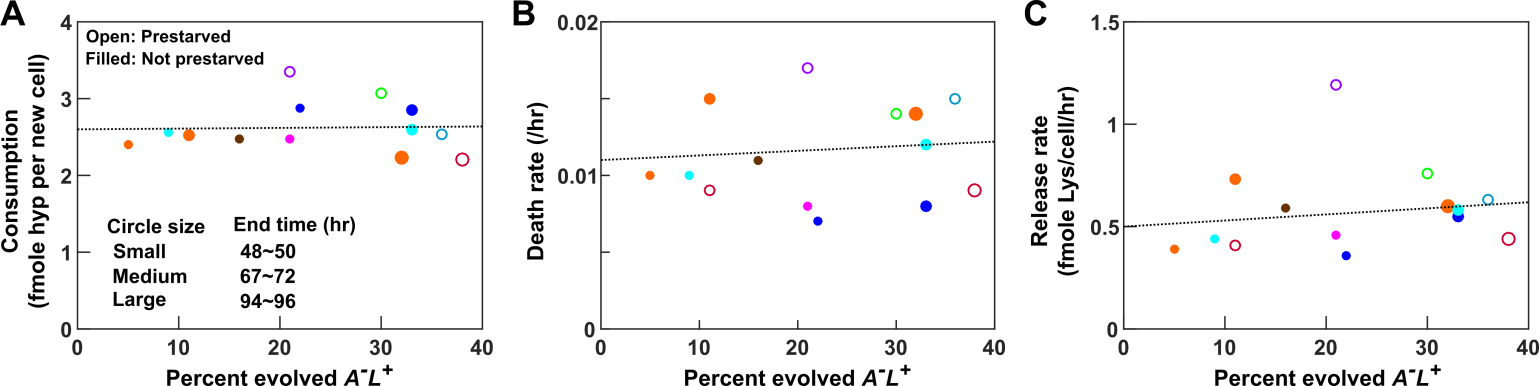

Supplement: S11 Fig — A−L+ (WY1340), either prestarved for 24 h (open circles) or not prestarved (filled circles), were cultured in 8-h chemostats (different colors representing independent chemostat experiments). Hypoxanthine consumption per birth (A), death rate (B), and lysine release rate (C) were quantified (Methods, “Quantifying phenotypes in chemostats”) using dynamics up to 48–50 h (small-sized circles), 67–72 h (medium-sized circles), or 94–96 h (large-sized circles). Percentage of evolved clones was quantified at the end of each measurement. Despite phenotypic differences between ancestral and evolved A−L+ (S8D Fig), measured phenotypes did not show significant correlation with percentage evolved (slope ± standard error of the mean [SEM] being 0.1 ± 0.8 (A), 0.003 ± 0.008 (B), and 0.3 ± 0.6 (C)—none significantly different from zero). This lack of correlation is presumably due to the relatively large measurement errors and the relatively narrow spread in percentage evolved. Take consumption as an example. Suppose that ancestral and evolved A−L+ consumed hypoxanthine at 2.5 fmole/birth and 1.5 fmole/birth, respectively (S8D Fig). At 10% mutants, consumption would be 2.5 * 0.9 + 1.5 * 0.1 = 2.4 fmole/birth. At 30% mutants, consumption would be 2.5 * 0.7 + 1.5 * 0.3 = 2.2 fmole/cell. This 10% difference is smaller than the measurement error. For example, at about 33% evolved A−L+ (filled dots in A), consumption varied from 2.2 to 2.8 fmole/birth. In summary, quantified phenotypes did not correlate strongly with percentage mutants because percentage mutants were sufficiently similar across different replicates and because measurement errors were sufficiently large. All data can be found in S17 Data. (TIF) [file pbio.3000135.s011.tif]

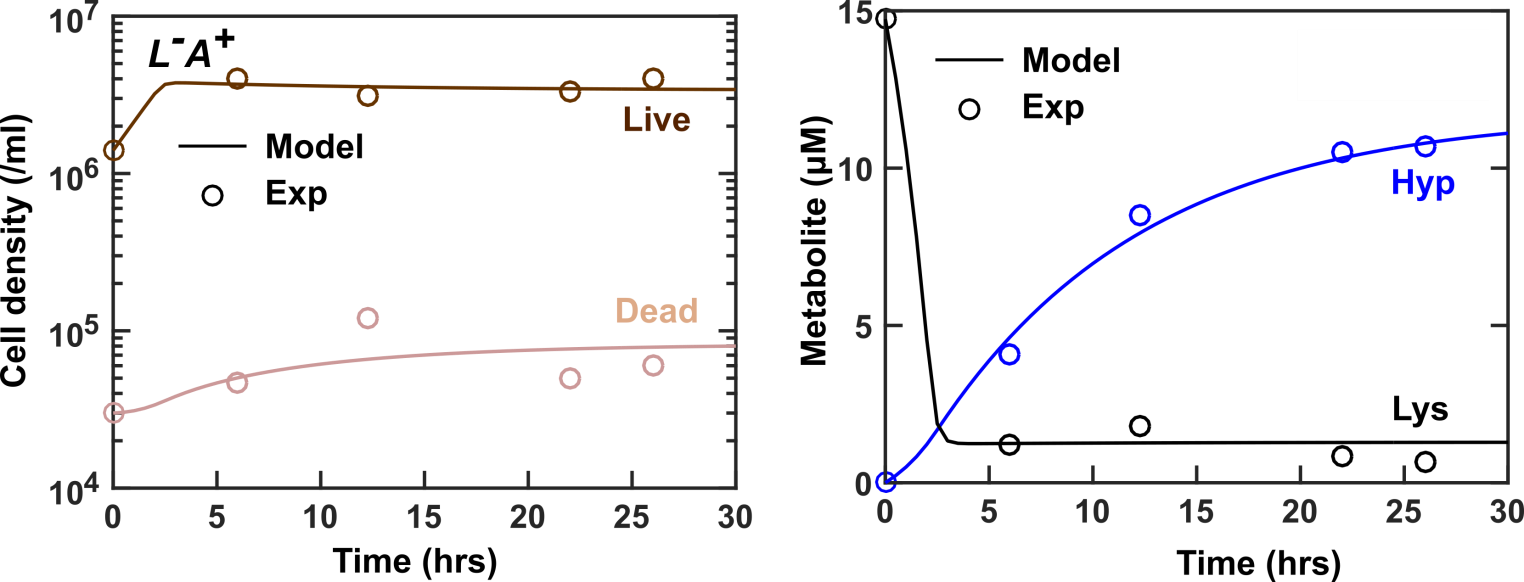

Supplement: S12 Fig — L−A+ cells in SD + 15 μM lysine were inoculated into a chemostat culturing vessel (19 mL). SD + 20 μM lysine in the reservoir was pumped into the culturing vessel to achieve an 8-h doubling time (i.e., 19 mL * ln(2)/8/h = 1.646 mL/h). L−A+ phenotypes in Table 1 (except for release rate of 0.30 fmole hypoxanthine/cell/h and death rate of 0.0021/h measured in this particular experiment) were used to simulate chemostat dynamics (S4 Code). Simulations (lines) and experiments (circles) are in good agreement. All data can be found in S18 Data. SD, Synthetic Dextrose minimal medium. (TIF) [file pbio.3000135.s012.tif]

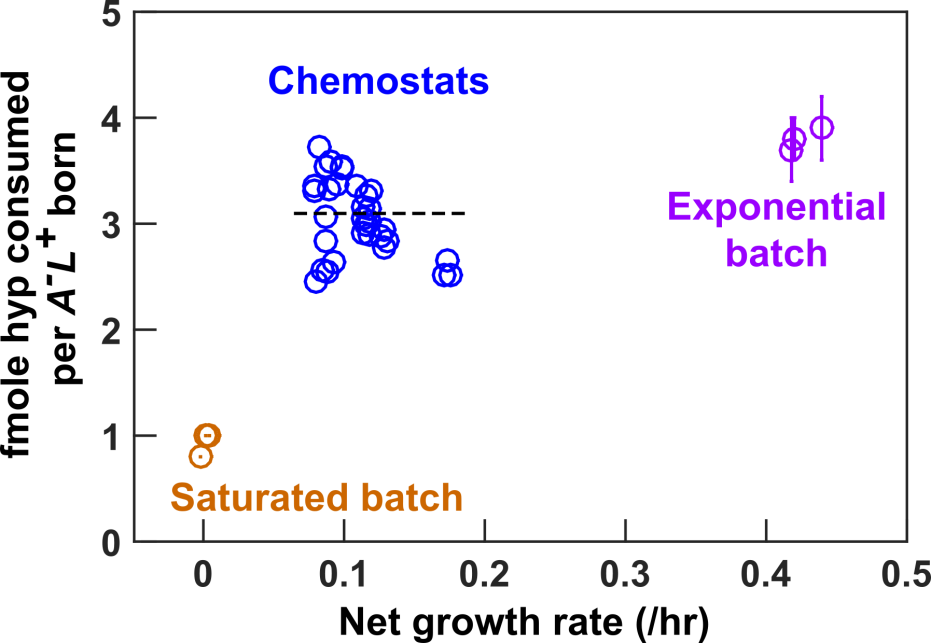

Supplement: S13 Fig — For hypoxanthine-limited chemostat measurements, data were jittered slightly along the x axis to facilitate visualization. Consumption was measured over a similar time window as that of CoSMO growth rate to ensure similar evolutionary effects. For exponential and saturation consumption of adenine (which is similar to hypoxanthine, see S3A Fig), error bars mark 2 SEMs for slope estimation. The black dashed line marks the average hypoxanthine consumption per A−L+ birth in chemostats (Table 1; data can be found in S6 Table). CoSMO, Cooperation that is Synthetic and Mutually Obligatory; SEM, standard error of the mean. (TIF) [file pbio.3000135.s013.tif]

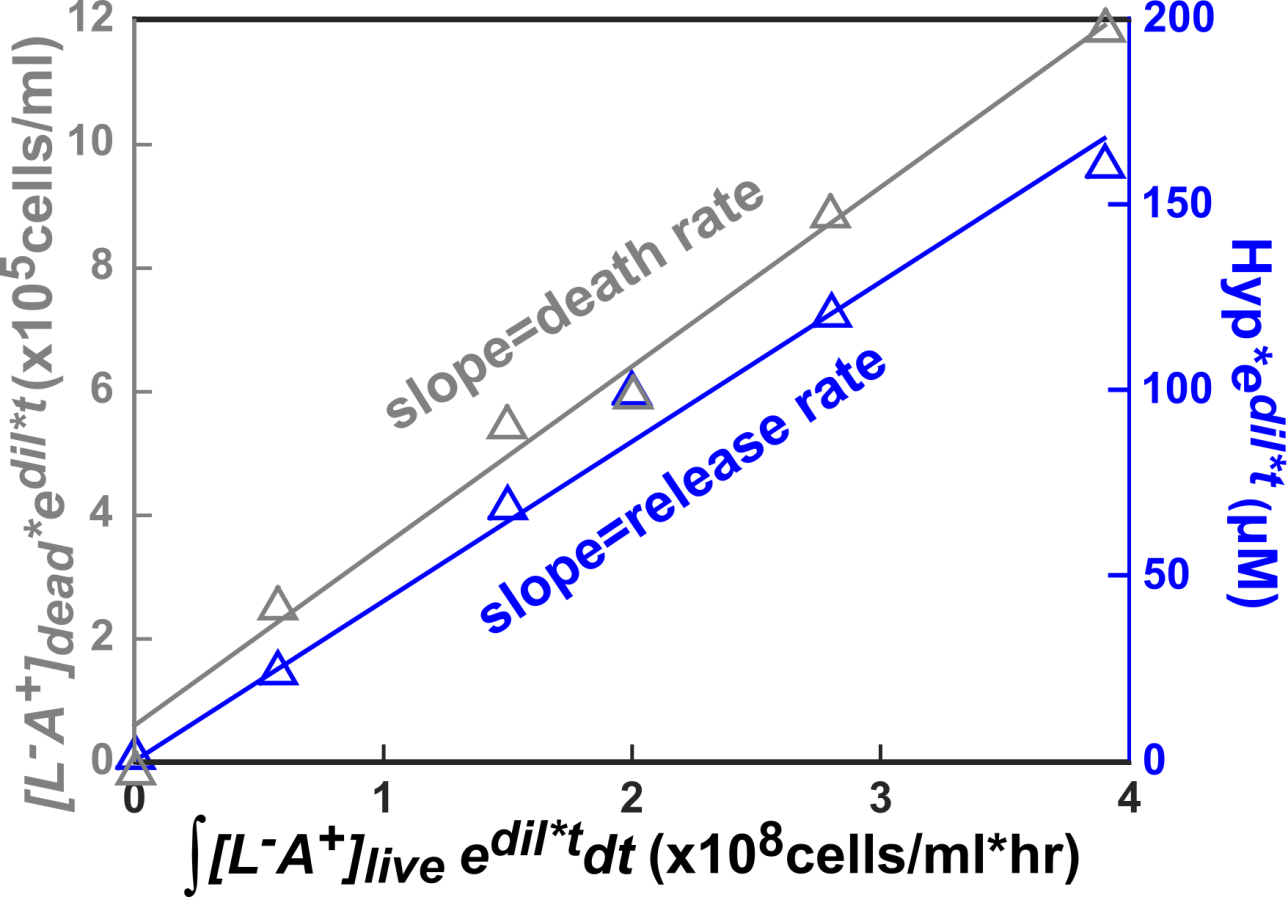

Supplement: S14 Fig — We used regression to measure death rate (gray) and hypoxanthine release rate (blue) for the triangle-marked chemostat experiment from Fig 3. For an explanation, see “Quantifying phenotypes in chemostats” in Methods. Densities of fluorescent live cells and nonfluorescent/ToPro3-positive dead cells were measured via flow cytometry (Methods, “Flow cytometry”). Hypoxanthine was quantified using the yield bioassay (Methods, “Bioassays”). All data can be found in S19 Data. (TIF) [file pbio.3000135.s014.tif]

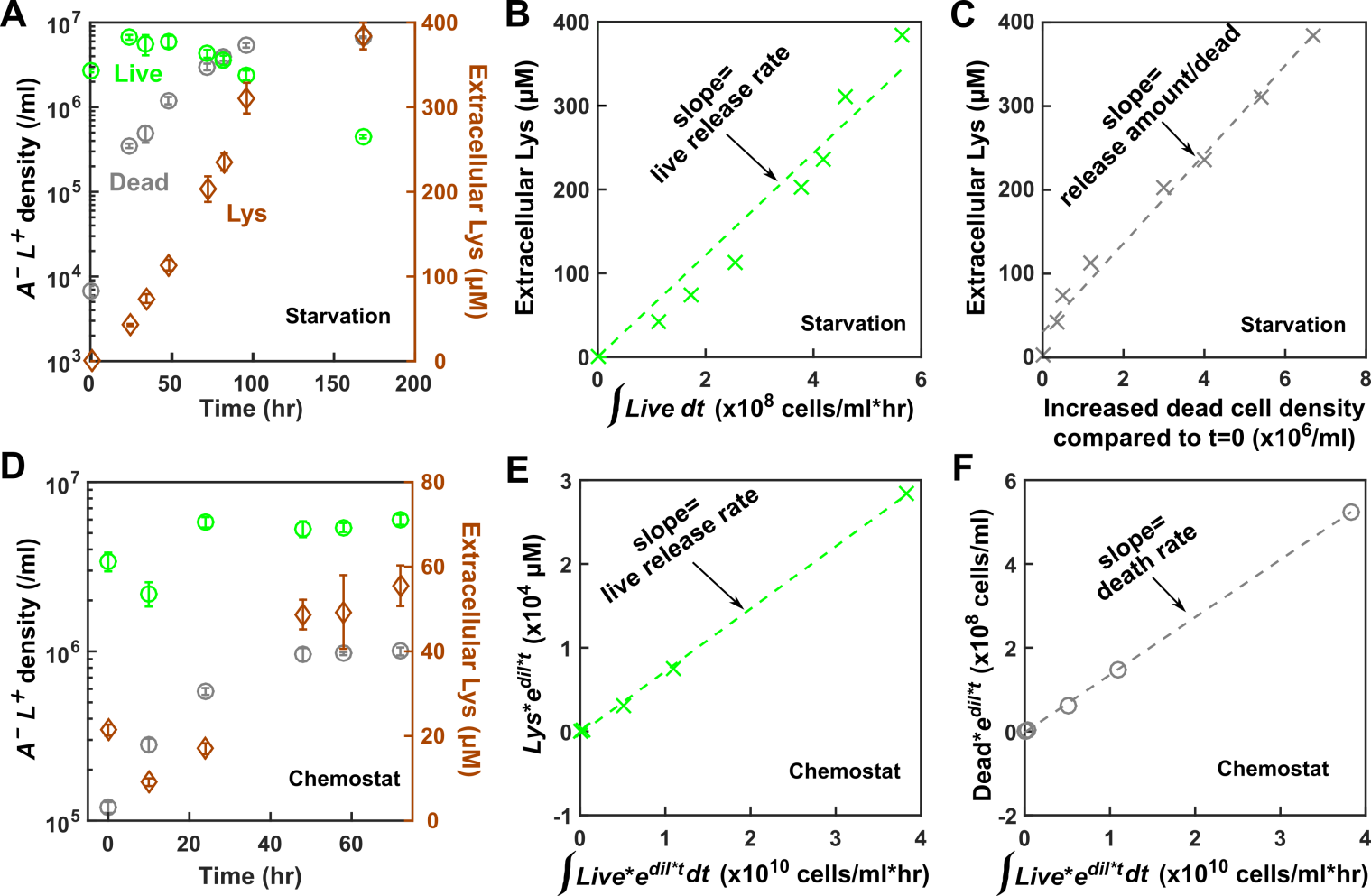

Supplement: S15 Fig — A−L+ cells grown to exponential phase in SD + excess hypoxanthine were washed and diluted into SD. (A-C) “Starvation”: at time zero, cells were inoculated into SD. From population and lysine dynamics (A), the live release model (B) and the dead release model (C) yielded similar fits to the data. Thus, from regression alone, we could not distinguish live from dead release. (D-F) “Chemostat”: cells were prestarved in SD for 24 h and then transferred to a hypoxanthine-limited chemostat (doubling time, 8 h) at time zero. From population and lysine dynamics (D), lysine release rate by live cells (E) and death rate (F) can be calculated from slopes of respective regressions (Methods, “Quantifying phenotypes in chemostats”). Note that lysine release rate during starvation (B) remained relatively constant during the initial 90 h (excluding the last two data points; the time window we later used to measure CoSMO growth rate; also see S18B Fig). In chemostat, upon reaching the steady state, the release rate also remained relatively constant (the last three time points in D, E). All data can be found in S20 Data. CoSMO, Cooperation that is Synthetic and Mutually Obligatory; SD, Synthetic Dextrose minimal medium. (TIF) [file pbio.3000135.s015.tif]

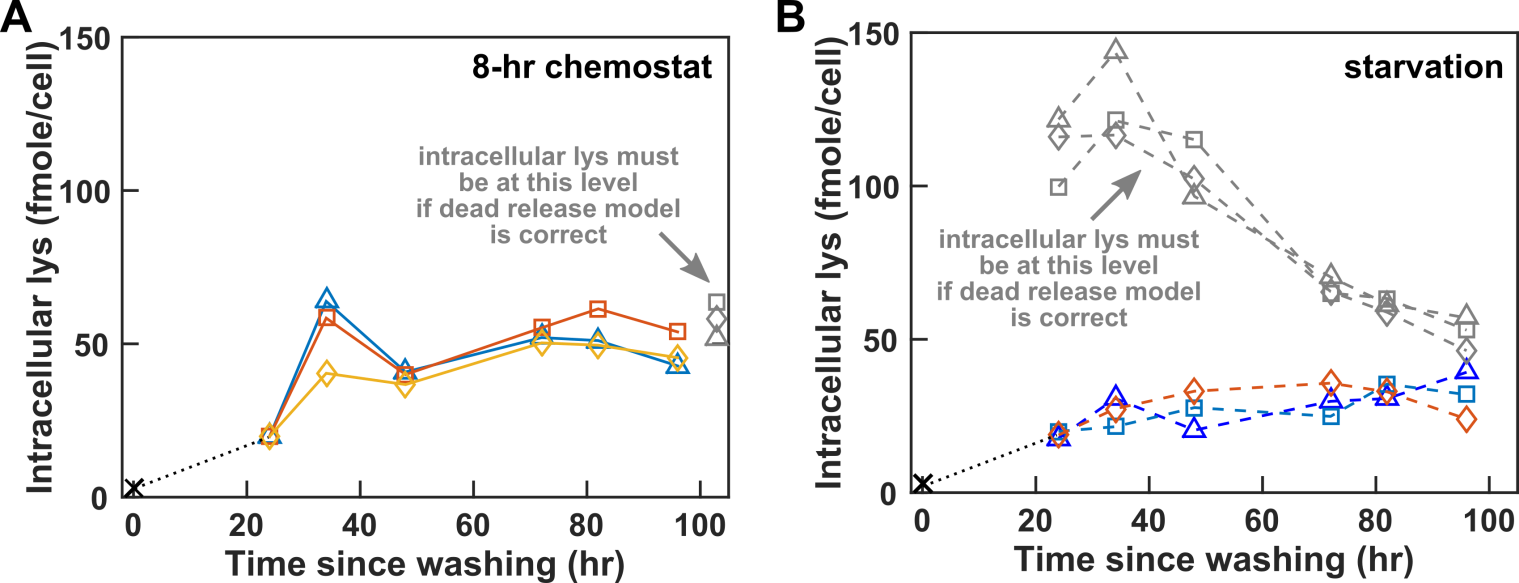

Supplement: S16 Fig — A−L+ cells (WY1340) grown in SD + excess hypoxanthine to exponential phase were washed and diluted into SD at time zero. Cells were either starved further (B) or inoculated into hypoxanthine-limited chemostats after 24 h of prestarvation (A). At various times, cells were harvested, and intracellular lysine was extracted and measured via yield bioassay. Different colors (except gray) represent different replicates and are identical to those in Fig 6A. Gray symbols in (A) represent the intracellular lysine content required to satisfy the dead release model at the steady state (calculated from the last three time points of S15D Fig). Gray symbols and dashed lines in (B) represent the intracellular lysine content required to satisfy the dead release model during starvation. Because live or dead release could explain chemostat results (A) but dead release could not explain starvation results (B), we made the most parsimonious assumption of live release. All data can be found in S21 Data. SD, Synthetic Dextrose minimal medium. (TIF) [file pbio.3000135.s016.tif]

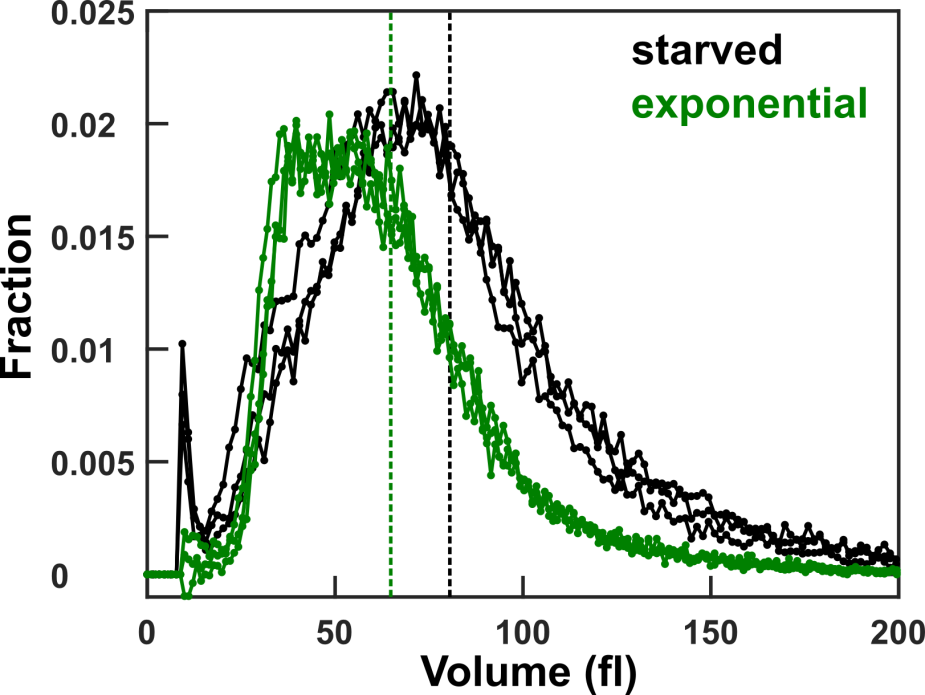

Supplement: S17 Fig — A−L+ cells (WY1340) grown in SD + excess hypoxanthine were either maintained at exponential phase (green) or washed and starved in unsupplemented SD for 24 h (black). Cell sizes were measured using a Coulter counter. The initial peak in starved cells may represent dead cell debris. The average sizes of exponential and starved cells were 64.8 fL and 80.5 fL, respectively (dashed lines). All data can be found in S22 Data. SD, Synthetic Dextrose minimal medium. (TIF) [file pbio.3000135.s017.tif]

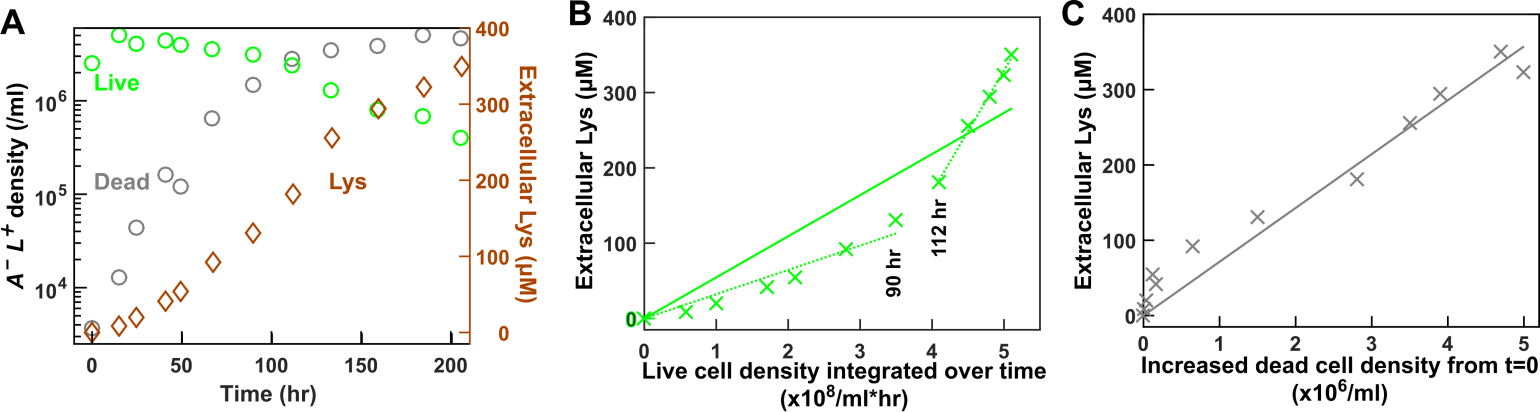

Supplement: S18 Fig — (A) Exponentially growing A−L+ cells were washed and diluted into SD. Live and dead population densities were measured by flow cytometry, and lysine concentration was measured by the yield bioassay. Regression in both the live release model (B) and dead release model (C) deviated from linearity. Because metabolite analysis suggests that live release is more likely (S16B Fig), we infer that the release rate is time variant—initially slow and then speeding up (a similar but less obvious trend can also be seen in S15B Fig). However, because CoSMO growth rate measurements rarely exceeded 96 h, we used the lysine release rate measured up to 90 h in Model ii. All data can be found in S23 Data. CoSMO, Cooperation that is Synthetic and Mutually Obligatory; SD, Synthetic Dextrose minimal medium. (TIF) [file pbio.3000135.s018.tif]

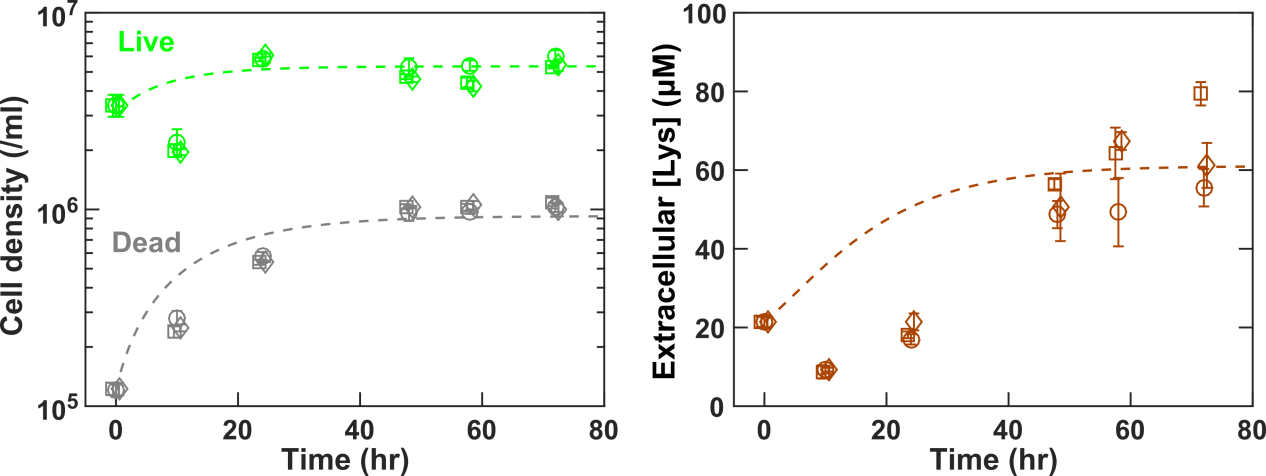

Supplement: S19 Fig — A−L+ cells grown exponentially in SD + excess hypoxanthine were washed and diluted into SD, and prestarved for 24 h. At time zero, starved cells (together with the medium, which had already accumulated some lysine) were inoculated into chemostats, and fresh SD + 20 μM hypoxanthine was pumped in at a rate to achieve a doubling time of 8 h. Dynamics of live and dead populations (left) and of released lysine (right) were plotted (squares, circles, and diamonds representing three chemostats). The model (dashed lines; S5 Code) was based on parameters in Table 1, except for a lysine release rate of 0.99 fmole/cell/h, which was averaged among the three chemostats. The initial decline in live cell density in experiments was presumably due to a growth lag when cells transitioned from starvation to chemostats, which was not modeled. The initial decline in extracellular lysine concentration in experiments is consistent with the live release model: reduced live cell density leads to reduced extracellular lysine. All data can be found in S24 Data. SD, Synthetic Dextrose minimal medium. (TIF) [file pbio.3000135.s019.tif]

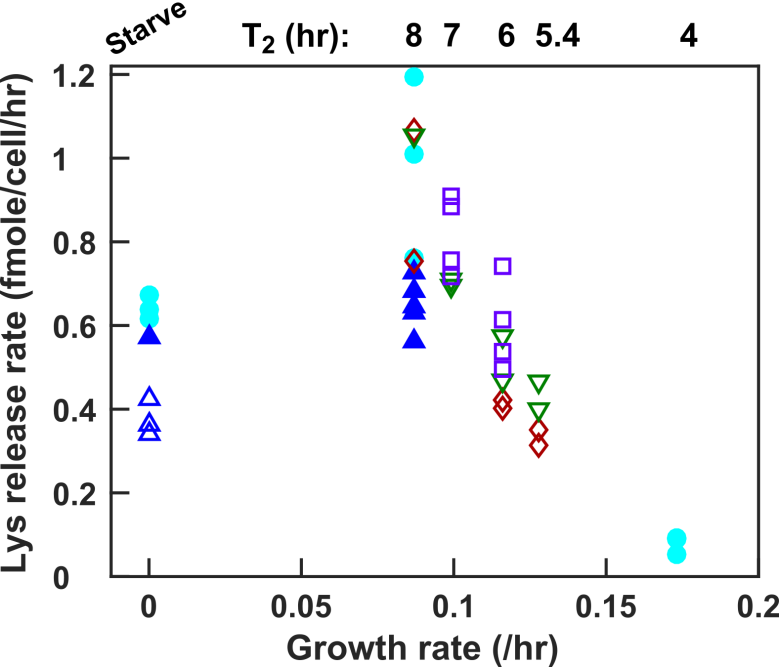

Supplement: S20 Fig — A−L+ cells grown to exponential phase in SD + excess hypoxanthine were washed and diluted into SD at time zero. Cells were either starved further (“Starve”) or inoculated into hypoxanthine-limited chemostats after 24 h of prestarvation (e.g., S15D and S15E Fig; doubling times marked above). Each symbol represents an independent measurement, and measurements done at the same time were marked with the same color. Open and closed symbols represent pregrowth done in tubes versus flasks, respectively. We observed day-to-day variations in measurements (e.g., cyan circles higher than blue triangles). Lysine release rate data are in S8 Table. SD, Synthetic Dextrose minimal medium. (TIF) [file pbio.3000135.s020.tif]

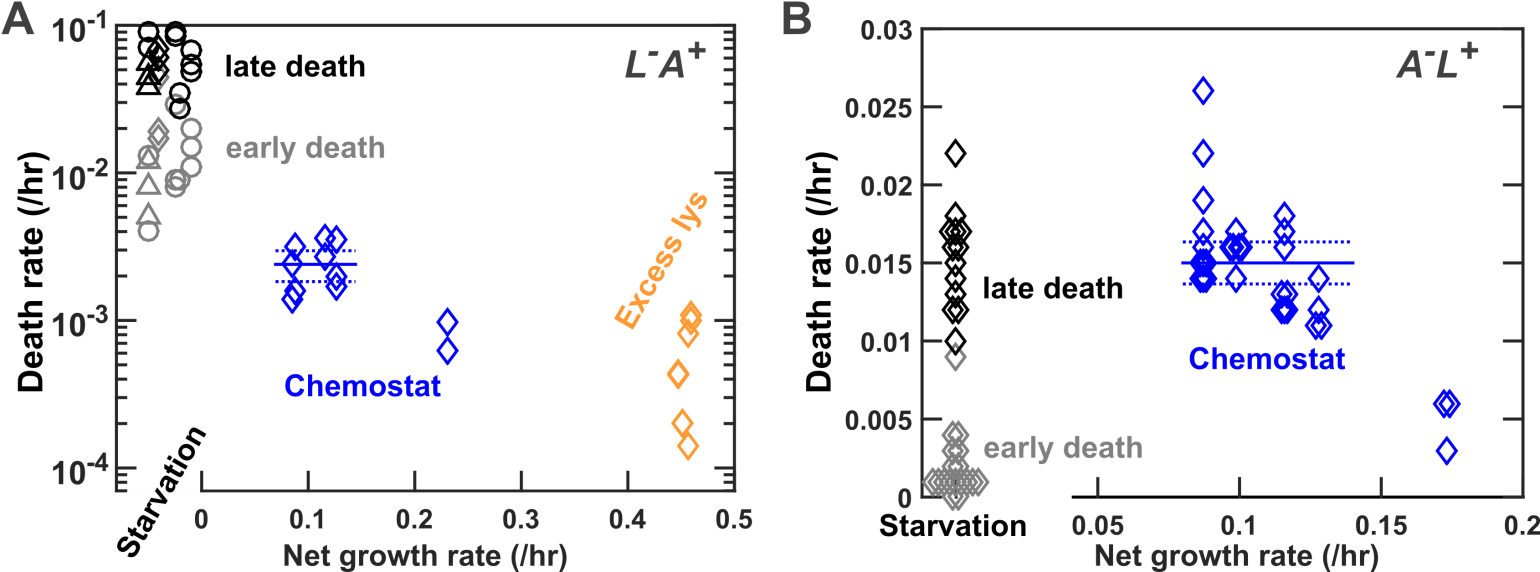

Supplement: S21 Fig — (A) Exponential L−A+ (WY1335) cells were washed in SD, and death rate was measured in chemostats at various doubling times (Methods, “Quantifying phenotypes in chemostats,” Eq 16; S14 Fig, gray). As a comparison, death rates in batch cultures with zero or excess lysine are shown (see [27] for detailed methodology and data). With no lysine, the early-phase death rate (gray; from 5 to 12 h post-wash) was slower than the late-phase death rate (black; from 12 to 30 h post-wash). With excess lysine, death rates were very low (orange diamonds; Methods, “Calculating death rate in nonlimited batch culture”). Death rates of L−A+ in chemostats (blue; doubling times from left to right being 8, 6, 5.5, and 3 h) were in between death rates in starvation and in excess lysine. Blue solid and dashed lines mark the mean death rate ±2 SEMs from 5.5–8-h doubling time chemostats (Table 1). Detailed data for A are in S7 Table. We used a log plotting scale to visualize differences between small numbers. (B) Exponential A−L+ (WY1340) cells were washed and prestarved for 24 h. They were either further starved (black and gray) or cultured at various growth rates in chemostats (blue). During starvation, death rate was initially slow (gray) and then sped up (black; see [27] for detailed methodology and data). Average death rate (blue solid line) and 2 SEM (blue dotted lines) were calculated from chemostats run at doubling times of 5.4–8 h (e.g., S15F Fig) and used in our model (Table 1). We used a linear plotting scale because early death rates were zero and could not be plotted on the log scale. In both A and B, death rates were quantified from the decline rate of ln(live population size), and live population size could be measured via microscopy total fluorescence intensity [27] (circles), microscopy live cell count [27] (triangles), or flow cytometry live cell density (diamonds; Methods, “Flow cytometry”). Detailed data for B are in S8 Table. CoSMO, Cooperation that is Synthetic and Mutual [file pbio.3000135.s021.tif]

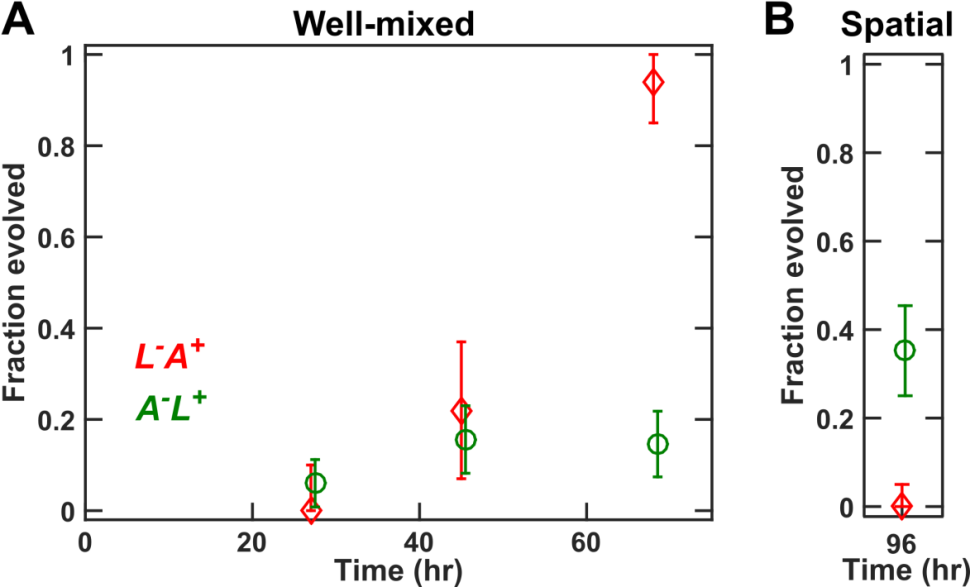

Supplement: S22 Fig — (A) L−A+ evolves rapidly in a well-mixed environment. Exponentially growing L−A+ (WY1335) and A−L+ (WY1340) were washed free of supplements, preconditioned, and mixed at 1:1 in SD at a total cell density of 105/mL. The resultant CoSMO was grown in a well-mixed environment. At various times, samples were plated on YPD, and 32 L−A+ colonies (red diamonds) and 84–96 A−L+ colonies (green circles) were isolated to assay whether they were evolved or not (Methods, “Detecting evolved clones”). (B) L−A+ evolves slowly in a spatially structured environment. L−A+ (WY1335 and WY1657) and A−L+ (WY1340 and WY1342) were mixed at approximately equal ratio and spotted onto the middle of an agarose slice containing 0.7 μM lysine (the spotting setting in Methods, “Quantifying spatial CoSMO growth dynamics”; prestarved A−L+ cells were washed again in SD so that CoSMO started with a defined level of lysine in the agarose pad). At 96 h, CoSMO samples were plated on YPD, and 80 L−A+ colonies (red diamonds) and 88 A−L+ colonies (green circles) were isolated to assay whether they were evolved or not. For (A) and (B), error bars indicate 2 standard deviations according to binomial distribution. Specifically, if we observed e evolved clones among N total clones, then the fraction evolved was p = e/N and the error bar was 2[Np(1−p)]/N. If no evolved clones were observed, then p = 0, and the upper error bar was defined to be that of maximal e, whose lower error bar spanned 0 (similar to S9 Fig). Error bars were truncated at 0 and 1. All data can be found in S25 Data. CoSMO, Cooperation that is Synthetic and Mutually Obligatory; SD, Synthetic Dextrose minimal medium; YPD, Yeast extract Peptone Dextrose rich medium. (TIF) [file pbio.3000135.s022.tif]

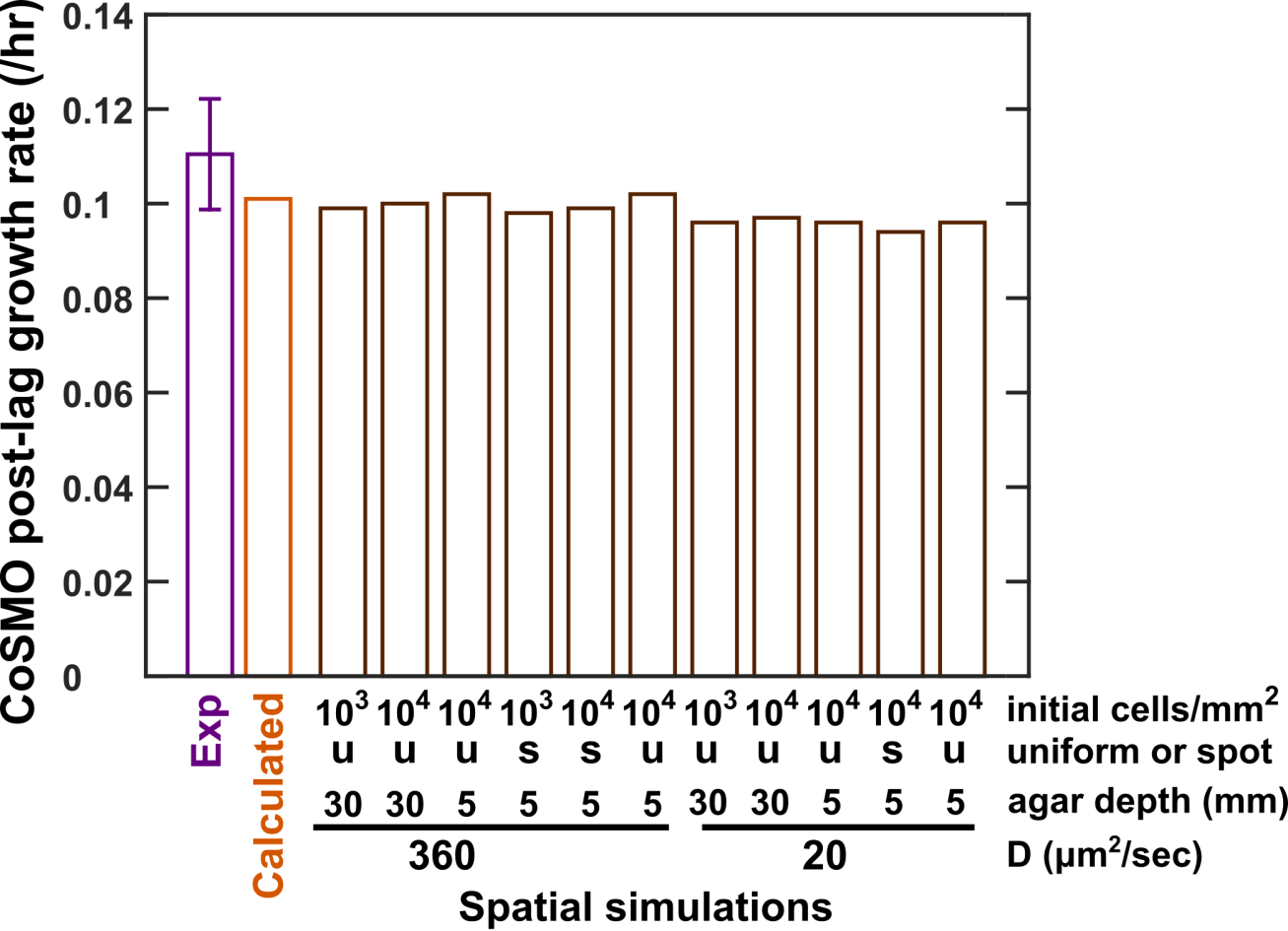

Supplement: S23 Fig — Spatial CoSMO growth was simulated under varying initial total cell density, inoculation setup (uniformly plated “u” versus centrally spotted “s”), agar depth, and diffusion coefficient (20 and 360 μm2/s, corresponding to diffusion coefficients in community and agarose, respectively [32]). Spatial simulations yielded similar CoSMO growth rates (brown). Experimental measurements of spatial CoSMO (purple) and CoSMO growth rate calculated from Eq 17 (orange) were taken from Fig 7 and plotted here for comparison. The spatial model and the calculation both considered variable lysine release rate. All data can be found in S26 Data. CoSMO, Cooperation that is Synthetic and Mutually Obligatory. (TIF) [file pbio.3000135.s023.tif]

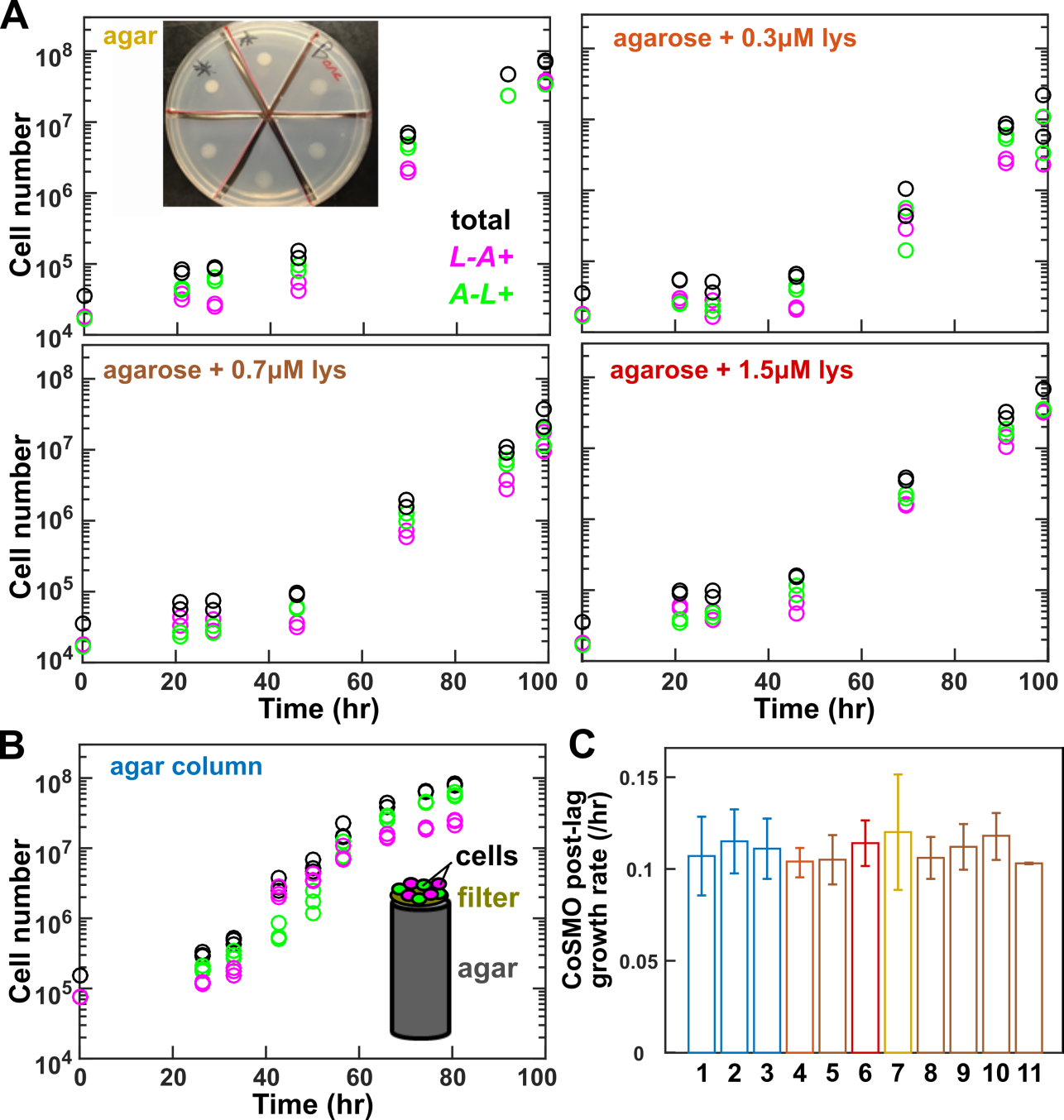

Supplement: S24 Fig — Preconditioned L−A+ and A−L+ were mixed at approximately 1:1 ratio and grown on 2 × SD agar (which may contain trace nutrient contaminants) or agarose (Methods, “Quantifying spatial CoSMO growth dynamics”). (A) Growth dynamics of CoSMO on four media. Inset: shared experimental setup. A total of 15 μL of 4 × 10^4^ total cells was spotted on the center of the cut pad, forming an inoculum spot of radius 4 mm. The lower left panel is identical to Fig 7A. (B) Growth dynamics of CoSMO in deep 96-well plates. A total of 1.5 × 10^5^ initial total cells were filtered on top of a membrane filter to ensure uniform spatial distribution. This was equivalent to 3,000 cells/mm2. (C) After the lag phase, steady-state growth rates of CoSMO were calculated from 11 independent experiments, with color-coding corresponding to conditions in (A) and (B). Time points at which total cell numbers exceed 1 × 10^8^ were excluded to avoid stationary phase. Error bars mark 2 standard errors of estimating growth rate. In A and B, each data point represented the average of three flow cytometry measurements of a single spatial sample. Experimental data for A and B and summary data for C are provided in S4 Table. CoSMO, Cooperation that is Synthetic and Mutually Obligatory; SD, Synthetic Dextrose minimal medium. (TIF) [file pbio.3000135.s024.tif]

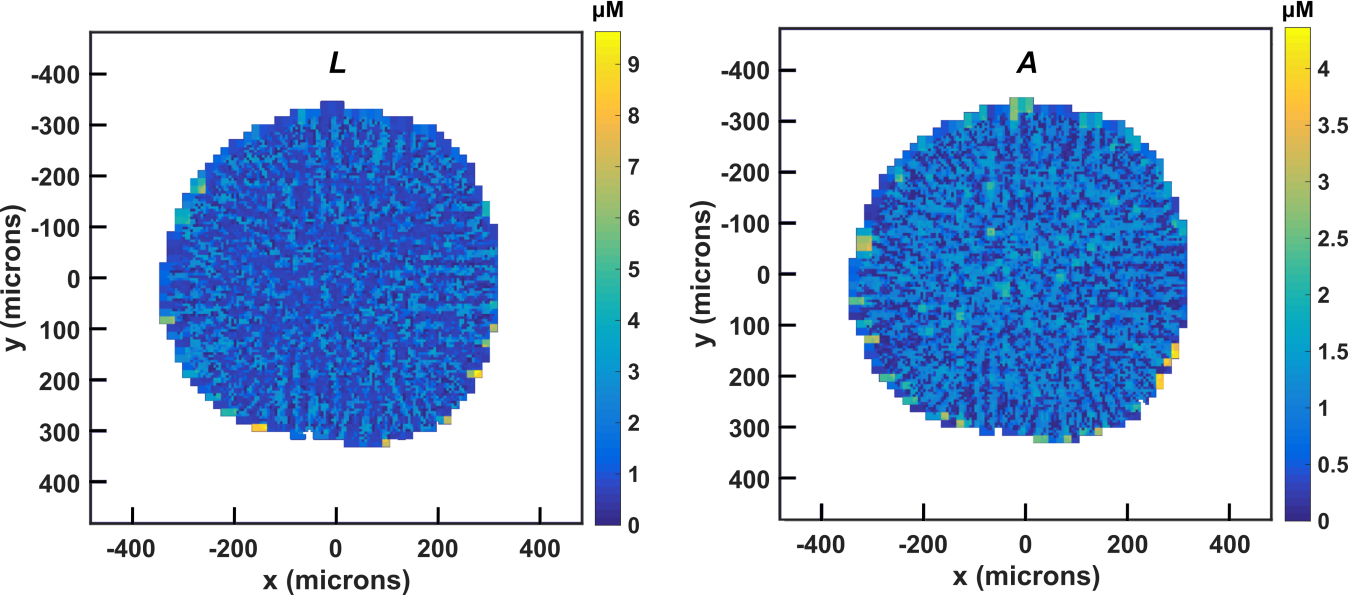

Supplement: S25 Fig — Metabolite concentrations in the agarose and the community eventually reach a nearly uniform state. Plotted are top views of lysine (L, left panel) and hypoxanthine (A, right panel) concentrations in the cell layer immediately on top of the agarose surface 120 h after being spotted in the middle of an agarose pad. Because the populations were fairly intermixed within the community [32], the overall metabolite distributions remained fairly uniform within the community. The spatial averages of L and A in the community were 1.35 μM and 0.79 μM, respectively. The average concentrations in the agarose (1.31 μM for L and 0.73 μM for A) closely matched those inside the community. Thus, the CoSMO growth rate in a spatially structured environment is similar to that in a well-mixed environment. Here, the diffusion coefficients inside CoSMO and agarose were 20 and 360 μm2/s, respectively. CoSMO, Cooperation that is Synthetic and Mutually Obligatory. (TIF) [file pbio.3000135.s025.tif]

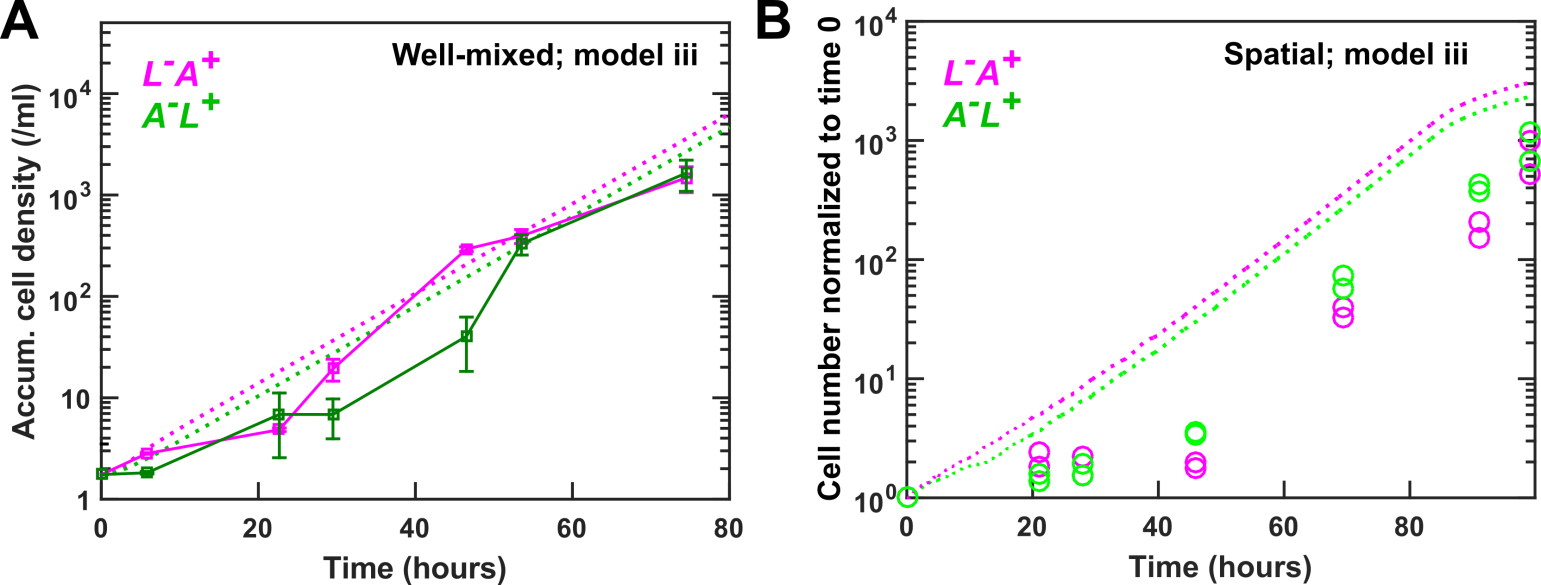

Supplement: S26 Fig — (A) Predicted (dotted lines; S8 Code) and measured (symbols; identical to those in Fig 1B) dynamics of a well-mixed community. (B) Predicted (dotted lines; S7 Code) and measured (symbols; identical to those in Fig 7A) dynamics of a community grown on top of an agarose pad. In B, the growth slowdown toward the end of simulations is due to exhaustion of shared nutrients in the agarose pad. All parameters are from Table 1. (TIF) [file pbio.3000135.s026.tif]
